# Tending to the machine: The impact of intrapartum fetal surveillance on women in Australia

**DOI:** 10.1371/journal.pone.0303072

**Published:** 2024-05-09

**Authors:** Deborah Fox, Rebecca Coddington, Kate M. Levett, Vanessa Scarf, Kerry L. Sutcliffe, Elizabeth Newnham

**Affiliations:** 1 Collective for Midwifery, Child and Family Health (CMCFH), University of Technology Sydney, NSW, Australia; 2 School of Medicine, University of Notre Dame Australia, Sydney, NSW, Australia; 3 NICM Health Research Institute and THRI, Western Sydney University, Penrith, NSW, Australia; 4 School of Nursing and Midwifery, University of Newcastle, Callaghan, NSW, Australia; Rural Women’s Social Education Centre, INDIA

## Abstract

Qualitative research about women and birthing people’s experiences of fetal monitoring during labour and birth is scant. Labour and birth is often impacted by wearable or invasive monitoring devices, however, most published research about fetal monitoring is focused on the wellbeing of the fetus. This manuscript is derived from a larger mixed methods study, ‘WOmen’s Experiences of Monitoring Baby (The WOMB Study)’, aiming to increase understanding of the experiences of women and birthing people in Australia, of being monitored; and about the information they received about fetal monitoring devices during pregnancy. We constructed a national cross-sectional survey that was distributed via social media in May and June, 2022. Responses were received from 861 participants. As far as we are aware, this is the first survey of the experiences of women and birthing people of intrapartum fetal monitoring conducted in Australia. This paper comprises the analysis of the free text survey responses, using qualitative and inductive content analysis. Two categories were constructed, *Tending to the machine*, which explores participants’ perceptions of the way in which clinicians interacted with fetal monitoring technologies; and *Impressions of the machine*, which explores the direct impact of fetal monitoring devices upon the labour and birth experience of women and birthing people. The findings suggest that some clinicians need to reflect upon the information they provide to women and birthing people about monitoring. For example, freedom of movement is an important aspect of supporting the physiology of labour and managing pain. If freedom of movement is important, the physical restriction created by a wired cardiotocograph is inappropriate. Many participants noticed that clinicians focused their attention primarily on the technology. Prioritising the individual needs of the woman or birthing person is key to providing high quality woman-centred intrapartum care. Women should be provided with adequate information regarding the risks and benefits of different forms of fetal monitoring including how the form of monitoring might impact her labour experience.

## Introduction

Intrapartum fetal surveillance refers to the continuous or intermittent monitoring of the fetus during labour with the intent to assess fetal wellbeing and detect signs of distress or compromise [1]. There are two main methods of intrapartum fetal surveillance: continuous electronic fetal monitoring (CEFM) and intermittent auscultation (IA). CEFM involves the use of electronic devices to monitor the fetal heart rate and uterine contractions continuously throughout labour [1]. CEFM may be conducted via a cardiotocograph (CTG) either in wired, or wireless forms, internal scalp electrode (FSE) which involves a spiral electrode being inserted into the fetal scalp or breech [2, 3], or non-invasive fetal electrocardiogram (NIFECG), a wireless and beltless monitoring device which has more recently become available on the Australian market [4]. All forms of CEFM provide real-time information on the fetal heart rate pattern in conjunction with the measurement of uterine activity. Alternatively, intermittent auscultation involves listening to the fetal heart rate at specific intervals using a handheld Doppler device or Pinards’ stethoscope [5].

The aim of both CEFM and IA is to monitor the wellbeing of the fetus during labour and birth by providing fetal heart rate data to add to the overall clinical assessment of the woman or birthing person and their baby [1, 5]. Care involves excluding the suspicion of the presence of fetal hypoxia requiring further assessments and/or intervention to hasten the birth [2]. The choice of intrapartum fetal surveillance method depends on various factors, including the health status of the woman and her fetus, the stage of labour, the availability of monitoring equipment, and the preferences of the healthcare provider and the woman [3].

It is estimated that in Australia, more than half of the 300,000 women and birthing people who give birth each year are recommended to have CEFM in labour for indications such as induction or augmentation of labour, previous caesarean section, multiple pregnancy, use of epidural analgesia and/or delayed progress in labour [4]. Despite its widespread use, it is recognised that intrapartum fetal surveillance is not without limitations and challenges [6, 7]. Interpretation of fetal heart rate patterns can be subjective, and false-positive or false-negative results may occur, leading to unnecessary interventions or missed signs of fetal distress. According to the most recent Cochrane review, there is no evidence that continuous CTG monitoring improves maternal or neonatal outcomes, apart from a slight reduction in rates of neonatal seizures [1]. Furthermore, the review suggests that CTG may have deleterious effects, due to an association with increased caesarean section rates.

Literature about CTG monitoring often focuses on the safety of the fetus, as can be seen in key literature by authors including Chandraharan and Arulkumaran; Lamb and Heazell; Brocklehurst et al. [8–10]. This can overshadow the reality that the woman or birthing person, and the progress of labour, are impacted by the wearable or invasive medical device imposed upon them, often resulting in restriction of movement [11–14]. When labour is impeded, the potential for resulting intervention and surgical birth can result in negatively affecting the health of the neonate [15].

Since the introduction of wireless forms of continuous monitoring in 2003, qualitative literature describing women’s experiences of being monitored during labour and birth is limited [11, 14, 16, 17], and evidence of the experiences of women and birthing people in Australia remains scant [11]. To increase understanding of the experiences of women in the Australian maternity care system, we constructed a national survey with key questions about participants’ lived experiences of continuous fetal monitoring and/or intermittent auscultation. As far as we are aware, this is the first survey of the experiences of women and birthing people of intrapartum fetal monitoring conducted in Australia.

## Methods

This paper is derived from a larger mixed methods study, ‘Women’s Experiences of Monitoring Baby (The WOMB Study)’ exploring the experiences of women and birthing people of fetal monitoring during labour, and the information they had received about fetal monitoring during pregnancy. Data were collected via a cross-sectional survey that was developed in the platform ‘Qualtrics’ [18] and distributed online via social media sites. The survey was developed by the research team, based on their expertise in the topic area, and findings from prior research on fetal monitoring [4, 11–13], humanising birth [19], ethics and informed consent [20], and childbirth and parenting education [21, 22]. The survey was piloted by three women who had recently given birth, which informed subsequent revisions made prior to recruitment.

The survey was open to participants from 30th May to 30th June 2022. Participants were eligible if they had given birth in Australia in the past five years and had experienced any form of fetal monitoring during labour, either intermittent auscultation or continuous electronic fetal monitoring. The analysis of quantitative survey data will be published elsewhere.

Approval to conduct the study was granted by the University of Technology Sydney Human Research Ethics Committee (approval no. ETH21-6563) and ratified by the University of Notre Dame Human Research Ethics Committee (approval no. 2022-063S). A detailed research data management plan was approved by both ethics committees and all data collection and analysis complied with the requirements for sourcing the data. Information about the study was provided to participants to read before they commenced the survey. Informed written consent was implied by completion of the survey, as advised in the participant information and as approved by the ethics committee.

For this paper, we extracted specific data on the perceived benefits and disadvantages of the monitoring that the participants received, including their answers to the following questions:

- What do you think are the benefits of being monitored?
- What do you think are the downsides of being monitored?
- If given the choice, would you choose the same form of monitoring?
- How would you prefer to be monitored?
- Now that you’ve had your baby, what do you wish you had been told about monitoring during labour?
- Do you have any other comments you want to share about your experience of monitoring during labour and birth?

The qualitative and inductive content analysis approach of Elo and Kyngas [23] was used to synthesise the data from the open-ended survey responses. According to Elo and Kyngas [23], their method can be applied qualitatively or quantitatively and in the former, used either deductively or inductively [23]. An inductive approach was taken as we aimed to derive concepts from the data, rather than deductively testing an extant theory or set of assumptions. Although quantitative forms of content analysis often include percentages or counts of codes and concepts, we felt it most appropriate to use a qualitative and inductive approach. Hence, no percentages or counting of data are included in our findings, as the analysis looked for relationships, patterns, and concepts in the data, rather than frequencies. We followed the three-stage process of content analysis espoused by Elo and Kyngas [23] that is, *preparation*, *organising* and *reporting*.

The *preparation* phase involved reviewing all survey questions with open-ended qualitative responses and allocating them to each researcher for coding and analysis, according to their area of expertise [23]. Authors, DF, RC and EN individually conducted open coding of the data pertaining to the questions described above which focused on participants’ intrapartum experiences.

The *organising* phase followed, in which conceptual categories were formulated [23]. To commence this process DF, RC and EN met for a full day workshop where codes were shared, reviewed, and discussed to make sense of the data. We then mapped codes into categories on a whiteboard, comparing our open codes and sharing interpretations, moving codes into different categories eventually agreeing on a conceptual framework. We continued to meet regularly by Zoom over the following six months, in an iterative process to confirm final categories and sub-categories. The categories and coded data were presented and discussed with the rest of the research team (co-authors KL, VS and KS). After deliberation on the various conventions of Content Analysis, we chose to structure the findings into categories and sub-categories, to align with the Elo and Kyngas [23] methodology which espoused that the term ‘concept’ is used when theory development is the goal of the analysis, or ‘category’ when theory is not anticipated [24, 25]. We chose ‘category’ accordingly, and the sections of each category ‘sub-categories’.

*Reporting* involved writing up the findings collaboratively and supporting our analysis with exemplar quotes. Categories were refined and consolidated [23]. Transcribed quotes from respondents’ survey responses were used to illustrate the categories.

### Reflexivity statement

Of the six authors, four are midwives and two are maternal health researchers working with pregnant and birthing women as allied health practitioners. The team has previously conducted maternal health research, with quantitative and qualitative research expertise in humanising birth, fetal monitoring, childbirth and parenting education, epidemiology, and exploring women’s experiences. We all align with the midwifery philosophy espoused by the International Confederation of Midwives (ICM) [26] and believe in the importance of supporting birth physiology, using a critical research lens to question the status quo of the maternity care system in Australia. To ensure rigour, we continued returning to the data to ensure we were offering a transparent interpretation. We also checked each other’s thinking via robust discussion, providing alternative viewpoints to ensure that our personal philosophy did not dominate. We actively sought discontinuities in the data that challenged the categories we were constructing.

### Inclusivity statement

We respect gender diversity and acknowledge that not all people who are pregnant or giving birth identify as women. In this paper, the word ‘woman’ or the phrase ‘women and birthing people’ may be used interchangeably. Both are intended to be inclusive of birthing people who may not identify as women.

## Results

Valid responses to the survey were received from 861 participants. The average age of participants was 33 years, slightly higher than the most recently available Australian national average of 31.1 years [27], however, falling within the 30–34 year age range which accounted for the largest proportionate age range (38%) of all births in 2021. Responses were received from all states and territories of Australia, half from New South Wales and Victoria. More than 85% of respondents were born in Australia, higher than the national rate of 65.6% [27], which may be accounted for by the self-selection nature of the social media recruitment method used. Respondents identifying as Aboriginal or Torres Strait Islanders totalled 3.3%, lower than the proportion of First Nations women giving birth in Australia in 2021, reported as 5% [27]. Most participants were having their first baby (65%) and had completed tertiary level education (72%). In answer to the question, *What kind of monitoring did you have in the birth you are describing now*? 798 participants responded. Table 1 details the type of monitoring experienced by women in the survey, and where there was more than one type of monitoring (excluding admission trace), this was described as ‘multiple’ monitoring.

**Table 1:** Type of monitoring experienced by survey participants.

Monitoring Type		
Handheld		130 (16.3%)
Wireless		163 (20.4%)
Wired		281 (35.2%)
Fetal scalp electrode		39 (4.9%)
Non-invasive fetal electrocardiography		3 (0.4%)
Multiple (>1 form of monitoring)		182 (22.8%)
	**TOTAL**	**798 (100%)**

Two categories were constructed from the analysis of the data, *Tending to the machine* and *Impressions of the machine*. Each of these categories contained three sub-categories. *Tending to the machine* consisted of the sub-categories, *Midwifing the monitor*, *Over-reliance on an unreliable machine* and *Bringing the baby into the room*: *a double-edged sword*. The second category, *Impressions of the machine*, consisted of the sub-categories, *Feeling uncomfortable and restricted*, *Taking me out of the zone*, and *Wishing I knew it was a choice*.

### Category 1—Tending to the machine

The first category of findings, *Tending to the machine*, presents women’s perceptions of the way in which midwives and doctors interacted with the fetal monitoring technology. The category comprises three sub-categories, *Midwifing the monitor*, *Over reliance on an unreliable machine* and *Bringing the baby into the room*: *a double-edged sword*. Many respondents described how the focus of the room was on the monitoring equipment and data output, rather than their individual care. This left them feeling that staff were *midwifing the monitor*. They expressed concerns about an *over reliance* on the monitor as part of clinical decision making and found this especially frustrating when the data from the monitor conflicted with what their body was telling them. The audio and visual data accompanying the monitoring technology served the purpose of *bringing the baby into the room* which was reassuring if the baby was well but created more anxiety if there was some uncertainty, thus creating a *double-edged sword*.

#### Sub-category 1—Midwifing the monitor

Many participants who were monitored with a CTG reported that their midwife spent a lot of time interacting with the monitor that displayed the fetal heart rate data. They noticed that the midwife’s attention was directed towards the machine first, and the woman second, saying, for example: ‘*Everyone focuses on the monitor and not much else’ (participant 538)*, resulting in situations where it is perceived that the ‘*Machine becomes more important than the woman’ (participant 347)*.

Some participants noticed that midwives and obstetricians came into their birth space just to look at the machine, and then left without engaging with them, saying for example, *‘The doctors were focused solely on the CTG trace’ (participant 211)*. This led women to say that they felt invisible: ‘ *…people watched [the] monitor instead of me*, *[with] no trust in my body or baby’ (participant 56)*. They wished they had known that having continuous monitoring would mean there was a constant flow of ‘*people always in the room’ (participant 38)*.

There was a sense that for midwives and obstetricians, observing data from the CTG machine took precedence over noticing the woman or birthing person’s behaviour or what they were communicating. Women felt clinicians were listening to the machine over their body, saying that: ‘*It makes the professionals focus too much on numbers and not the woman’s individual experience’ (participant 157)*, and that they were, *‘paying more attention to the monitor than the woman—the woman is in labour*, *believe the woman’ (participant 230)*. They felt this negatively impacted the support they received because: *‘Midwives become totally focused on trying to get good monitoring rather than on providing good physical and emotional support’ (participant 197)*.

These behavioural signals suggested to participants that their experience was less valid than the data produced by the machine, which took priority over their experiential knowledge and directed the clinical decision making:

*I found it reassuring but also very frustrating because the monitoring really drove the actions of the midwife and OB and heightened risk in their eyes, and ultimately created stress for me that inhibited my capacity to focus on the birth (participant 833).*

*Some staff (mainly OB, not the midwives) seemed obsessed with the trace and the numbers and would forget to include me, the person actually giving birth, in the conversation about how my labour was progressing and how my baby was doing. This was especially frustrating when I specifically asked staff not to whisper about numbers, as it made me more anxious (participant 608).*

In addition to the data from the fetal monitor being prioritised, some women felt that maintaining a position in which to receive a clear fetal monitoring trace was more important to midwives than their bodily experience of labour. Participants reported being asked by the midwife to move, change positions, or keep still, to facilitate positioning of the CTG transducers ‘*… I had to keep so still to keep it in the right spot*, *it made the pain worse’ (participant 634)*. They felt their freedom to move and make choices according to their comfort was sacrificed, for the sake of maintaining continuous fetal heart rate data: ‘*Can’t move around*, *midwives get annoyed if you move and the monitor slips*, *feels really uncomfortable…[it] made it really hard to focus on mindfulness/breathing’ (participant 538)*.

The sounds of the alarms that emanated from the machine also provoked anxiety, as this woman described:

*My monitor continuously went off because it kept slipping, sending off alarms which made me panic about the well-being of my baby. The midwives didn’t immediately respond to the alarms, which made me wonder why they used them. When I asked why it kept going off, they told me not to worry. It was a bit stressful (participant 413).*

Whilst women accepted the use of fetal heart rate monitoring, they felt disappointed that this took priority over, and in some cases discounted, their bodily needs and experience of labour.

#### Sub-category 2—Over reliance on an unreliable machine

Many participants expressed concern about the accuracy of fetal heart rate data emerging from the CTG, and felt it was an unreliable source of information. Some even referred to wider implications for maternity care:

*The [CTG] affects your ability to labour…it’s not evidence based, can be wrong… the people using it have now lost the skills to assess birth without it… the research shows it does not actually prevent death or CP [cerebral palsy] (participant 123).*

There was an awareness that the data obtained from fetal heart rate monitoring carried significant weight in the eyes of hospital clinicians and was used to guide clinical decision making, particularly in regard to interventions:

*The monitoring determines what intervention can and should be used according to policy…it can fall off and provide an inaccurate reading and the medical professionals still base decisions on it when it drops out (participant 57).*

Women were especially disconcerted when clinicians relied on the interpretation of the machine data when it differed from their bodily experience: *‘I didn’t like that the midwife doubted when I was contracting*, *based on the machine*, *and was told I wasn’t*, *only for another midwife to say I indeed was and that she could feel it on my tummy’ (participant 909)*. They identified that there were flaws in the data, such as false negatives or false positives:

*My baby was deprived of oxygen for a significant period of time during labour DESPITE continuous CTG monitoring indicating nothing out of the ordinary. At no point in time (despite the monitoring) was I given any indication that the baby was in distress…baby was born with an APGAR of 0 and required 4 minutes of CPR…I thought AVOIDING these things was the reason WHY hospital use continuous CTG monitoring! [capitalisation in original quote] (participant 41)*.

There was a perception that the use of CTG technology was mainly a way for the hospital to protect itself legally, saying, for example, *‘It’s not to save my baby*, *it’s to save the hospital’s backside’ (participant 56)*. This had implications for the choices women could make about their labour: *‘Hospitals require a good CTG to be able to use the bath and if they can’t get one*, *they won’t let you in’ (participant 74)*.

Some of the negative experiences of monitoring were as a result of women feeling that the technology was unreliable, which became starker when the data disagreed with their own physical experience.

#### Sub-category 3—Bringing the baby into the room: A double-edged sword

The presence of fetal monitoring had the effect of bringing the baby into the room because of the way the aural and visual data from the baby’s heart rate attracted everyone’s attention. Women described the different ways in which they felt the monitoring kept their baby safe, as one said: *‘It was good to know my baby was okay the whole time’ (participant 387)*. For some, the sound or the visual data reassured them that the baby was doing well, *‘I was very anxious*, *going into labour*. *The intermittent monitoring gave me reassurance’ (participant 191)*. For others it was more about knowing that the monitor would alert them if the baby became distressed. Some women regarded it as important data for health professionals to use as part of their clinical decision making. Despite the challenges of wearing a CTG, some women enjoyed hearing their baby’s heartbeat via the monitoring device, saying for example: *‘To be honest I quite enjoyed listening to my baby even with the CFM that I didn’t particularly want’ (participant 113)*. One woman found it made her feel *‘reassured and calmer*, *hearing my baby’s heartbeat’ (participant 576)*.

Knowing that the monitoring would indicate if and when their baby was distressed was valued: *‘My baby ended up having some indications of distress*, *so I am grateful that [it] was discovered through the monitoring’ (participant 493)*. It meant that intervention could occur in a timely manner, keeping their baby safe: *‘It allowed for my midwife to recognise early and quickly that my baby was in fetal distress and allow for appropriate management’ (participant 635)*. It also helped alleviate concerns for a woman who said she had *‘a fear-based mindset and was very afraid for baby*, *so felt some comfort from thinking monitoring might help identify any problems’ (participant 321)*. This form of reassurance was particularly pertinent for women with experiences of fetal distress or adverse events during a previous birth, for example: *‘As there were concerns of stillbirth in my first labour*, *it was reassuring to hear/see a heartbeat’ (participant 617)* and *‘As my first birth was traumatic…it was reassuring to hear the heartbeat during my 2nd birth’ (participant 685)*.

Other participants valued the visual data, saying, *‘I like being able to see how baby is going during labour’ (participant 149)* and *‘It helped me to see that my labour was progressing’ (participant 384)*, although the converse was also true. The appearance of non-reassuring patterns could increase their anxiety, saying: *‘[I felt] reassured that I could see her heart [rate patterns] but [it was] also very anxiety provoking when her heart rate was dropping intermittently*, *and I was alone in the room feeling very scared’ (participant 267)*.

Receiving ongoing communication about whether the fetal heart rate was reassuring was important to many women. Lack of communication about what they were hearing or seeing was a determinant of anxiety and uncertainty: *‘Communication could be improved to take stress away’ (participant 508) and CTG monitoring was specifically ‘a source of anxiety…if we aren’t properly informed on how it works and what is normal/abnormal’ (participant 697)*. Others commented on the importance, for them, that the staff could assess how their baby was coping, via the monitor: *‘It was good to know that the medical professionals were keeping a close eye on my baby’ (participant 696)* and *‘Bub’s heart rate stayed consistent throughout my labour and midwives communicated that they were really happy with that’ (participant 554)*. Yet another reason for reassurance was that women could see that monitoring allayed doubt/fear in their health care providers: *‘I guess because it allowed my health care team [to] know that she was okay’ (participant 479)*. A woman who transferred to hospital during labour after a planned home birth said, *‘the CTG reassured the doctors that*, *even though I had laboured at home for so long*, *my baby and uterine scar were fine’ (participant 530)*.

Hearing alarms emanating from the monitor was disconcerting: *‘I was panicking when the beeps and noises would change*, *even though [my] baby was ok’ (participant 843)*. The sounds heightened their anxiety about their baby, impacting how they were coping with labour: *‘The application of [the] monitor…increases anxiety*. *I had to ask for the sound to be switched off because it was so loud right next to me and so fast’ (participant 130)*. Hearing or seeing the data could therefore create a double-edged sword, in that it could be both reassuring and anxiety-provoking at the same time: *‘It was reassuring but also made me anxious looking at a monitor for any slight change in readings’ (participant 447)* and, *‘It’s reassuring to know that their little heartbeat is still going strong*, *although it was very worrying when it was lost or low or they were unable to find it’ (participant 537)*. It was not only women undergoing continuous electronic fetal monitoring who experienced anxiety. One woman felt anxious every time intermittent monitoring was imminent, saying: *‘I found it anxiety inducing every time I saw the Midwife with the doppler in her hand preparing to listen*, *I’d worry that she would find something abnormal and my lovely*, *natural*, *undisturbed birth might end there’ (participant 78)*.

The use of synthetic oxytocin during labour poses additional risks that women were aware of, saying, *‘I understood that with syntocinon it was important to have constant monitoring of baby’s wellbeing’ (participant 197)*. For them, monitoring was an important way to observe whether the dose was being tolerated by their baby: *‘It was good to be able to see how well my baby was tolerating the syntocinon rate increases and titrate them as tolerated’ (participant 608)*.

It was not only the women that were affected by the fetal monitoring, some participants noticed that their partner was also distracted by the machine. They felt that the machine took their partner’s attention away from their needs during labour:

*Birth partners may get fixated on the numbers and not be paying attention to the birthing person and the headspace of the birthing person if they are worried about the monitors, it may not allow them to get in the right headspace for birth (participant 81)*.

This first category, *Tending to the machine*, described the impact that fetal monitoring had upon the care and support that women received. The next category focuses more closely on impact upon the women’s experiences of labour.

### Category 2 Impressions of the machine

Category 2, *Impressions of the machine*, explores the direct impact of fetal monitoring upon women, and comprises three sub-categories, *Feeling uncomfortable and restricted*, *Taking me out of the zone*, and *Wishing I knew it was a choice*. All forms of fetal monitoring presented some challenges to women’s comfort beyond the discomforts of being in labour, leaving them *feeling uncomfortable and restricted*. Their ability to stay focused on their labour, which they referred to as ‘being in the zone’, was compromised by interruptions that were driven by the need to gather technological data by clinicians, resulting in women being taken *out of the zone*. Women reflected that during pregnancy and early labour they had received limited information about the potential impact of monitoring on their labour, providing little opportunity to give their informed consent. This left them *wishing I knew it was a choice*.

#### Sub-category 1: Feeling uncomfortable and restricted

Participants wrote about a range of impacts that fetal monitoring had on their comfort in labour, from the discomfort of tight elastic belts to a lack of mobility and bodily autonomy. They typically described the belts and transducers of CTG monitors as being very uncomfortable: *‘[I was] very uncomfortable*, *straps very tight’ (participant 155)*. The need for constant readjustment of the CTG transducers was a common cause of discomfort: ‘*staff pushing hard on the monitors trying to pick up a trace’ (participant 155)* and for some women felt invasive *‘… it is not comfortable and can be quite intrusive with all the extra hands touching you trying to keep it working properly’ (participant 156)*.

Women described how the monitoring, ‘*restricted my movement…the midwife had to hold it to my abdomen during contractions when I was transitioning and I found that very uncomfortable’ (participant 556)*. This impacted their capacity to move into their position of choice, ‘*They kept losing bubs heart rate and it made it difficult to move into comfortable positions’ (participant 360)*.

The experience of discomfort extended to those who were monitored with the wireless CTG (telemetry): ‘*The wired one makes it so you can’t move around properly*, *and the wireless one kept moving down and almost falling [off]’ (participant 491)* which meant that, *‘not all labouring positions are possible*, *even with wireless monitoring’ (participant 68)*. When wireless CTG (telemetry) was utilised, women reported the transducers needed to be frequently repositioned: ‘*Couldn’t move as freely even though it was wireless*, *it would slip easily*, *machines would alarm*, *and I’d have to wait for it to be repositioned by a midwife’ (participant 325)*. The fetal scalp electrode was also found to restrict movement, as this woman described, *‘when they were monitoring directly from bubs head*, *I couldn’t really move at all’ (participant 359)*.

Women’s intentions for using mobility to cope with the pain of labour could not be realised when they were being continuously monitored: ‘*I was unable to move around and that had been my plan before labour began’ (participant 593)*. There was a disconnect between the information shared with them during pregnancy and the reality in the birth space and they wished they had known that they, *‘wouldn’t be able to move around freely’ (participant 670)*. Although women were given information in pregnancy about active birth strategies that would be available to them in labour, these options were taken away when they were in labour, *‘We were told about [the] birth ball*, *the bath*, *movement*, *breathing… hey all of that stuff is useless because you’ll be strapped to the bed most of the time’ (participant 441)*. This left women ‘*being stuck on the bed’ (participant 94)* and unable to perform basic bodily functions such as accessing the toilet, ‘*I couldn’t really leave the bed*, *I would have to ask to be unattached to go to the toilet’ (participant 391)*.

Being unable to move or adopt positions freely in labour led women to feel a decreased sense of bodily autonomy. One woman described her frustration with the fetal monitoring she had experienced in several labours:

*Constantly losing the heartbeat when you move can make you anxious that there’s a problem with the baby. Also [monitoring is] a disincentive to move around. I gave up on bouncing on a Swiss ball because it wasn’t possible with the monitoring. For one birth I opted for a [fetal scalp electrode] monitor to be placed on the baby’s head because the wireless monitor needed constant readjustment, which meant I couldn’t use a bath for pain relief. For my third birth I just lay in bed using the gas a lot because moving around was so frustrating (participant 583)*.

Some women reported that their need to use pharmacological methods of pain management was directly related to their movement being restricted by continuous monitoring, ‘*I couldn’t move to manage my pain so ended up needing gas earlier than I hoped’ (participant 744)* and, ‘*Being bed bound*, *uncomfortable and stressed during labour [leads] to other interventions such as epidurals to manage pain that you can’t manage in other ways’ (participant 870)*.

Lack of access to water immersion, often due to hospital policies banning it in the presence of continuous monitoring, was also a concern: ‘*[I wish I had known] that I wouldn’t be allowed or able to use the birthing pool’ (participant 159)*. Continuous monitoring meant they lost access to water immersion, which was disappointing, for example: *‘I had wanted to use water as pain relief but was unable to*, *due to the monitoring they wanted me to have’ (participant 55)*, ‘*It felt like there was no choice given to me—and it prohibited me from having a water birth’ (participant 15)*, and *‘I was made to get out of the bath to continue to give birth’ (participant 728)*.

Lack of access to water immersion, often due to hospital policies banning it in the presence of continuous monitoring, was disappointing for many women, as the following quotes illustrate:

*[I wish I had known] that I wouldn’t be allowed or able to use the birthing pool (participant 159)*.

*I had wanted to use water as pain relief but was unable to, due to the monitoring they wanted me to have (participant 55)*.

*It felt like there was no choice given to me—and it prohibited me from having a water birth (participant 15)*.

*I was made to get out of the bath to continue to give birth (participant 728)*.

The use of continuous fetal monitoring devices had a significant impact on women’s experiences of labour. Many felt unprepared and misinformed about the way monitoring would reduce their bodily autonomy in labour and potentially alter their plans for birth.

#### Sub-category 2: Taking me out of the zone

Many women described the intense focus they experienced in established labour as ‘the zone’. As well as the physical impacts of continuous monitoring, participants wished they had known how much continuous monitoring would affect their focus, as they experienced: *‘Interruption of mental state*, *lack of flow to get in the zone to labour/ birth…’ (participant 892)*, also describing the effects of this disruption on the process of labour:

[I wish I had known] that it will pull focus from where my mind should be during labour. That it makes people come into your birthing space far too often to fix it up. That it limits movement even if wireless because it moves around so much…which can lead to interventions and caesareans (participant 157).

Other women identified how monitoring ‘*brings attention out of the labour zone’*, saying, for example, that they were, *‘unable to move*, *relax*, *get comfortable or get in the zone’ (participant 233)*.

An important finding is the impact that restricted mobility in labour may have for women and birthing people who have previously experienced sexual abuse. Two women described how being continuously monitored during labour resulted in a re-traumatisation of previous sexual abuse due to feeling ‘tied to the bed’ and unable to move or ‘escape’:

I felt tied to the bed like I couldn’t get off it, I felt very restricted in choices of labour positions, it contributed to my re-traumatisation from previous sexual assault (participant 766).

I was tied to a bed and unable to move/escape, it contributed significantly to being re-traumatised from previous sexual abuse, I believe it hindered my ability to give birth unassisted because I was limited in my choice of labour positions (participant 883).

It is evident that feeling uncomfortable and restricted by fetal monitoring devices negatively impacted women’s enjoyment of labour and their capacity to utilise positions that helped them cope with the pain of labour. For some women, such as those who had previous experiences of sexual abuse, the sensation of being restricted had significant adverse psychological impacts.

#### Sub-category 3: Wishing I knew it was a choice

A major concern for participants was that the option of CTG monitoring was not presented to them as a choice, and that all options were not explained:

*Just [would have liked knowing] all options available and when one may be required so I could make a more informed decision instead of feeling pressured to have [fetal scalp] electrode monitoring (participant 71)*.

Other women described feeling disrespected when their informed choice to decline continuous monitoring was overridden, wishing ‘*that my request to decline had been respected’ (participant 103)*, with one woman reporting it as traumatising:

*I wish my midwife had respected me when I said I didn’t want CTG monitoring. It started the cascade of interventions that I didn’t need or want and left me with severe PTSD (participant 62)*.

Even when they knew their options, women stated that, *‘standing up for what I wanted was difficult’ (participant 65)*. Women did not feel prepared for how significantly the form of fetal monitoring being used would take them *out of the zone* during labour.

Participants described being unaware of their options, or that they had a choice about whether or not to be continuously monitored, because it was often presented as a necessary fact rather than an intervention for which they needed to give informed consent: ‘*[I wish I had known] that it wasn’t compulsory or that there were other methods available (participant 107)*.*’* They wanted to be given information and choice about forms of monitoring: ‘*[I wish I had known] that you had a choice [and the] key reasons for it and expectations on how it will work upfront (participant 96)*.*’* Many women questioned whether continuous monitoring was necessary during their labour: ‘*[I wish I had known] whether it was truly important to be monitored at that moment in time’ (participant 99)* and wanted to be better informed about their right to decline care: ‘*[I wish I had known] that I could have refused if I chose (participant 54)*.*’* Some discovered their right to decline experientially: ‘*At one point I pulled all the cables and straps off which apparently alerts the midwives*, *but no one came to reattach them*. *After that I figured they really weren’t needed (participant 100)*.*’*

In this category, we have identified how the ‘impressions of the machine’ can be experienced bodily, and as restricting options for pain management. This has a negative influence on labour, not only due to provocation of anxiety and discomfort but also disrupting the hormonal labour state described by participants as the ‘zone’. Not only is the use of these technologies disruptive, but it is often presented as mandatory, rather than as a health care intervention that requires informed consent before use.

## Discussion

The findings of this study provide a comprehensive dataset on Australian women’s experiences of fetal monitoring in labour. The two categories constructed indicate the influence of fetal monitoring technologies on women and birthing people during their labour and birth experiences. The first category, *Tending to the machine*, outlines participants’ perceptions of the way clinicians interact with fetal monitoring technologies, indicating their sense that the technology and its associated data was privileged over their lived human experience. Although the survey was open to women who had experienced all forms of fetal monitoring, the majority of responses referred to experiences of CEFM. The presence of audible and visible continuous fetal heart rate data in the birth room was considered a ‘double-edged sword’, increasing anxiety for some who were not comfortable with the continual changes in fetal heart sounds and allaying the fears of others who felt reassured by the continuous trace.

The second category, *Impressions of the machine*, described the direct impact of fetal monitoring devices upon the labour and birth experiences of women and birthing people. These impressions included significant discomfort due to tight elastic belts of CTGs, restrictions to freedom of movement and the resultant inability to employ planned strategies for managing the pain of labour. This category also outlined the lack of information provided during pregnancy relating to their options for fetal monitoring in labour and a lack of informed consent which left women ‘Wishing they knew it was a choice’.

Using a care ethics lens [20, 28–31] to interpret these findings identifies an endemic lack of concern in maternity settings for genuine ethical care. Although technology can be useful in contributing to an overall clinical picture, it is not a replacement for attentive, human caring. Dutch philosopher Peter Paul Verbeek’s post phenomenologist theory of technological mediation [32] addresses the ways in which humans experience technology in the world, and the ways in which human experience is shaped by technologies. Verbeek [32] proposes that, as humans, we need to mediate our interactions with technology, rather than being locked into historical perspectives of seeing technology as either instrumentalist (under our control) or determinist (in control of us). By rejecting the notion of technology use in the birth space altogether, we minimise the opportunity to consider its impact in a nuanced manner.

When entering a hospital labour room, fetal monitoring technologies promote a notion of fetal personhood, by inviting us to listen to the fetal heart and watch its physiological patterns on a screen. In 2008, Verbeek wrote an analysis of the impact of routine antenatal ultrasound upon our understanding of fetal personhood, and parental-fetal relations [33]. His approach was reminiscent of the historical perspective presented in 1993 by Duden [34]. For example, the recognition of pregnancy was once a profoundly personal bodily sensation for a woman experiencing ‘quickening’, technology has brought about the anticipation of external, and often biomedical, confirmation of pregnancy [34]. Routine pregnancy ultrasound examinations may provide this confirmation prior to the woman’s own bodily experiences, whilst establishing the status of the fetus as a potentially independent being, separate from its mother [34]. We can observe fetal movements, make anatomical measurements, and share photographs of an unborn baby, months before they are born. Duden posits that technological transformations have strengthened the independent legal status of the fetus and the sense that pregnant women’s bodies need controlling, as we operate within what Duden refers to as ‘the age of the public fetus’ [34] (p.55).

We propose that the perspectives of care ethics [20, 28, 30, 31] and technological mediation [32] can equally be applied to fetal monitoring in the context of caring for women and birthing people during labour. By this, we mean that midwives and other health professionals should not simply comply with the demands of the fetal monitoring technology by *midwifing the monitor* instead of the *person*, which arguably increases the risk of physical and emotional harm. Instead, midwives and other health care professionals can *mediate* the impact of technologies upon the experiences of women and birthing people, while sustaining an ethics of care which keeps the individual at the centre by focusing on authentic engagement, attentiveness, and relationality [30, 31].

The first category of results, *‘Tending to the machine’*, shows the propensity of continuous CTG monitoring in labour to shift the focus of care from the birthing woman or person to the machine. The presence of the machine in the room led to the experience of the technology having more importance than–or dominating—the lived human experience. The belief that CTG monitoring presents the safest form of monitoring an unborn baby, and that it can prevent an adverse fetal event, is an example of technorationalism. The concept of technorationalism includes the idea that ‘complex’ (e.g., technological) solutions are more suitable than simple (e.g., human care) ones, and views all technology as ‘progress’, whether or not it actually provides any benefit [19].

Preoccupation with the machine necessarily entails reduced interaction with the birthing woman, hindering midwives’ ability to be authentically ‘with woman’ [35]. Midwives have reported that CTG transducers require constant ‘fiddling’ to maintain connection and expressed that new technologies are urgently needed that better account for the needs of midwives and women in the birth room [12]. In our study, women sensed that their midwife was inconvenienced by their instinctive need to move and change positions in labour, sending a message that the fetal heart rate was the central focus of the midwife’s work, superseding the woman’s comfort and bodily autonomy, thereby reducing her choices.

In an institutional ethnography of the effects of a central monitoring system, where all CTG readings are available to all staff in a central location [36], midwives described being concerned by an emerging practice where, on seeing the reading, a clinician (doctor or senior midwife) would enter the labour room, having made interpretations and decisions, before consulting with the attending midwife or the woman herself. This provoked a sense of anxiety for midwives, and our findings align with this, from the perspective of birthing women and people. This shift in focus, from the woman–who is supposed to be at the centre of care–to the machine–was experienced as dehumanising. A resulting circumstance of the central monitoring system was the notion that clinical decision making was undertaken without a full clinical picture [36], and this was also shown in our subtheme, *over-reliance on an unreliable machine*. The women in our study described knowing that a non-reassuring trace was due to something happening in the room–an embodied and experiential knowledge—such as movement or loss of contact, but this information is not inherently embedded into the CTG data (though it can be added manually).

Perhaps more concerning is the second category, *Impressions of the machine*, which illustrates the way that fetal monitoring was experienced by women as a certainty. According to the four principles of biomedical ethics [37, 38], treatments and interventions in medical settings can only be given with full and informed consent. The context of pregnancy and birth carries no legal exemption [39]. Recent research identifies that in the maternity sector in particular, this does not always occur [29, 30, 40]. Knowledge and policy, skewed towards medicalised processes, shapes the way that choices are presented to pregnant and birthing women and people [41]. In addition, women have described feeling coerced and bullied in their interactions with perinatal medical professionals [42, 43]. Presenting any clinical intervention as a given necessity, or as routine rather than a choice, as described in the second category, *Impressions of the machine*, does not conform to current ethical standards. The idea that CTG monitoring can save a baby’s life is misleading, based on current evidence. And yet, this was an overwhelmingly common experience expressed by the women in this survey. Often, women who should have been eligible for IA were nevertheless continuously monitored with CTG. Women who declined monitoring, or particular forms of monitoring, had their wishes overridden [42, 43], which contravenes the ethical principle of respect for autonomy [37, 38], and is experienced as dehumanising and potentially as causing trauma.

Hospitals used information gathered from CTG as a way of gatekeeping particular practices. For example, some women described having to have a CTG trace before getting into the bath, even though a reassuring admission trace is not predictive of birth outcomes [44]. Interestingly, since 2003, continuous monitoring has been possible during water immersion with waterproof CTG (telemetry), but hospital policies are often prohibitive, or hospitals do not invest in the appropriate equipment for this to occur [13].

The strength of this study is that there is little research published on women’s experiences of intrapartum fetal monitoring in Australia, and to our knowledge, no survey data that has attracted such a large response such as the 800+ responses received. The rich data provided detailed hospital experiences from across all states and territories of Australia. Some respondents who were commenting about their experience of monitoring in their own labour and birth identified themselves as midwives, providing an interesting perspective from both sides of the lived experience.

Limitations include the nature of recruiting via social media, and we acknowledge there is a higher chance that the participants are self-selecting and may be more likely to have a negative interest in the topic. However, the rich data provided detailed hospital experiences from across all states and territories of Australia. It was also clear from scrutinising the data that some respondents who were commenting about their experience of monitoring in their own labour and birth, also identified themselves as midwives, providing an interesting perspective from both sides of the lived experience.

The principle of informed consent protects women and birthing people’s rights to voluntary consent or refusal of any medical treatment, procedure, or intervention based on the provision of sufficient, evidence-based information to make a decision that reflects self-determination, autonomy, and control [39, 45]. Our findings indicate that many women did not feel they had adequate information to make an informed decision relating to the method of fetal monitoring used throughout their labour. As such we strongly recommend that clinicians ensure they discuss fetal monitoring with women in the antenatal period, providing adequate verbal and written information outlining risks, benefits and alternatives. In addition, women need an opportunity to discuss this at the commencement of labour, with clinicians ensuring they gain continual consent rather than presuming consent once a form of fetal monitoring has been employed.

Other implications for practice include encouraging midwives to become more proactive in mediating the impact of fetal monitoring. Midwives need to take responsibility for the way in which they care for women and birthing people in the presence of technology, and for considering how the chosen form of monitoring technology may impact their experiences and ability to labour. For example, freedom of movement is an important aspect of supporting the physiology of labour and birth and managing pain. If freedom of movement is being prioritised, the restriction created by the use of wired CTG is clearly going to be inappropriate. Other options would need to be discussed. Keeping the birthing woman in mind and prioritising individual needs are key practices needed to provide high quality woman-centred (inclusive of all birthing people) intrapartum care. Future clinical guidelines and professional position statements need to address this issue, and many currently do not.

Future research implications include exploring ways to promote informed decision-making during pregnancy around options for intrapartum fetal monitoring, so that women and people have bodily self-determination as active participants in the assessment of their labour and the well-being of their baby. Additionally, a few women who have experienced sexual abuse self-identified in this survey that restriction of movement in labour and birth was re-traumatising. More in depth research is needed to explore this sensitively, to understand the full impact of restriction of movement for these women. The findings showed that clinicians’ shifting their attention away from the woman, who should be at the centre of her care, towards the machine, may contribute to suboptimal clinical decision-making and reduced options for women; something that needs to be explored in future research.

## Conclusion

The findings of this research highlight serious issues which may be integral to current problems in maternity services, such as reliance on technology over human interaction, and overriding the right to bodily self-determination. These findings are concerning not only because they highlight how monitoring technologies can disturb the neurohormonal processes of labour, but, more alarmingly, the way such technologies are experienced as mandatory, leading to enforced physical restriction and therefore a denial of human rights. Midwives need to do better to uphold this right and to ensure that individualised information is given about types of intrapartum monitoring to ensure informed consent. This is important not only in the humanising of care, critical evaluation and mediation of technology is a key element in humanising birth. A crucial aspect of this is for midwives to take responsibility to mediate the impact of fetal surveillance on women and birthing people and to incorporate professional guidance on how to best do this. When using any technology, it is important for midwives to direct attention to the care, support and information needs of the birthing person, and use the information gained to support these needs.

## Supporting information

S1 File (PDF)
