# Supplementary material for: Tending to the machine: The impact of intrapartum fetal surveillance on women in Australia
Source: PLoS One. 2024 May 9;19(5):e0303072. doi: 10.1371/journal.pone.0303072 (PMC11081371; doi:10.1371/journal.pone.0303072)
Supplement: S1 File — (PDF) [file pone.0303072.s001.pdf]

The WoMB Study: Women's Experiences of Monitoring Baby

The qualitative questions the survey asked participants to respond to were:

1. **What do you think are the benefits of being monitored?**
2. **What do you think are the downsides of being monitored?**
3. **If given the choice, would you choose the same form of monitoring?**
4. **Now that you've had your baby, what do you wish you had been told about monitoring during labour?**
5. **Do you have any other comments you want to share about your experience of monitoring during labour and birth?**

1. What do you think are the benefits of being monitored?

- Yes. It can be reassuring for mothers.
- Detect any abnormalities
- Reassurance baby is coping with contractions
- To be able to constantly keep track of baby's heartbeat
- Keeping track of the baby's heart rate
- Checking baby's condition - reassuring
- Some assuredness that baby is ok during the process
- Making sure baby is ok when induced
- Health professionals being able to identify changes in heart rate of baby.
- That the baby is okay especially for the first time
- For me hearing my baby's heartbeat
- Keeps birthing mother assured she is doing it well, health staff can support her decisions if they know baby is ok.
- Being able to identify any change in baby's heart rate
- For me, reassuring to know baby was ok during moments of doubt
- Early recognition of a compromised baby and therefore early intervention
- You know the baby is safe and okay
- Slight destress in bub towards the end of labour was noticed and gave me the drive to push her out
- If there is a suspicion that the baby is under stress
- Makes staff feel more comfortable when there are lots of birthing women
- Unsure - doctors and midwives determined baby was distressed/having trouble and intervened
- Confidence that baby was not distressed, able to see contractions with epidural
- Hearing babies heart beat can identify progressively deteriorating heart rate
- Detect whether the baby is in distress

- Reassured the midwife and my partner - I didn’t really think about it in labour and I could feel the baby moving anyway
- For me none. For the baby maybe some. Her heart rate started to decelerate during the final pushes as her shoulder got stuck. The obstetrician had to reach in, turn her around and pull her out. There were midwives involved in the move pushing down on my tummy.
- Knowing the baby is doing ok, reassurance
- To monitor baby if In distress
- Beneficial for the care provider
- Caregiver confidence
- Ensure safety and well-being of both mother and baby.
- None for the woman
- I felt assumed that my baby was coping with labour appropriately
- Make sure baby is safe (if done correctly and reviewed by midwives etc)
- In some situations, there may be benefits of providing reassurance that baby is ok
- Check that baby was coping well with labour and doing well
- Knowing straight away if my baby wasn't coping with the labour
- To make sure baby is not in distress and medical staff can act quickly if baby is
- I had preterm labour and two uterine scars - to use as part of an overall assessment for how baby was during labour
- Midwives can follow CTG on a screen outside of birth room which provides privacy for you in labour.
- You can see how baby is coping with labour
- Ensuring fetal wellbeing
- The staff feel they have a better understanding of what is going on with baby
- No, Doppler and watch women and listen to them. CTG hinders labour
- I now really don’t believe there are any - especially not the wire monitoring.
- Having information on baby's condition
- Reassurance that baby is ok.
- Baby stays safe
- There are none
- Knowing babe was well
- Reassurance
- Checking if baby is ok, but again, just trust the woman and watch her while in labour
- Knowing that my daughters heart rate was dropping and she was in fetal distress so we could have an emergency c-section
- Knowing that baby was doing ok
- For me it helped me avoid a LSCS as well as enabled me to rest in a long labour
- Knowing baby is not in distress
- Ensuring the baby is not distressed or dropping HR
- Keeping an eye on baby
- To see the recovery of bubs heart rate however this is not a lone indication of any issues
- To monitor for fetal distress for true emergencies
- Checking on the baby’s wellness

- Due to my weight, the midwife was unable to easily monitor my babies heart rate. So continuous monitoring was the best option particularly with being induced and having an epidural
- Knowing baby is not in distress
- Early detection of foetal compromise
- To check in on the baby
- It was nice to hear her heartbeat, and I suppose there was some medical reason to do so
- If baby does get distressed you can pick it up quickly and there are certain times I'm sure the monitoring saves a baby's life
- Prevent stillbirth
- Reassurance of baby's welfare?
- Wellness of baby
- Makes the hospital feel better
- I had a little bit more breathing space as I had consented to what doctors had been pushing for.
- Making sure the baby is okay
- Knowing that my baby's heartbeat was strong and happy
- No benefits for continuous monitoring...intermittent monitoring to double check baby is coping with labour
- Early detection of deterioration of my baby
- If something goes wrong you can catch it early. Things like eg.. lack of oxygen to bubs brain causing cerebral palsy would be a catastrophic outcome if it could be avoided by knowing somethings amiss.
- You can have real time knowledge about your baby's wellbeing. It also helped me be like yep that was a real pain I'm not imagining it
- When there are other risk factors such as meconium or prolonged labour doctors are happier for labour to continue when they can see baby is happy
- Peace of mind, more data on baby's movements
- Heart rate and distress for bub when mum had preeclampsia
- Reassurance and motivation. If something was wrong the care provider could know sooner.
- Checking on baby's welfare.
- If the mother is induced or using pain relief then ir makes sense to monitor baby
- It offered peace of mind however I wasn't concerned and found the intermittent monitoring with the Doppler to be sufficiently reassuring.
- Reassurance baby is ok
- If you have a medical condition then continuous monitoring can help prevent anything bad happening. Intermittent monitoring for Al other people allows midwives to understand if/know if baby is reacting bad to the labour.
- Very little as not enough evidence on interpretation.
- You know bub is okay
- Knowing my baby isn't distressed
- Ensure babies heart rate is stable
- I was told they had to track babies heart rate so that if things went wrong they had time to prepare for a caesarean

- Peace of mind (nursing background) ensuring baby is ok ensuring mum is ok
- Reassurance of fetal heart rate but don’t find CTG to be superior. In the context of IOL, I felt it was safer
- Understanding if baby could be in distress by their heart beat although this could be false
- Able to check no issues with baby heart beat during long labour
- Picking up an abnormal heart beat quickly
- Definitely fetal well-being
- Reassurance that baby was well and encouragement to keep going as I was.
- Peace of mind about baby’s wellbeing
- It is circumstantial. I guess it benefits to know what the babies HR is. But I don’t know if continuous is necessary.
- Might be able to indicate fetal distress
- Fetal wellbeing at that present time is assured
- Recognising fetal compromise
- I wasn’t sure there were benefits for me
- There are none
- Keeping an eye on how baby is travelling
- Health of baby, health of mum
- Maternal reassurance
- Fetal distress or abnormal events are traced
- Absolutely. It was able to detect my baby in distress
- Health professional able to provide earlier intervention to minimise birth risks and trauma to baby.
- I think monitoring has its place for at risk babies but there are more risks for monitoring in low risk pregnancies.
- Knowing the baby was safe
- Knowing baby is safe and well oxygenated, able to monitor contraction timing adequately with the toco.
- I pretty much did CTG to keep the Drs off my case. I was having a VBAC with a big baby, and they wanted to do RCS. I understand that CTG means they can see if baby is in distress, but I don’t think it benefitted me any more than a midwife listening intermittently
- Knowing baby is coping ok with labour
- They are great that showing you a baby is well
- Understanding how baby was coping with labour
- None
- Check on baby HR and how they are coping with labour.
- Ensures that baby is not distressed or compromised during labour.
- Few
- I can't think of any
- Reassurance that baby is okay
- Confirming baby was safe
- The health professionals can tick their boxes
- No!

- Small benefit in hearing babies heart rate to see how they are coping intermittently. No benefit to continue monitoring
- In case the baby is in distress they will know about it
- Reassurance that baby is doing well.
- Checking that baby isn't in distress
- Being able to ensure babies heart beat was stable especially when concerned about decreased feral movements
- To check baby is ok
- Due to reduced fetal movement was reassuring to know baby was okay.
- From my perspective no, I was not told what they were seeing on the monitor and it gave me no reassurance. The staff may have felt more comfortable with me having it on
- For the midwife and me and my husband to know all was okay with the baby's heartbeat and heartrate, being comfortable to continue to labour naturally knowing the baby was doing fine.
- I think there would be times when it would be helpful but not for every woman
- Good for high risk women
- To see if baby is in distress.
- Know what/how baby is doing
- Ensure fetal wellbeing
- In case bub is at risk then they know quickly and can intervene.
- Reassurance
- Checking how baby is coping during the labour
- There are none - u less you consider peace of mind for the midwife or OB to be of benefit
- None.
- Knowing that the baby is tracking well through labour
- Peace of mind if wanted
- For me, reassurance
- Knowing baby was ok during labour
- Check on welfare of baby
- There were no benefits for me, but some people find it reassuring, and it makes life easier for hospital staff.
- Knowing babies ok
- It’s vital to be aware of the health of bub if there are risks so monitoring isn’t a question for me.
- I have yet to see that they are reducing intervention rates
- Identifying if baby is in trouble to enable intervention. Reassurance for me. Ability to keep pushing for longer as baby doing fine.
- If mum or bub is actually high risk then I understand the need for monitoring closely, and the potential need for early interpretation and decision making around intervention. For otherwise low risk births, there is potentially more harm than benefit.
- Knowing the baby is fine
- To help ensure baby is happy inside and coping with labour
- Picking up fetal distress

- Monitoring can tell everyone if the baby is in distress
- The heart rate of baby is something to watch at different stages in labour
- Keep track that the baby coping with birth
- Ability to identify problems quicker
- Early identification of potential distress, able to try less invasive methods of relieving her distress.
- Picking up distress e.g. cord wrapped around neck too tightly etc.
- Reassurance baby was safe
- Able to know baby is ok
- Reassurance that baby is well, assessing any medical concerns
- Lessened my anxiety about the baby
- Being able to pick up on issues with baby quickly, I was induced with cascade of intervention leading to ECS, would not have ended well for my baby without monitoring
- Reassurance that baby is coping with labour
- Reassurance that baby was coping in labour
- The midwife knows that baby is handling the contractions well.
- Important when interventions that can impact on fetal wellbeing are used
- Almost nil
- Mainly the baby's heart rate
- Knowing how bubs is doing but that can also be distressing
- Reassure the care provider
- Heart rate, BP can be looked at for both mum and Bub. In the hope to reduce harm and prepare for an emergency delivery.
- Peace of mind if you are worried about your baby
- Reassurance about the fetus' wellbeing
- To reassure me and my midwife team baby is not in distress
- Foetal well being
- Can listen to baby.
- To keep any eye on how bub is travelling during labour
- Gives information about how well the baby is coping in labour
- Knowing my baby is not distressed
- For my labour it was safety for baby as he had poor Dopplers and was small
- I suppose it may be helpful to detect uterine rupture. It benefits mainly the hospital system
- I know how the baby is doing
- Knowing that my baby was not in distress and all was well as the day went on
- If there are concerns with baby but think this can be better managed with intermittent monitoring
- Potential identification of concerns relating to fetal wellbeing
- Checking on feral stress to ensure urgent procedures-I.e Caesar/ epidural/forceps need to be discussed.
- Hand held and Doppler would be ideal- unfortunately midwife workload and policy over people prevents it
- Not having to worry about baby during labour

- Being in control of what's happening and that baby and mum are ok
- Baby safe
- If there are decisions to be made it is a tool to provide more information about what to decide. Eg if baby is showing signs of being stressed it may help you decide to have some intervention
- Yes but not continuously with wires and straps.
- Babies HR is constantly being listened to
- Knowing if there is an issue with the baby and being able to identify and act quickly.
- Can be reassuring to know that baby is coping with the stresses of labour.
- Being able to see bub was in distress and make decisions based on that.
- Seeing how bub is
- Helps the midwife
- Timing contractions
- Able to see if/when baby was showing signs of distress
- Keep an eye on baby well-being
- Fuck all!
- None
- I guess it adds to the available information for decision making.
- For me it was peace of mind. Especially in my second birth after the first saved my sons life.
- Helps manage some risk factors.
- Reassuring to know bub is happy
- Ensuring babies heart rate is not dropping too low, giving useful information about the safety of the labour for my baby
- Aware of baby's well-being
- Knew my baby was fine
- Able to help tell when a contraction is coming and make sure baby heart rate isn't terribly low
- In my case there were no benefits
- Instantly alerted to baby distress
- Continuously monitoring baby's heartbeat and contractions is very reassuring that the baby is okay
- Knowing babies heart rate is a normal rate
- Can identify any issues or distress in baby in real time
- Insight to bubs heart rate, stress levels
- You can tell if there is serious foetal distress
- Able to monitor my baby
- If there is fetal distress its apparent
- My child's heart rate
- It's hard to answer specifically because intermittent monitoring was beneficial, CTG was not. Benefits are reassurance baby is well.
- understanding babies condition
- To know that baby was safe and being monitored after heart rate dropped. Let me focus on staying calm and labouring/birthing without worrying if baby was on.
- Making sure baby is not in distress during labour

- I was reassured my baby was doing well, gave me peace of mind and also ensured everything was ok when my babies heart rate dropped suddenly after my waters were broken. I was not monitored through my entire labour but for some periods when there were concerns about baby.
- Information about baby's heart rate in real time
- Knowing when I was having contractions; monitoring baby heart rate and moving to vacuum delivery due to fetal distress
- Knowing baby okay
- To show to them he was still alive
- Reassure you baby is ok
- My contractions from the syntocinon were irregular and prolonged, were able to tell when baby started to get distressed by it
- Health of baby
- To ensue baby was okay
- Reassurance, data on baby.
- Knowing baby is ok
- Everyone is able to watch how baby is coping
- Monitoring fetus wellbeing when being induced
- I wasn't able to feel my tightenings so it was good to see them happening regularly on the monitoring
- Being able to see the strength and length/gap of contractions
- knowing baby's heart rate is okay
- I felt safe knowing that there was a way of knowing my daughter was okay during a very quick labour
- Reassurance that baby is ok
- Reduce risks of baby being stresses
- Constant check of heartrate, ability to spot things going
- I guess its reassuring knowing if something will happen to baby
- To ensure the baby is safe
- Fetal distress being picked up I guess.
- Maybe for the Mum to be to have peace of mind
- Intermittent monitoring with a doppler is fine in conjunction with highly skilled physiological birth care providers
- Fetal outcome
- Confidence in continuing to labour in my own time.
- Live interpretation of foetal oxygenation
- Reassurance baby wasn't in distress
- To ensure that the babies heart rate doesn't drop.
- Keep an eye on baby's distress levels
- Making sure baby is coping with labour, reassurance for the mother, hand held Doppler monitoring allowed me free movement and to be in water
- If you have been subject to intervention which prevents normal birth
- Overview of fetal wellbeing
- Reassurance if all is well with baby which was my situation
- Reassurance the baby isn't having a hard time for any reason. But we can also tell that from external factors so, maybe there isn't much benefit

- It was reassuring at times to know baby was being monitored
- If normal pregnancy and normal baby movements, minimal monitoring needed
- Unacceptable Bub stress .. ?
- Could provide reassurance for some people, ie in an induction that babe is coping with the synt
- Knowing bub is OK.
- To have a baseline & frequent readings of babies HR. To see how baby Copes with contractions
- Reassurance to parents of baby's health status during labour
- Know if the baby is in distress
- Making sure babies are ok
- Is baby alive
- Hearing baby heartbeat and knowing they are ok
- Reassurance
- Checking that baby is ok
- To ensure the safety of baby
- Knowing baby is tolerating labour well
- Knowing your baby is safe
- Able to keep a close eye on baby. I really liked knowing heart rate of myself and baby it out me at ease
- Through monitoring my baby's heart rate and the decrease of her movements they were able to understand that she was in distress and scheduled a c section very quickly. She was quite an unwell little baby when she came out. Without the monitoring they might not have known as quickly.
- Knowing baby is ok during induction
- As a standard in a low risk labour, I actually don't think there are any benefits of being monitored, and research is showing it doesn't improve outcomes.
- If your baby goes into distress it is picked up faster.
- Heart rate near the end of labour meant that ise of suction was recommended and was an easy decision
- Safety for heart rate of baby?
- It's great to monitor the bub and ensure their safety- however if mum and bub are healthy and Labor is progressing well, I don't think it needs to be continuous.
- They found baby was in distress
- Ensure baby's safety.
- Knowing baby was ok
- If she was becoming distressed, I wanted it be picked up immediately, not 30 minutes later!
- Checking on baby stress during labour and earlier intervention when required. Especially for labour that has been induced.
- If baby is seemingly unwell or not coping with the labour then monitoring may be beneficial.
- Pick up distress
- I knew my baby was in distress or not
- Reassurance that baby is coping
- That the staff can see if baby is in distress

- Knowing when my baby's heart rate dropped to indicate need for vacuum
- Informed decision making with at risk labours
- Reassurance
- Regular check on the baby
- Benefits are mostly for baby to tell if they're in distress, sometimes parents like the peace of mind of seeing the monitor
- ensure safety of baby
- Knowing how baby is coping
- Knowing my baby was ok
- Being able to check on how baby is coping during labour
- Checking on baby
- Know if baby is coping
- Reassuring
- Reassurance about baby. Could see labour was progressing and when it stalled.
- A/a
- Making sure baby isn't going into distress
- Keeping a track of baby's heart rate, fetal distress and my contractions
- Having a better idea of how baby is going
- To know when to push. I arrived at the hospital and had baby out within 5 minutes
- Informed decision making; reassurance; doctors and midwives were able to closely monitor
- If you need reassurance or there is a know. Justification/risk or complication
- Other than for baby's safety, none
- If there had of been a problem I guess it would have identified it
- Any changes can be picked up quickly and dealt with
- Picking up fetal distress
- Sound of baby's heartbeat assisted in mindful counting of breaths
- Information received as to health of baby, coping with labour
- My baby is safe
- Early signs of baby struggling
- I have no idea
- Checking on baby's heart rate
- Awareness of babies state/health
- Helps the team to know if baby is okay or distressed
- Check that baby is safe
- My baby's heart rate was dropping regularly during my labour. Being able to monitor it was very important
- Check in baby
- Knowing the baby is ok and not getting stressed by the labour
- It put my mind at ease throughout my labour
- Knowing the baby is handling the labour
- Reassurance
- We could quickly find out the baby's heart rate's dropping.

- Comfort knowing baby is not distressed. Would be so awful for anything to go wrong that could have been prevented if monitored properly. I do think its important
- None
- My midwife knew exactly how my baby was tolerating a syntocinon infusion
- Fetal Heart rate dropped and emergency c section was needed. This wouldn't have been known without monitoring.
- To ensure baby and contractions are safe and consistent
- To know that baby was okay. I wish I had been monitored in my first birth.
- We knew when we needed to get baby out immediately once she was distressed
- Knowing that the baby is okay.
- The CTG saved my baby's life and meant we could react quickly when baby's heart rate dropped out. The position I had been in must have restricted baby's umbilical cord. Without the monitor, we would not have known and the outcome could have been fatal.
- See how baby is coping with contractions
- For me, once I was in labour it showed that bub wasn't happy- leading to my emergency c. She had her cord wrapped around her neck. It meant that was picked up early in my labour so they could intervene.
- Baby's wellbeing is clear. My Baby's heart rate dropped and didn't come back up (cord around neck). If he wasn't being monitored we wouldn't have known and his birth may have had a different outcome
- It picked up that my baby was in distress so I was sent for an immediate emergency caesarean. As it turned out I would not have been able to give birth naturally due to multiple fibroids that hadn't been picked up during the pregnancy
- You know how baby is coping with the labour
- Safety of your baby.
- Safety of mother and baby
- It saves babies lives
- Without it I would not have my baby as his heart rate suddenly dropped to very dangerous levels
- They could observe babies abnormal heart rate
- Yes if you are high risk
- The baby's welfare
- Monitoring detected my first babies heart rate was slowly dropping. Because of Monitoring, the ob was alerted to a major drop in heart rate and he was able to save baby.
- Make sure baby is okay and not in distressed.
- Knowing how happy baby is during labour
- Picked up issues with heartbeat straight away
- Having a gauge of babies response to labor
- Information about baby / mother enabling decisions to be made in their best interests
- I'm not sure
- Tracking baby during labour
- Sudden changes in baby's condition are clear and able to be addressed

- Babies health
- Looking for well-being of baby
- Ensuring baby is ok
- Risk assessment
- Distressed baby
- When not being able to feel bubs movement you could be able to hear and see the heart beat of bubs
- They were able to adjust the oxytocin accordingly, and close to baby's delivery they were able to identify fetal distress
- Knowing baby was not distressed
- Safety of baby
- Aware of how baby is going, my baby went into Distress without monitoring I would not know this.
- Only for medical staff
- Assured that baby is stable
- Able to pick up an emergency easier
- Good indication of fetal health, is not painful and helps make informed decisions.
- To ensure baby isn't in distress
- Health and risk of baby
- Knowing the baby is safe
- Checking the baby is coping with labour
- The nurses can make sure that the contractions are getting stronger and that you are getting closer to active labour. They can also make sure that the baby isn't getting distressed.
- To monitor babies heart beat during contractions
- With the right practitioner, they can determine if baby is having problems
- To know the baby is ok
- I appreciate hearing my babies heartbeat on the Doppler intermittently - helped my motivation for birth. I didn't like being constantly hooked up to the machine. Knowing now that babies heart beats fluctuate (especially when they are nearing the birth canal) it was hard for my husband and mum to see the constant monitoring.
- Check on the baby
- Knowing that my baby was in distress allowed her to be delivered safety. I was stressed about the meconium in my waters and my labour not progressing so was relieved to deliver her safely
- Benefits would be to high risk women only, who have had abnormal traces pre labour and suspected foetal issues
- Knowing whether baby is in distress or not
- Reassurance
- To check you and baby are ok
- Reassurance that the baby is ok, and the ability to act quickly if something goes wrong.
- Monitoring baby for any changes
- Ensuring health of baby
- Was good to know how baby was tracking and if anything changed the care providers discussed options and kept me informed

- They were able to pick up on my baby’s heart rate dropping
- Intervention if something is not going well.
- Baby and mother can be observed over time to detect changes occurring
- making sure bubs was doing okay
- Keeping an eye on the babies heart rate gives the midwife and obstetricians a better understanding of what's going on and if they need to intervene earlier than anticipated
- Constant insights about your situation
- I don’t believe there are any.
- Able to monitor baby during induction
- Reassurance the baby is ok
- Nurse can respond if heart rate drops
- Checking if baby is in distress
- Could hear and see baby’s heartbeat beat do knew he was okay
- Warning of any issues affecting baby (like dropping heart rate)
- Hearing the heartbeat for labour purposes
- Seeing baby’s heart rate and seeing her stress levels through contractions over time.
- Peace of mind
- Easily depict whether there is a fetal heart rate deceleration or tachy
- Reassurance for mums who want it, picking up abnormal heart rate in rare instances
- Baby's heart beat fell dangerously low and this was detected quickly due to monitoring.
- Monitoring baby
- Knowing what is going on
- Can monitor your see if bevy becomes stressed or distressed during labour
- My baby was having decelerations, i knew he had to be delivered quickly so i focused on pushing,
- I felt very reassured to hear my baby's heartbeat during labour and knowing that she wasn't in distress or had slowed due to the morphine or epidural
- A well trained midwife can hear subtle changes in the FHR which may help with some decisions needing to be made
- An accurate trace of baby
- It regularly checks baby andy heart rate which could show if baby goes into distress
- Keep track of babies heart rate
- Keep an eye on heart rate
- They can monitor bub closely
- Safety of my babies health & well being
- Its reassuring to hear the heartbeat. Able to monitor baby's heart rate, and distress levels.
- Ensuring the safety of the baby and being able to react more quickly if things start going wrong.
- Reassured that baby was safe
- Keeps close eye on baby heart rate
- Checking stress of baby
- Easier to ensure that your baby is ok throughout

- I could here bubs heart and the sound sort of distracted me from the pain
- Able to detect changes quickly
- Yes, intermittent monitoring in a low risk labour is a helpful reassurance that baby is coping with the stressors of labour and birth.
- Knowing baby's not in distress
- Checking on the baby was reassuring
- I can't think of any
- It has its place. In some circumstances if the data is interpreted properly it could save lives.
- Able to be reassured everything is ok with baby
- Early detection of issues with baby during labour
- Safety of baby in a high risk pregnancy when indicated to produce benefit over risk
- Good to keep track of baby's heart rate
- Make sure bub is okay
- Safety of baby
- Reassurance that baby is doing well, identifying when baby isn't doing well
- Clearly and quickly know if baby is in distress
- Catching any issues with the baby
- A small glimpse into the health of baby when you can't see what is going on. Assisted my calmness in not trying to hurry things along and helped reassure me that my decision to not allow more intervention was not detrimental to my baby
- Status of baby in difficult circumstances
- Knowing whether baby is in distress
- Knowing how the baby is doing
- Ongoing risk assessment if baby heart rate or response to inducing drugs changed suddenly.
- If medically necessary can help to save baby.
- We could tell the baby was ok as I had to push for a very long time.
- Ensuring baby is not dangerously distressed.

- Able to catch fetal declerations
- Reassurance, able to intervene or take measures if mum or baby is not coping.
- Knowing that baby is ok. However, it can be quite stressful when you can hear it either slow down or speed up significantly
- Baby's safety
- Knowing that baby is ok
- Safety and well-being of baby and myself
- I think it can provide data that can then help the birth team and mother make an informed choice about birthing options. I think it can reveal/ alert the birth team and mother to potential problems (like the baby being in distress).
- The midwife could detect my baby was in distress
- Being able to tell if the baby is becoming distressed at all.
- Knowing how my baby is coping with labour. She had low heat rates throughout so it was good to monitor.
- Can pick issues up quickly
- Monitoring bubs incase they go into being destressed, raised heart rate

- Able to see how baby is
- My care providers were able to tell what both babies were doing throigh the whole labour. At no time was either baby distressed which helped me relax
- Know if baby is distressed
- For my previous birth while pushing my daughter's heart rate kept dropping, resulting in the Dr suggesting an episiotomy. I was happy that we were aware she was in distress and were able to respond to that.
- If the monitoring is accurate, it might provide some assurance that the baby is okay.
- My baby ended up being distressed and it was decided I needed assistance to birth. Without the monitoring they wouldn't have known that.
- Bub survived because they were forced to no longer put it off taking me to get cut open. I was bumped 3 times before I made it into surgery
- Knowing baby is okay
- Picking up on any distress for the baby, elevated bloody pressure for me.
- The monitoring saved my baby's life - it caught a dangerous deceleration of heart rate
- The safety of the baby.
- Safety for baby, detection of deterioration
- Hearing baby's heart rate is knowing he is well !
- Having an idea of how my baby was handling labour as we were hoping to avoid a csection
- It gave me comfort
- Knowing baby status, able to make informed decisions based on data, one scale to measure against
- If there are known risk factors, then it allows for timely adjustment of appropriate care.
- Knowing how the baby is. Calm
- Keeps a close eye on baby to make sure they are ok
- Knowing how baby is coping in labour
- Reassurance
- Knowing if the baby was in distress.
- See the babies heart rate and how they are coping with contractions.
- Can pick up when baby is struggling, can provide reassurance to some women.
- Literally only was useful 12 hours into labour when babies heart rate did start to have decels
- knowing how baby is doing and being able to adjust labour positions- when baby's heart rate was high I stopped pacing and stamping and instead lay on my side with a peanut ball thingy
- My baby survived birth because of the internal monitoring attached to him.
- Staff able to get an idea of how baby is doing inside, identify prolonged decels etc.
- Ensuring baby's heartbeat remains stable
- They were able to keep a close eye on my son who started to have some major decells during contractions. My body also wasn't going into labour and that's why they decided to have me go for an emergency caesarean
- Check progress of labour and heartbeat of baby
- Keeping an eye on baby to make sure they are coping with the labour

- I felt reassured that baby was well and coping with labour when she was listened to every half an hour.
- I don't think there are benefits of CEFM in normal birth, but I think reassurance (for mother and midwife) is a benefit of intermittent doppler/pinnard
- Knowing she was safe and everything was going well. Also made it feel so real that I was about t have my first baby and gave me motivation to get through the pain of labour.
- Reassurance to the mum; easy access to midwife and doctors as to what is happening with baby
- Parental reassurance, and it is an important indicator of baby's status for midwives/doctors
- As above, knowing baby is safe and can address issues early
- Track heart rate. Baby's safety
- So proper emergencies can be identified
- Able to easily see when baby is in distress
- That's babies heart rate can be carefully watched as well as contractions
- Checking the baby is ok and not in distress also helped me with contractions
- Good for monitoring contractions during IOL
- If the baby's heart dropped you'd know
- Prolonged labour and ensuring nothing has gone wrong, being able to ensure baby is still coping
- Know if something is going wrong
- Nil- particularly as I was in second stage already and baby was born 18 mins after entering the birthing suite.
- I think it helped prevent further interventions (such as a c-section) as they could tell the baby wasn't too stressed.
- That you know that the baby is ok and that of something goes wrong they can quickly realise that.
- It was good for my baby's safety
- Being able to monitor how baby is coping with labour.
- Knowing how baby is going continuously made me feel reassured
- Yes in case of changed in heart rate
- Reassurance
- Constant fetal surveillance
- Obvious ones like if baby is in distress
- Fetal distress
- Keeps baby safe
- If baby is in real stress
- Being able to see how well baby is coping during labour
- To check that I had no early signs of uterine rupture
- Yes
- Knowing what's going on and being able to intervene when things are going wrong
- Reassurance baby was handling labour and was well
- Reassurance surrounding foetal distress
- Can be helpful to identify fetal distress with intervention
- Knowing how baby is doing

- I liked that I could regularly hear the heart beat and that I could control my contractions based on the monitoring.
- safer for baby
- We were able to monitor baby and make decisions that were best for her in real time
- Not worrying about the baby, I could just focus of the labour.
- Keeping an eye on everything as I am a FTM I had no idea what to expect. However worried about impacts of vacuum and electrode on my baby after birth.
- Seeing how the baby handled the stress from the contractions
- Seeing the baby's heart rate
- Eyes on baby at all times
- I could see that my baby was well and healthy, I could move and go in the bath with them
- Helped me to know my baby wasn't in distress
- If u think it's the best thing, you will do that without knowing that you can access benefits from alternative options if they had been known to me
- Progression of labour
- Knowing baby was ok and heart rate monitored for distress
- Fetal well-being, particularly in high risk situations and prolonged labour
- Checking baby
- The benefits are only for the health providers
- Knowing baby was ok. Helped my OB make decisions about intervention. Ie she used the vacuum at the end in response to baby's heart rate dropping.
- I guess they would know if my baby stopped breathing and it was an emergency
- Intermittent monitoring I believe to be somewhat beneficial for picking up decelerations to baby's heart rate where it doesn't recover after contractions
- Reassurance that baby is coping.
- reassurance the baby is ok
- Baby safety knowing if baby was in distress or tired
- Picking up changes in babies status
- I think it's important to know that baby is doing ok so we can make changes if needed
- Knowing if there is a major concern with baby heart rate
- If needed for reasons for the babys health
- For me it was to ensure the safety of my babies as they were pre term and making sure there was no feral distress.
- Reassurance
- We knew that babies heart rate was dipping during contractions. He had the cord around his neck so it was an emergency
- Ability to monitor for fetal distress.
- Detection of serious adverse events
- Wellbeing of baby.
- Monitor baby's wellbeing
- Know baby is ok and not too stressed
- I felt better due to no longer feeling movement
- It did help keep me from worrying about baby's heart rate

- I know monitoring can be beneficial for monitoring fetal wellbeing during labour.
- Picking up a baby in distress
- Monitoring baby’s heartbeat
- If you are induced and labour is forced I believe monitoring needed as baby is shocked into birth
- That the baby can be monitored continuously when there is a need.
- Indication of baby not coping because something is wrong, such as uterine rupture
- To keep baby safe
- Knowing what is going on with baby and perhaps early indications of issues?
- Knowing that the baby is ok and to keep going.
- Can see if something is happening with bubs heart
- Making sure baby is comfortable and not distressed
- Can provide some comfort to an anxious mother, can allow medical providers the information they need to help the mother make decisions
- Baby was distressed which led to C-section, otherwise we would not have known this
- Knowing Myself and the baby were ok, it was backed up by the monitoring which meant i could continue to labour without further medical intervention
- Monitor bubs hr
- Could constantly monitor baby
- Baby's wellbeing
- Reassurance, early intervention if required
- Baby was being monitored
- I could hear his heart beat during the whole labour which put me at ease
- Able to pickup on deteriorations in baby quickly
- Health of baby
- Knowing what’s happening
- Knowing if foetus in distress and if needed emergency caesarean
- Ensuring heart rate ok no stress on baby
- I think if the baby needs it it is beneficial but mums should know more info about their options and how it will impact their ability to move during labour
- Can ensure baby is safe
- Ensuring all was okay with my baby
- Knowing baby is ok
- Safety of baby
- Knowing if the baby is in distress or not
- Reassurance that baby was okay throughout the whole labour experience.
- May pick up problems in labor
- So that the care providers can see some lines and hear some beeping. (Ensuring the baby is not tachycardic or experiencing bradycardia or deceleration, to monitor for foetal stress and to ensure foetal wellbeing)
- They could detect if me or my baby were in distress
- Small babies heart hate could be monitored. Unsure it was needed the entire time, though.
- In case there was something happening for baby

- Safety for baby, my baby’s heart rate was dropping a lot during contractions which wouldn’t have been picked up without monitoring
- Peace of mind - i knew the baby was ok so could relax and focus on delivery
- Knowing that baby is safe while birthing so the focus can be on the labour birth
- Constant supervision of my baby's health
- It puts the Doctors minds at ease and they feel a sense of insight into the wellbeing of the baby.
- In some instances, catching fetal distress
- Reassurance that the baby was okay
- Any potential issues with myself or baby
- Know it’s alive
- My first born could have died. I feel at least intermittent monitoring is important
- Ensuring baby is comfortable and happy
- Knowing exactly how baby was doing helped ease my concerns
- Making sure baby was always okay
- Baby safety
- Reassuring
- Knowing babies condition
- Reassurance, ability to intervene quickly if problems arise
- Knowing the midwife was keeping an eye on how my baby was coping the whole time.
- You can hear the baby's heartrate, assuming the midwife has actually found the baby's heartrate.
- Being able to detect if baby is getting stressed, so can take appropriate action
- Knowing when contractions are coming.
- Early warning of fetal distress, monitoring for effective contractions
- Doppler eveey 10mins woulf be more of benefit to me
- Monitoring for signs of fetal compromise, maternal HR/BP
- Baby’s heart rate can be monitored
- Real time feedback on how baby is coping with contractions
- If something was to have gone wrong we would have hoped to have seen the changes
- It alerted care team that baby was not coping with labour
- being aware of how my baby was going
- Knowing the baby is okay, gauging movements
- Where interventions has been use
- Heart rate changes
- Ruined my labour as i ended up having to just lie on the bed
- Ensuring the baby is not distressed I am sure - but I do not believe this needs to be through wired continuous monitoring
- Seeing that baby is ok during a stressful labour
- Keeping the baby safe
- Ensuring the baby remains well during the labour and being reassured by this. Being able to identify early if the baby becomes unwell..

- If there is a clinically justified reason for CTG then it is beneficial to healthy outcome for mother and baby
- Constant information about foetal health
- Reduces the minimal chance of danger/risk for baby. Useful to those with high risk medical conditions.
- If there is an issue withe foetal heart rate, you have a clearer idea of that
- Monitoring foetal distress
- Monitoring baby’s health
- Check baby is okay
- For high risk women and babies I can see benefit. For low risk women with a normal progressing birth I do not see any benefit.
- When they don’t break waters properly and make you go into labour for longer than necessary so baby does go into distress the monitor picks it up
- Benefits are in intermittent monitoring in my opinion, not in continuous monitoring. But they are sense of ease that baby's heartbeat is fine, and confidence to continue to labour and work hard knowing they are fine.
- Checking baby is progressing and safe
- They can make decisions more quickly that protect baby
- Can assess fetal distress
- More insight to baby
- To keep track of baby’s heart rate. My daughters Harare had big dips in it and this helped the midwives with knowing how to proceed with my delivery
- To keep track of baby heart rate
- Make sure baby is ok throughout
- Checking on the baby’s well-being
- Keeping track of Bub in destress
- Babys safe
- Knowing the baby’s heart rate at all times is reassuring, knowing they are not distressed, and knowing whether they are awake or asleep is also helpful.
- Babies well being tracked

- Continuous monitoring meant they could pickup when bub started to get distressed and had a better idea of how often I was contracting once I had the epidural.
- Hearing a strong beat, able to calm myself during labour, prepared for being ready to move into another way of birthing imcase of emerency. Importsnt Kept my partner calm, he said the sound kept him level and reassured
- Ensuring your baby is safe and the team have an idea of what is happening interuterine, especially while being induced.
- The birthing team could move quickly if my baby became distressed during labour
- Because I had experienced it before, I knew what we were listening to and seeing on the monitor - so it was reassuring.
- Information
- You know how baby is going
- Ensuring baby stays safe

- When you are still able to move around and monitoring does not get in the way of your flow, monitoring is reassuring to both mother and practitioners as we all know how bub is doing.
- Knowing the baby's heart rate
- So they know if anything goes wrong, it does provide a little reassurance
- Reassurance (mother) and more information to make clinical decisions
- That they were able to tell the changes to the baby's heart rate and do something about it before my labour progressed any further
- Nothing
- If there were any risks or irregularities in babies heart rate, coupled with health risks of the mother, then monitoring is important and necessary. It should not be used as a crux by health professionals rather than combine their skills and spend time with the mother to determine how the labour is tracking.
- Perhaps early awareness if baby is distressed
- Keepibg a close eye on baby to ensure we could continue natural labour
- My babe needed time be delivered quickly and I'm unsure if that would have been picked up as quickly because our heart rates were very similar and without CCTG monitoring it may have been confused
- I don't think there are any benefits
- I was 10cm dilated when arriving to hospital and had to lay still while having big contractions
- An indication the baby is ok
- Ensure baby is coping during labour
- Ensuring baby is coping well
- Could tell when I was having contractions
- Foetal safety
- Bring able to anticipate potential issues
- It puts the Doctors minds at ease and gives them the illusion that they are in control of the birth and have insights into the condition of the baby.
- Knowing baby is ok during labour

2. **What do you think are the downsides of being monitored?**

- I constantly needed to hold the monitor to get the babies heart rate.
- Distraction for staff and women, open to interpretation, can cause unnecessary worry and panic
- Difficulty moving during a contraction. -potentially leading to unnecessary interventions
- I found that it limited my mobility somewhat and I really like to move around during labour
- The excruciating pain- I felt like they were cutting me in half (compounded by the posterior position of the baby)
- Reduces mobility, focus on that as opposed how woman is feeling, can get obsessed with the monitoring as opposed to trusting body
- I was unable to move and as the wireless ones were flat I could not have a shower

- Difficulty mobilizing and losing the trace intermittently became distracting.
- I experienced continuous monitoring in a previous pregnancy (5+ years ago) which resulted in restricted movement, restricted access to my choice of pain relief (movement and water), added stress and a lack of clear understanding of the purpose/meaning of the results which caused anxiety.
- Lack of movement, feeling like you're on a time limit, not being free to move or get comfortable, not being able to use the shower
- Lack of movement and anxiety over hearing any changes in sound. Assuming the worst.
- To start due to my size the straps and monitors wouldn't stay in the right position. I was happy when the midwife suggested the scalp monitor
- Restricted movement and options
- It has the potential to freak everyone out! And its uncomfortable being continuously monitored in full blown contractions.
- It is distracting when in labour, it can lead to interventions that aren't necessary, can cause anxiety for the mother or staff
- Sometimes monitoring interrupted my head space
- Unnecessary intervention
- It's very uncomfortable, it's difficult to move freely
- It was so frustrating being connected up. Needing to stand still while they fussed. It takes the teams attention away from the birthing mother. They can become more focused on the numbers rather than looking at and being with the woman
- pressure for the mum and partner
- It was uncomfortable to move (noting though that I also had a syntocinon drip and IV antibiotics); issues when movement cause sensors to lose contact.
- Unsure
- Inability to move freely
- Lack of movement. Can be invasive. "Screen watching" for mother, support people and midwife. Focus on monotony trace being perfect all the time rather than trends
- Not very good at detecting whether the baby is in distress. It's uncomfortable and restricts movement, which makes it harder to cope with labour. Being struck on the bed increases likelihood of complications/interventions, including/especially caesarean
- Not really shown to reduce any bad outcomes, sometimes I had to reposition myself but this was minimal as I had a quick labour
- Not being able to move freely!
- More in relation to the epidural - I wish I was able to get a walking epidural. I asked but the anaesthetist said it wasn't possible.
- Lack of Freedom of movement, increased anxiety for birther, over medicalising a natural process when potentially not needed
- Unable to move. Time constraints. Decisions made based on times.
- In this case, I believe that the caregivers were too reliant on the CTG traces, even though the sensors were often dislodging and losing contact
- Limited movement and freedom allowed with wire monitoring.
- Anxiety about the trace, decisions being made based on trace alone

- I didn't have any down sides to having the auscultation my midwife was very good and I can only remember her presence doing it one time in my 4 hours labour
- Fear tactics used to force mothers to csections. Uncomfortable.
- Unnecessary intervention, could limit ability to move, create anxiety/stress for labouring woman
- The minute something sounded funny I felt a lot of anxiety and stress
- It made me very anxious, it limited my ability to move which possibly slowed my progress and made me feel little trust in my own body
- Nil
- The interpretation of the trace needs to be correct. It can be very subjective.
- Too much intervention, previous labour monitoring was used to coerce c section
- There are more likely to be unnecessary interventions
- Makes you feel limited in mobility. Also the choose of having wireless monitoring all depends on whether they're also correctly charged, or if in labour for an extended period of time, they run out of battery and then you need to be connected to wires and feel as though you must be on the bed
- I was stuck to the bed and couldn't move around at all. I felt I needed to stand up but couldn't. They were very concerned that they weren't picking up baby's heart rate and I'm sure I would have had some kind of obstetric intervention if I hadn't been isolated due to "suspected covid' (I had a cold)
- Where do I start? No movement, people watched monitor instead of me, no trust in my body or baby, noise, no movement
- Everything
- None
- Freedom of movement, interference during a contraction.
- Constantly being annoyed to find baby's heart beat when in the shower
- Everything. Restricted movement, higher adrenaline slowed labour, adrenaline meant I was in more pain and needed more pain relief. I wasn't allowed in shower or bath. More interventions for fetal distress that caused injuries to both me and my baby.
- Limited movement and access to water - not portable CTG
- Midwife and doctors focusing on the CTG instead of me.
- Intervention
- Restricted movement, no trust in woman, not accurate measurement of health of baby, trust in machine over woman, unnecessary decision for emergency caesarean
- None for myself
- Restricted some labouring and birthing positions (even with wireless they kept losing the trace)
- Inability to mobilise, difficulty with pushing sensation
- Anxiety, lack of movement and being unable to use water for pain relief
- Feeling i couldn't move as the straps were not holding the monitors in place. Once the electrode was inserted it felt odd having the wire hanging out and fear of pulling it off baby
- Very uncomfortable and restrictive even when wireless
- Restrictions in movement, clinical setting, uncomfortable, starts a cascade of interventions, impacts oxytocin

- Any small blips makes providers worried which can lead to intervention
- Overly worrying about timeframes of the hospital
- I think from my perspective monitoring was always beneficial if anything i think there should be an easier way to monitor larger women
- Having to stay still
- I found it anxiety inducing every time I saw the Midwife with the doppler in her hand preparing to listen I'd worry that she would find something abnormal and my lovely natural undisturbed north might end there.
- Not being able to deliver in the position I wanted
- It stressed me out greatly and was something that I feel contributed to a stalled and ultimately traumatic labour. They didn't explain that some changes in the baby's heart rate was normal so it would go down a little and I would get extremely distressed. Additionally in my experience they didn't get proper consent from me and that made me feel very dehumanised and uncomfortable which made things worse and more difficult for me
- I think it creates more intervention and stress and hinders the birthing person. Birth partners mag get fixated on the numbers and not be paying attention to the birthing person and the headspace of the birthing person if they are worried about the monitors may not allow them to get in the right head space for birth.
- Labour is delayed if movement/bath are restricted
- Unnecessary stress and potential for unnecessary intervention
- Less focus on the woman and more on the machine
- Constantly being brought out of your birthing brain unnecessarily
- Being stuck in certain positions, increased pain due to this.
- Limited movement and positioning.
- Being attached, less free movement
- Restricted movement, stresses mum out, constant monitoring is unreliable and can show many false results where baby is not actually distressed but monitoring says baby was
- Unnecessary intervention
- Lack of movement, and potentially over analysing normal patterns.
- It sometimes made me anxious, if the sound of the heart beat dropped out or the alarms sounded. I also think the inability to move freely for extended periods was certainly annoying
- Lack of movement, being stuck on the bed not good for baby position or mums comfort, distracting, not always accurately picking up, differing interpretations
- Unable to progress and move around freely
- Not being able to have a water birth, monitoring was distracting, kept falling off couldn't get in the zone
- Being asked to stay still during a contraction. Not being able to move around and labour properly if wired monitor used.
- Can lead to unnecessary interventions. Restrictions on labouring positions or environments
- Incapable of moving freely which is a major downside during labour. It was super uncomfortable and borderline painful

- The continuous, wired monitoring heavily restricted my movement. Being in the bed was uncomfortable and got in the way of my labour.
- Thinking something could be wrong with baby when the monitors aren't tracing properly
- Unnecessary monitoring can cause stress on mum, inability to move freely, and too much focus on the medical side of something that our bodies intrinsically know how/what to do
- Not enough evidence on interpretation, different interpretation by different care providers. Leads to fear and unnecessary interventions
- That's all they seemed to care about
- Not being able to walk around
- Can’t move around
- Added stress, restrictive movement, makes it unnecessarily clinical
- Continuous noise, worry when you hear different noises
- False sense of security. Impairs movement. Inaccurate. My CTG was very patchy due to my movement in labour so had a fair amount of loss of contact. Moved freely anyway
- The monitoring was showing baby’s heart rate completely fine. Yet my baby pooed while I was in labour
- Monitoring until failing and midwives coming to adjust things all the time when they dropped
- It’s awful if you can’t move. I couldn’t get into established labour and needed to be induced because my baby’s head wasn’t going properly on my cervix.
- Sometimes unnecessary action is taken when baby appears to have irregularities that are very well just normal physiological processes of labour, for example assisted births or unnecessary caesarean.
- Having to stay still during that time with then hand held Doppler although this was minimal
- Interruption of normal labour processes, inability to move freely, fear mongering when the machines don’t work properly (ie on posterior babies like mine)
- Inability to move freely. interruptions from the healthcare provider during birth to discuss babies monitoring.
- Requires adjustment, observation, need skilful interpretation.
- Limited movement, uncomfortable, having to readjust all the time, watching ctg and being anxious if a decel or anything abnormal occurs
- Inability to keep moving
- It interrupted my labour
- Less ability to move and use natural forms of pain relief, unnecessary provider panic, not evidenced based, does not improve outcomes, reduces provider skills in assessing labour
- Less movement
- Limited movement, increased pressure for more interventions
- Restriction of movement, labour processes, becomes primary decision maker, becomes not about the woman and the baby, intuition lost, heavy clinician reliance
- Limits positioning, uncomfortable , looks for abnormal amongst normal
- Maternal uncomfortableness

- Less flexibility in birthing positions and locations
- Cascade of interventions, likely caused my monitoring which may pick up up on relatively normal heart rate dips during contractions. The application of any monitor can affect the woman's mindset and ability to relax and progress naturally. Increases anxiety. I had to ask for the sound to be switched off because it was so loud right next to me and so fast. I was asked to change my position or stay still during contractions to be hand monitored and that increased me pain levels.
- Not having the results explained to you, being left alone in a room for a long period of time
- Non-wireless options don't always allow for free movement and to change positions easily
- The CTG would lose connection and need midwife to fix it which was annoying. That could be stressful for some people thinking something was wrong
- Having to change position so the midwife can hear the baby's heart beat
- They are not great at showing you when a baby is unwell, they lead to unnecessary intervention as ppl can't interpret them, lack of freedom and movement, and they use a central monitor where I birthed so ppl that had never met you and didn't know what was going on would come running in if there perceived something to be wrong
- I know it can be uncomfortable, distracting and lead to unnecessary interventions, but for this birth it was reassuring for me, and the midwives did a great job of making it comfortable and not limiting my movements/choices in any way.
- Inability to move positions, inability to access water (was refused by care providers), easier for care providers to limit you to the bed, can falsely interpret CTG recordings or use the reading to claim fetal distress, I believe it was part of what directly lead to my birth outcome being negative, it was also used to say my baby was getting "tired"
- Overrides the care of the mother
- No down sides to the way I had it done this time. Was unobtrusive, did not restrict me and was not painful. Midwives were awesome!
- Increased intervention, deceased upright movement, forced into positions, not left to intuition
- Unable to move freely, misunderstood by midwives when monitoring changes, leads to unnecessary intervention
- Not being as in tune to your own body during contractions, I focused on the numbers on machine
- Care providers overreacting to things like loss of contact and assuming it was a Brady
- No freedom of movement, unable to labour in the shower or water, no sense of control or say in own care
- Increased chance of intervention due to misinterpretation of CTG trace
- Misinterpretation of results leading to increased invention and care provider fear untrusting a natural process
- It was very tight and painful. I couldn't move around the room easily. I had no choice and felt disempowered. I had to have an internal monitor as the external was so painful but they didn't tell me that I had a choice to not use it at all or just do intermittent monitoring. The internal monitor meant that I couldn't use the bath which I had really wanted and I honestly think it was a factor in me needing an

epidural as it was hard to manage the pain and I felt disempowered which I think made me give up earlier

- I experienced no downsides.
- Less freedom of movement
- Couldn't move around as much as even the wireless monitor lost heartbeat a few times
- Feel like there's more pain in the labour and you get off track with breathing and concentration labouring it's like going backwards
- The cords were VERY distracting! The finger clip kept coming off and beeping would start.
- Very uncomfortable, straps very tight, staff pushing hard on the monitors tying to pick up a trace. Monitors slipped every time i tried to moved and had to be rearranged.
- Taking time from the labour progression to wait and check. In my first labour (10yrs ago) I was continually monitored, and that did impact on my ability to move around and made labour more difficult- horizontal bed position rather than upright.
- It doesn't stay in place so constantly has people fiddling with it and adjusting it throughout labour. I could see the monitor and found myself focusing on it and trying to decipher if it was normal or not. Because it kept slipping off and losing baby's heart rate, I felt I had to sit still and not move. It makes the professionals focus too much on numbers and not the woman's individual experience. It prevents water immersion.
- Inability to just do things without worrying if the monitors are still recording
- Restricted movement, unable to get into the water tub for pain relief
- None
- Uncomfortable, annoying
- Restricted movement, inability to labour as I wanted to, the restrictions slowed things down and we came close to an instrumental birth. In the end there was no need and the monitoring potentially put bub at risk because they were watching th trace and not me labouring.
- Potentially causing anxiety. Movement restriction.
- Not for me, but if continuous then not moving around and unnecessary interventions
- How long is a piece of string
- False readings, untrained care providers using monitoring to coerce into interventions and c-section.
- The discomfort or restriction of some methods, monitors not staying in place meaning having to place them back when the monitor goes off instead of fully focusing on labour
- Affects headspace, constantly being touched/ distracted, big chance of increased interventions
- Did not change my birth plan and outcome so no down sides for me
- Interrupted during labour while midwife tried to find baby heartrate
- Restricted movement of mum, could start cascade of interventions that might not be needed because heart rate naturally varies.

- It’s uncomfortable, it leads to more interventions, it doesn’t prevent or reduce infant mortality or morbidity, it increases c section rates, it limits choices of positions you want to labour in
- The baby moving and then lose the tracking
- Only downside was that I couldn’t try to use a shower/bath to help with labour symptoms
- I believe there is a confirmation bias. The more you look for a problem the more likely you will find kt
- None for me. I can imagine continuous may interfere with free movement, use of water
- '- takes midwife focus away from mother - potentially stretches shift midwives across too many mums - lack of understanding of results, large "grey" area means overly conservative interpretation of results, higher rates of unnecessary interventions
- Nerve wracking listening to every noise, baby would get worked up and send both out heart beats up, couldn’t move around and get comfortable
- Being restricted
- Care providers intervening, cascade of intervention
- Inability to move. Undue anxiety listening to the baby’s every change in heartbeat. Inability to go in the shower or bath.
- Interference with the progression of labour, allowing doubt or fear to enter the birth space, causing unnecessary alarm which can lead to temporary pauses in labour and a cascade of interventions
- Interrupts natural process, restricts movements,
- Restricted movement, pushback when asked to remove so a walk around the hospital was possible
- I get obsessed with watching the trace (but this is true for any and all data available)
- Decreasing movement in labour, causing alternative pain relief methods e.g. epidural as shower interferes with reading of CTG, constantly being touched during labour to fix CTG ultrasounds.
- Poor readings cause poor decisions
- Intervention can occur
- Can increase anxiety, birthing person focuses on monitoring results. Impacting maternal movements and therefore may lead to longer or more painful labour. Reduces birthing person's trust in their body
- Can be uncomfortable at times
- Unable to move, issues when they lose the trace
- I was in strong labour when I arrived in hospital so it was uncomfortable to lie down for them to monitor the baby so I just said "sorry I have to stand up" and I did and they just listened to the baby while I was standing up
- If volume not turned down, a bit distracting
- There are many downsides to monitoring that is not intermittent with a pinard or doppler. Mums need to be able to move freely in labour and delivery, use water for pain management etc.
- Many decisions for intervention are based on CTGs that don’t look reassuring, but often (usually!) the baby is completely fine. It prevents women from moving freely

and utilising the bath in my setting if there are any concerns for the baby abs if a fetal scalp clip is based. Midwives become totally focused on trying to get good monitoring rather than on providing good physical and emotional support to women. Much higher rates of caesarean and instrumental birth and more episiotomies are cut because the monitoring picks up decelerations (which are often normal and not a sign of distress but it's hard to tell which babies are coping abs which ones aren't)! The straps can be tight. The wireless often runs out of battery abs then women are tied to a machine. So many down sides!

- Uncomfortable and restrictive
- Not being able to move and if having a VBAC they use it against you as an excuse that if any little thing goes sideways you'll be rushed for a c section
- Being connected up left me very restricted
- Yes- can be restricting, can increase interventions, makes women doubt their own abilities and their babies. Health providers can become focussed on the trace and not the clinical picture
- Reduced movement and water access. Constant readjusting of monitors when I did not want to be touched during contractions.
- Interrupting a labouring woman
- Poor contact in the shower
- My experience of continuous wired monitoring during my first labour and birth (not referred to in this study) lead to a complete inability to move my body to support my baby to be born.
- Limited mobility
- Limits to free movement, added stress.
- It's super uncomfortable when you have regular contractions or are in an awkward position. Obviously, there would be radiation effects to the baby if used incorrectly or for long periods
- Unknown effects of US on baby. If CTG it can increase your chances of having a c-section. Can limit movement and positions depending on type of monitoring.
- Uncomfortable, restricted movement, midwives constantly repositioning the straps, not being able to hold my belly for the straps being on the way, they were extreme annoying
- Not being able to move! Having the midwife constantly touching it to adjust, it's uncomfortable. The doctors were focused solely on the CTG trace and not what I wanted.
- Annoying, uncomfortable, invasive| risk to baby when scalp clip is used, it can lead go unnecessary intervention
- Movement restriction
- CTG bands could slip and lose the trace so they were regularly being moved and adjusted by my midwives.
- Increased risk of caesarean; increased unnecessary medical interference in the process of labour which can increase mum's anxiety (emphasis on what's going wrong, not right); lack of access to water for pain relief
- Distracting, uncomfortable, poor sensitivity and specificity, increased risk of intervention
- None

- Strapped down- can’t move difficulty monitoring because I’m a size 16 so forced to have clip
- Intermittent monitoring with a doppler absolutes sucked. It was mid contraction, hurt, was distracting and I didn’t like people up in my face during labour
- Not being able to move much, but I had an epidural so it doesn’t really matter I guess
- Confusion between my obs vs. bubs
- It completely turns off the primal brain and the labour process and takes the focus off the important stuff. Mostly I would think it would slow down labour and cause more intervention. Plus it’s uncomfortable and annoying
- Absolutely. Limited movement which can slow labour and find comfortable positions, inability to use water for pain relief, extremely uncomfortable plus it kept slipping off and losing the heart beat meaning the midwife was interfering and disturbing the flow and progress of labour.
- Restrictive to mother
- You start from a very negative place. You feel like you have to perform in birth. Pre labour monitoring is also very stressful. I had a bowel movement and then returned to my monitor and all the alarms went off and the midwives scared the hell out of me saying they had to find my baby's heartbeat when she just in fact moved. the whole birthing monitoring after that incident was STRESSFUL.
- Can interrupt labour. Can cause unnecessary stress and prevent active labour. Can be used to justify unnecessary interventions. Can be done without consent.
- Its requires you to stop doing what you’re doing, maybe only a minor inconvenience but still an inconvenience. And well there are well documented down sides to CTG monitoring.
- Paying more attention to the monitor than the woman - if the monitor says not in active labour, but the woman is in labour, believe the woman.
- CTGs would easily lose the trace if I sat down, which made my posterior labour a lot harder.
- Cascade of interventions. Unable to move in to better positions
- Unable to move, relax, get comfortable or get in the zone
- Couldn’t move,
- I think it made the staff over vigilant to normal variations in baby's heartbeat. And that meant that I was asked to be in less than ideal position.
- Sore tailbone and being heavier set it wax annoying to have to hold the paddles in place to get trace for some long periods of time
- People will look at the output of the monitoring and not at the labouring person in front of them
- Can interrupt your focus a bit but my midwives were so respectful it wasn’t much of an issue.
- The scalp electrode seemed invasive. The CTG being unable to pick up the trace was stressful for me and frustrating but I couldn’t help moving around
- Further interventions, limited movement, dissatisfaction of labour/birth
- Uncomfortable. Can be cause for unnecessary intervention
- Too focussed on contractions and the numbers and baby heart beat which can be scary for a birthing mother

- Too many to count, too angry to go into the details
- Movement needs to be somewhat planned
- Lack of mobility, coercion from staff ie told I could use water immersion or shower so I took the monitoring off all together, being disrupted and asked to move to get a good contact, hospital staff look more at the paper on the machine than they do at you to assess your labour, hospital staff are fearful of mitigation so overreact and panic at normal physiological FHR patterns such as early decels in second stage when I had lots of pressure and tachycardia when I could feel my baby moving significantly / rotating. Failing to look at big picture
- Restrictions on movement and comfort during labour if wireless monitoring is not available, also for those women who find being in the water soothing this is not possible when being monitored
- Not being monitored properly as in my case, not picking up bubs heart rate correctly
- Beeping, monitoring makes you feel anxious, it’s another parameter which is used against you in the birthing room, another way to take away your choices and your power and make you feel inadequate
- Limited in positions at the end
- Labouring mums are restricted. We can’t labour the way we want or in a relaxing environment due to cords/machinery beeping and going off and constantly re attaching them when they move or get loose. I believe often its unnecessary and hinders what could be a positive, beautiful birth.
- I hated it, it was a distraction, it was uncomfortable and it didn’t allow me to move the way I needed to for my labour
- For CTG wired monitoring lack of movement, unable to access water, even tens machine was removed as apparently conflicts with CTG. Made me very scared & out of control
- being unable to move freely. unnecessary worry
- Harder to move around the room. I was still able to stand and rock, and north on all fours- but wireless would have enabled me to move away from the bed area.
- There were none for me. I had an epidural so was not moving around. The wires were somewhat irritating but in the scheme of labour and birth it was nothing
- I wondered whether the last bit of my labour got rushed because of monitoring results. She was 9/10 APGAR on arrival but I felt pressured to get an episiotomy due to baby distress. Afterwards I have wondered whether this was completely necessary
- It was very uncomfortable and restricted my movement
- Limited movement, had to birth lying down
- Not including risks no down side just want to be more involved
- Make you panic if the midwife struggles to find the heartbeat right away
- Wireless toggles would lose baby’s heartbeat when I moved around. So I was consistently having my midwife readjust it or ask me to stop for a few mins so they could check it.
- Anxiety inducing watching the monitor. I didn’t take my eyes off the monitor for the 10 hours I was in labour before the emergency caesarean
- Might freak you out if something is wrong but I’d rather know

- Too much focus on tech working/not working from midwife abs not enough focus on me. The data from the baby was also used as a reason to rush to instrumental delivery rather than allowing me to push on my own.
- Machine constantly malfunctioning, midwives in and out fixing it and readjusting, felt touched out
- There is an extra thing touching you during labour
- Unnecessary interventions
- Definitely stressful of considerable changes occur
- Not being able to be in the water
- Invasive testing, restricted movement, lack of access to waterbirth
- I had to come back to the room every 15 mins for a review
- Couldn't move as freely even though it was wireless, it would slip easily, machines would alarm and id have to wait for it to be repositioned by midwife
- Uncomfortable, felt movement was restricted. The fetal strap. felt heavy
- Freedom of movement
- can't think of anything
- Restriction in movement and having someone in your space at all times constantly adjusting the monitor and straps.
- Unnecessary interventions
- Creating chaos through looking too closely
- Loss of freedom of movement, care provider reliance, not evidenced based
- Painful to attach- FSE
- Often misinterpreted and impacted my ability to cope in labour when the machine malfunctioned
- If it was continuous/ not wireless, distracting during labour
- If you have to be continuously monitored it can be uncomfortable, less room to move around, more invasive in regards to allowing your body to birth your baby without intervention
- Slowing of my labour. More emphasis out on the CTG machine output than actually talking to me as the patient.
- Sometimes having to stop moving to get a good trace, hard to do when contractions are close together
- Machine becomes more important than the woman
- None. Reassuring to me and my care providers
- Stress for mother if baby shows any deviation from normal. Increased intervention. I'm my case it was coached pushing due to heart rate dropping at the very end. I didn't want to do coached pushing and believe if led to a vaginal prolapse.
- If there is continuous monitoring this can interrupt the natural process of labour, and also seems to increase interventions and csections.
- Stressful when alarms activated- loose connection, baby heart rate change etc, restricted movement
- Minimal monitoring is ok as long as one is allowed to move freely, being attached to machines and monitors feels very controlling
- If they can't find a heart beat â€¦ the level of panic or concern it could create
- Restrictions on movement! Overreacting of care providers to monitoring

- They kept losing bubs heart rate and it made if difficult to move into comfortable positions. Then when they were monitoring directly from bubs head I couldn't really move at all
- Cannot move! Therefore nonpharmacological pain relief options are limited. No shower or bath. Increased risk of infection with FSE. Midwife constantly adjusting belts & interrupting natural rhythm of things. Does not allow for optimal maternal positioning. Let me to get an epidural & therefore increase risk of instrumental
- At times care providers fuss around with the placement of straps etc if the monitoring device isn't clear
- Nik from my experience
- Very uncomfortable, could not move much
- Lack of movement, lack of trust, machines take priority
- Another thing to go wrong or add to stress
- Stressing when the machine indicates an issue
- Not able to move much
- Getting information which may not be useful, interrupts the flow of labour
- Intrusive, annoying, distracting. Checking occasionally via doppler would be much better.
- Not being able to move
- Sometimes monitoring fell off while moving around. It didn't limit me being able to be upright, was just annoying for the poor midwife to have to keep reattaching.
- Annoying laying still/being connected, but worth every second.
- Concerns when connection is lost
- Increased pain, anxiety, distraction. Loss of control and autonomy. Increased likelihood of interventions.
- Lack of ability to move around
- Limited movement. But I didn't have a need or want to move from the bed.
- Uncomfortable. Kept coming off. Had to be readjusted constantly
- Restricted movement. Hearing midwives talking about normal rises and falls in heartrate is not helpful to a labouring mother.
- Can't move freely, very restricted. Kept losing baby heartbeat and needing to be readjusted
- Less movement capability.
- I tend to move around in contractions, so I knocked the monitors regularly, which often set the alarms off causing some distress once the contractions eased
- I couldn't really leave the bed, I would have to ask to be unattached to go to the toilet etc
- Less freedom of movement. Higher chance of medical intervention if they don't like what they see that may or may not be necessary.
- Often leads to cascade of intervention. In my case, I was unable to move around, I couldn't have the water birth I had planned, I had to push on my back.
- No ability to move, slowed labour and angered me
- I could hear the impacts of contractions on my baby
- Being unable to move as freely, having the probes moved constantly was distracting

- Being stuck in one spot, all the wires and cords limit movement, it does add a bit more stress if baby heartbeat is erratic, sometimes it could add more intervention in labour
- None
- Limitations to movement at times
- So uncomfortable and not being able to lie in different positions
- For me a huge downside was unable to water birth in a bath, it is invasive and a little irritating when moving around. CTG straps kept moving and dropping out
- limited movement, hard to use water for pain relief, staff were more interested in the machine than me
- No bath
- It hurt and stalled my labour
- Not being able to move around freely
- Wired monitoring inhibited non pharmaceutical pain management
- Hard to get undressed
- Couldn't move
- Disheartening/frustration when labour was stalled. Unable to move freely/use water options.
- Distracting and could inhibit physiological birth
- My monitor continuously went off because it kept slipping, sending off alarms which made me panic about the well-being of my baby. The midwives didn't immediately respond to the alarms, which made me wonder why they used them. When I asked why it kept going off they told me not to worry, it was a bit stressful
- Restricting. Annoying noise
- Restricted movement. False sense of alarm and panic when monitoring drops out
- It can be restrictive
- Straps are annoying but that's it
- Not able to move around
- Significantly impacted on my ability to move around during labour, prolonged labour, had a very negative birth experience
- It was very stressful for me personally (as noted above)
- Being attached to wires, not being able to move much
- Being able to hear the babies heart rate drop was distressing, as well as not being able to move around.
- Restricted movement
- Become fixated on results
- I feel like there's a constant pressure to hit milestones during labour progression. Interventions offered at the first sign of distress.
- Nil
- Was very aware of any changes to heart rate
- No idea. I don't know anything about monitoring.
- Care providers could become too focused on input from being monitored that influences decisions instead of gathering a bigger picture
- Needed to be continually repositioned. Caused some distress/distraction seeing the monitor data

- Less mobility, less able to find a comfy position, my husband was busy holding the monitor in place when I needed other support
- Every time her heart rate dropped I would stress
- Not able to move.
- Being conscious or concerned about what the monitoring is showing. Being a bit more uncomfortable
- I focused on it a lot
- Lack of movement, not reliable information
- Not being able to move freely
- NA
- Not being given freedom to move or get into water made me frustrated and go instantly for the drugs to help cope
- Stopped me being able to move during my contractions and knowing that was my only pain relief/way of coping really bothered me which made things worse. Even worse was it was a student doing it so would take her a long time to find the heartbeat which just annoyed me more (never had issues with students before was just stupid being monitored for no apparent reason)
- Can impact mobility and st times becomes the focus for midwife
- Couldn’t use tens machine. Couldn’t move around
- Leads to limiting movement and fiddling with straps to impact on labour movement and progress
- You can't move where you like or stand up when you want.
- I was literally half naked in a sterile room trapped. I felt trapped.
- Mother can become stressed and worry too much about every little heart beat
- None. The slight annoyance of wearing the monitor was entirely worth it for safety of baby.
- No being able to move around stuck to the bed
- Interferes with maternal concentration, prevents water submersion in some cases
- For me, she wasn’t in a great position so it was hard to get a trace. I’m also a larger person (size 20) so they found it difficult to keep the bands on if I was moving around.
- Can’t move around as much, although the head monitoring was much better than the CTG
- Restricted movement
- You are restricted to an area.
- Potential to slow labour and not get the body into different positions
- Can restrict movement, can cause distress and can cause intense pain
- Restrictions, couldn’t have a water birth, having to carry around the machines, hard to get into comfortable labour positions, couldn’t place baby on tummy after birth due to all the monitors, pads and cords
- Unnecessary stress on straightforward births
- Too much intervention
- Even with wireless monitoring you don't have total freedom to move as the sensors keep falling down.
- Not being able to move around if you don’t have wireless monitor.
- Over medicalisation of labour

- Increased interference and intervention in labor. Medicalising birth. Uncomfortable and annoying.
- Restricts movement
- If the trace drops it creates extra stress for everyone
- Perhaps limited ability to move around but I didn’t experience this
- Anxiety around minor changes to baby's heartbeat
- Little communication
- Decreased mobilization, less focus on maternal care, less belief and support of mother, more medicalised
- Being restricted to move, more anxiety, focused on it too much, less patient connection
- Abuse of power
- Limited movement, very hard to manage pain laying down
- Being stuck on the monitors for a long time and having to start again if you need to go to the toilet
- Being stuck in the bed, limited movement, had to remain in certain positions to maintain an adequate trace.
- Being attached to a machine and baby moving a lot and trying to find a good trace
- I had so many wires, IVs etc I could barely move
- None
- I was uncomfortable and couldn’t move as much
- Can run the risk of intervention and can also lead to added discomfort in labour with needing to allow medical team to move you or disrupt you
- Restriction on labour meant stall in contractions
- Some movement restriction but benefits outweighed downsides for my experience
- Bring Bed bound
- Limited movement
- Being connected to something at all times
- Being unable to move, have a shower, change positions easily
- The monitor sighs into your belly and is extremely uncomfortable. The wired one makes it so you can’t move around properly and the wireless one kept moving down and almost falling.
- Limited movement for mum
- The restricted movement during labour, the potential for them to intervene unnecessarily if they don’t fully understand the normal HR fluctuations that occur during labour for mother and baby.
- Loss of mobility if hooked up to wires. If like me, they resort to a Doppler after refusing the wires, it interrupts your concentration and mental space. It also forces you to be in certain positions/stay still after every contraction when you should be using that time to re-focus and prepare for the next contraction
- As above
- Monitoring keep failing and cutting out
- Feeling constricted during labour
- So many issues. Increased risk of caesarean as decels could be interpreted as dangerous rather than a normal response to moving down the birth canal. It restricts

your movement, even wireless options as the staff are constantly fixing and rearranging the monitor if it loses contact.

- Some people may find it increases anxiety. I am someone who wants all the information and is not stressed by it but actually more relaxed.
- not being able to move, therefore increasing pain
- You are constantly concerned about disconnecting your monitors which inhibits your ability to move and every time you do move it loses connection making it beep which makes you concerned about your baby instead of focusing on your birth, definitely increases anxiety.
- I wasn't able to move around or use the bath. Although I don't think I was able to use the bath for other reasons
- Having to be in hospital for the entire labour due to PROM
- Worrying and listening to heart rate of baby change
- Was a little restricting at times but I had an amazing team who ensured I wasn't impacted too much
- Extremely uncomfortable I wanted to remove them but wasn't allowed. Hated labouring in the bed but was too difficult to move
- Impedes ability to move around. For me, wireless not charged. Could not get into the bath with the straps on. Gave incorrect data at times. Midwives relies too much on the machine output and did not listen to me that I was having contractions.
- None really, communication could be improved to take stress away
- Obviously being connected to a machine - I also didn't get to give birth in the birth centre at my hospital as there were greater risks
- A lack of freedom to move around, change positions if needed to help progress labour
- Can be more comfortable. Cannot think any downsides
- Restricted movement. Not evidence based. Focus on machinery instead of woman. Disrupts the flow of birth. Causes stress
- Monitor kept slipping and needed ongoing repositioning
- Birth is already over-medicalised, midwives and doctors monitor everything so closely that interventions are the norm
- None
- Reduced movement, feels very clinical, may increase anxiety for some patients
- It was uncomfortable even wireless. Moving could make the strap slip
- Potential extra stress/fixation on heart rate rather than considering the labour more holistically
- It was getting in my way of concentrating on labour
- Limited mobility or comfort, difficult to sleep/rest without bumping the monitors
- Yes absolutely!!!!! Interventions!!
- Increased interventions, decreased comfort, decreased mobility, use of water
- Less able to move around if you want to stay active during labour (i had an epidural and later a spinal block so not relevant to me)
- No
- Creates anxiety
- Somewhat negatively impacts mother's positioning during labour
- If there is no wireless monitoring it would be hard being stuck in bed

- Not being able to move as freely or use my TENS machine
- It’s very restrictive, the midwife has to spend time watching the machine not the mother, the CTG is open to interpretation
- Not being able to use the shower or bath
- You can’t actively move around how you want it makes you feel so restricted
- Uncomfortable
- Affected mobility and labour positions
- Limited movement
- It's scary when the machine loses the heart beat or it keeps dropping.
- Can't move around, midwives get annoyed if you move and the monitor slips, feels really uncomfortable, everyone focuses on the monitor and not much else, space in front of the machine was so cramped my partner was asked to press buttons on the machine, made it really hard to focus on mindfulness/breathing
- It is annoying to be checked continuously
- No freedom to walk when wired
- Nothing
- Occasionally slips off the right areas and needs to be readjusted
- I would think possibly more chance of intervention earlier than necessary
- Sometimes monitoring is over analysed and restrictive, occasionally leading to unnecessary intervention.
- Lack of movement, restricted movement
- Picked up my heartbeat so monitors keep going off. And making me feel nervous
- Very uncomfortable for me. Constant readjustments were distressing and Annoying
- Untrained or opinionated midwives/doctors could overreact to the data causing unnecessary stress.
- Adds a bit of stress when it loses a trace for example
- Slightly less mobility, increased anxiety when issues with machine
- Movement restriction, psych involvement increasing risk of assistance in birth, cascade of intervention, limit to upright birth etc
- Difficult if you want active labour - my monitors kept slipping and losing baby's heart rate and that was really annoying as the midwife would have to fix it over contractions, not being able to have a water birth
- Movement and ability to cope with pain/contractions
- Restricted my movement as kept slipping, midwife had to hold it to my abdomen during contractions when I was transitioning and I found that very uncomfortable
- Tricky to get reading and the wires in the right position. Just seems to add more stress to an already life changing time!
- I could not move around or do anything I had been taught to do to manage pain, leading to eventually begging for the epidural even though I had wanted a drug free birth. The epidural is what likely led to needing a forceps assisted birth.
- I didn’t have any but I guess it depends on how monitoring impacts movement and labouring focus as well as how the readings are being interpreted
- Limited mobility, perhaps midwives/dr quick for intervention
- Continuous monitoring was uncomfortable and very irritating
- Not being able to move freely and being restricted

- Not being informed for how often I was having results read, or clear information what it meant (waited for OB to deliver monitoring outcomes vs the midwife/ nurse).
- Unnatural. Being unable to move in the way that is comfortable for the birthing mother and potentially stopping her from the birth she wants. Also potentially have medical providers look at the numbers only and not listen to the birthing woman if she says something is wrong.
- Was difficult when using water eg shower.
- Not being able to move, normal drops in heart rate can impact mothers concentration. monitoring with wires impacts mothers poorly when in labour
- Meant I couldn't move around or have a shower
- Inability to move or use water / bath for pain relief. Sometimes it got out of position and it could seem like baby's heartbeat wasn't ok.
- Uncomfortable, the sound can exacerbate some of the trauma that is experienced with an emergency c section
- Limit movement, stress on mother
- None. I was only monitored once I had the epidural so couldn't have moved anyway
- It can create stress and tension which can slow down or stall labour. It can make the birth team rely more on the "data" than on the mother and what her body is telling her. It can make a mother uncomfortable and not able to move freely which inhibits active birth.
- I couldn't move much and it increased my anxiety hearing the heart rate drop
- With wired monitoring the biggest downside is mobility. Also as previously said, the stress that can occur when you can see a contraction is about to occur.
- My baby has a scar from the monitor on her head.
- Not comfortable
- Possibly quicker to intervene
- Monitoring can show distress when baby is ok
- I was restricted from movement however this wasn't just because pf monitoring but because i had an epidural and was connected to a drip and catheter
- Mother can't move. Monitoring not reliable
- Constantly losing the heartbeat when you move can make you anxious that there's a problem with the baby. Also it's a disincentive to move around. I have up on bouncing on a Swiss ball because it wasn't possible with the monitoring. For one birth I opted for a monitor to be placed on the baby's head because the wireless monitor needed constant readjustment, which meant I couldn't use a bath for pain relief. For my third birth I just lay in bed using the gas a lot because moving around was so frustrating.
- lack of flexibility to move around. Providing anxiety to the mother when the monitoring is inaccurate (mother's movement etc. affecting the monitoring)
- I had an epidural so I already couldn't move around but without the epidural the wires on the monitor would have been annoying and limiting. Also you can't be in the water with them.
- Pain and very limited and stress increased. Reliance on the monitors by staff rather than checking
- Not able to move around - but maybe I couldn't anyway due to induction

- If wireless continuous monitoring is not available you can be limited in movement and soothing methods (etc shower)
- Hard to use in the shower
- Uncomfortable and can restrict movement when you have to try and stay in the same position to keep the monitor on baby’s heart.
- Limited opportunity to move around, water birth
- Focusing too much and get scared at every little variation.
- I was unable to move around and that had been my plan before labour began
- Feeling like you can't move, the sounds it makes
- Focusing on numbers, removing from feelings in labour
- I feel like I had unnecessary intervention (augmented) because of the CFM.
- Can’t move around, painful pushing on belli
- Less range of movement
- No mobility, baby’s heart rate can fluctuate naturally during birth without it being an issue. Monitoring and c sections have increased but stillbirths have not been prevented. Leads to greater interventions than necessary.
- Increased interventions
- Can make you focus too much on the CTG listening to it.
- Causes unnecessary worry. Can be misinterpreted
- Increased risk of unnecessary interventions, stress, restrictions on movement which can increase pain and interventions.
- Extra stress, restricted movement, unnecessary and annoying focus of constantly holding in the right place. Humans can build rockets to the moon, I’m certain we can invent a better way to monitor a babies heart beat. If it were men who gave birth this would already exist
- I hated the belt thing and found it really restrictive once I was in the final stages. I just tore it off and no-one seemed to care though
- Some staff (mainly OB, not the midwives) seemed obsessed with the trace and the numbers and would forget to include me, the person actually giving birth, in the conversation about how my labour was progressing and how my baby was doing. This was especially frustrating when I specifically asked staff not to whisper about numbers as it made me more anxious.
- The internal monitor hurt more than giving birth and I wasn’t aware of any risks to my baby prior to insertion
- Can cause anxiety, don’t have freedom of movement as much.
- Reducing movement during birth if on static monitor. Disrupt natural flow of labour
- Not being able to leave the bed
- For my experience of intermittent auscultation there were no down sides. The midwife worked around me and my movements and position changes. If there had been issues with her heart rate which led to intervention my answer would likely be different.
- Restricted movement, distraction from strategies - not an issue for me though
- No down sides.
- My wireless monitor kept cutting out so eventually I needed to be hooked up to the wires- this occurred in both of my labours

- There were no down sides for me, but perhaps someone who is highly anxious may find it more distressing than reassuring
- Restriction of movement, not being able to use bath/shower.
- You can't move much
- Rush to intervene when babies heart is recovering
- Can't move around as needed, and baby had a cut on his head from the monitoring clip
- That it's not always accurate, and any little dip or rise in heart rate caused everyone to try and take unnecessary action
- I didn't feel I could move and when I was pushing, a midwife was holding the monitor the whole time it was really uncomfortable and hard to get into a comfortable position to push

- Unable to use water (found that great in my first labour) - the stress when loss of contact occurs. Cascade of interventions
- It's hard to move, invasive and isn't presented as a choice
- Often used when not necessarily needed but just used because data is wanted for health professionals
- Restricted movement
- It's interfering, takes focus away from concentrating on contractions, causes unnecessary concern when the trace isn't picking up correctly. For CTG there is no benefit to morbidity or mortality of mother or baby, but an increase in c-section risk, which is a massive deal. Hospital staff downplay the risks and recovery time of c-sections all the time and it has to stop.
- Uncomfortable and the shower messed with the external monitoring meaning they had to change to internal
- You can be a bit stuck with movement if you are all wired up
- Not being able to walk around make labour much harder
- If I couldn't have been monitored wirelessly and had to stay on the bed I would not have allowed it because I feel it was likely slow labour down
- I wasn't able to have wireless as they forgot so I wasn't able to move around easily
- I had wired, and they refused to take them off
- Not necessary if low risk
- Uncomfortable, restricted movement, lots of people touching me to get a good trace which was distracting, wasn't allowed to shower, led to looking for problems with baby that led to cs
- Mobility, interruption of birth environment
- Unnecessary intervention
- Restricted
- Can't move freely, use the shower, more likely to end in an unwanted c section.
- Having to stay in the room for monitoring
- Straps are uncomfortable
- No
- Couldn't use some of my methods of pain management - ie TENs machine, and had some mobility but not quite as much as I would have liked.

- For me there was none. My midwife was respectful and it felt non-invasive and I was happy to hear the Doppler
- Intrusive and technical issues. Unable to get in the bath
- It’s not always accurate and can increase unnecessary intervention
- I didn’t like anything touching me. Monitoring pads kept moving out of place
- Not being able to move as freely as I would have liked.
- couldn't move when initial monitoring occurred. this was very uncomfortable
- None
- It was restrictive. And once it went on there was no bath/showers.
- The effects on the baby
- Oh
- Them slipping and not picking up the babies heartbeat
- Not being told the risks such as the clip causing an infection in my baby’s scalp. Very stressful being told the monitor kept cutting out when I was sitting down on the toilet- was told I had to stand up in order for the heart rate to be detected. Not comfortable at all. Controls the labour and process / progress due to the constant stress and not being able to move freely around or comfortably.
- Too much information leads to unnecessary interventions
- The straps slipped and had to be adjusted to pick up the babies heart beat
- It was hard to change position without the monitors falling off or the midwife having to hold them in place during contractions
- Limited options for movement and positioning. Focusing on the readings. Feeling unsafe in the labour
- I don’t know
- Unable to move around
- Inability to move when monitored with wired CTG, wireless CTG appeared unreliable with tracings when moving
- It was forever moving and having to refind babies heartbeat, it dug in and was uncomfortable, I couldn't move around easily, and could get into water. My partner had to hold monitor in place which was a pain
- Caused a lot of distress in me wich of course caused more distressed in baby. I wasn't allowed in the water anymore so i had a more rough time with pain
- Can’t move around freely.
- Position i was allowed to be in
- OBs determining there are decelerations which show baby is in distress when in fact they are not. And then the diagnosis of baby in distress leads to further interventions which aren’t actually required.
- Continuous monitoring often gives false alarms, it has not reduced negative outcomes for babies however has increased the c-section rate. Often Hospitals do not have wireless monitoring which restricts movement, which in turn can cause further issues in labour.
- lack of freedom to move; midwives constantly looking at the monitor and not at you; getting a whole heap of data but that data not translating to a better outcome
- Unable to fulfill other birthing wishes as very restrictive
- Leads to more intervention, restrictive and reduces the natural aspect of childbirth
- I imagine continuous monitoring could be restrictive, which might slow labour

- Straps, continuous interruption to labour process inc having to move position if FHR can’t be heard, if CTG staff making decisions based on personal interpretation
- Limiting position and location
- I couldn’t move much, this made it very difficult.
- Even without wires my movement was somewhat restricted to positions
- Less freedom of movement
- Reduced movement, inability to have water birth, additional noises, reduced normal labour experience as always checking the CTG trace. Even with wireless monitoring the censors often moved on my stomach and needed frequent repositioning
- Inaccurate, rushed, monitors always having to be rearranged, increased vaginal exams, potentially unnecessary caesarean
- Limited movement for mother, discomfort. Feeling of birth being clinical
- Mother being able to hear it as well
- Unable to freely move around, took a while to hook up
- Being connected to a machine
- I didn’t move around and was staring at the machine over analysing for 15 hours
- It is comfortable, it medicalises a lot of labours that don’t need it, it causes a level of stress and anxiety that prevent the women from relaxing and allowing labour to progress.
- More ammunition for a c section
- Movement restriction
- Lack of freedom to movement man’s leads to unneeded interventions
- For me none as i never labourer. However I can imagine the wires would be constrictive during labour and frustrating as it would impede ability to move around
- For continuous; if movement is restricted; if you can't go in water; if there is intervention because baby's heart rate drops but it is actually in a normal range and intervention is unnecessary. Other ways that monitoring could be invasive and disrupt calm oxytocin encouraging environment
- Slightly annoying to keep readjusting it
- Restrictive and a source of anxiety for mums if we aren’t properly informed on how it works and what is normal/abnormal
- Interrupting your focus on labour
- Can’t move, feel restricted, staff more interested in the monitor rather than the patient
- False sense of security, attributing abnormalities to equipment rather than progression of birth
- Can make a mother MORE anxious, some medical providers lean to heavily on it and base all decisions around it which can then lead to poorer outcomes for the mother in terms of the type of birth she was aiming for
- Uncomfortable
- Being confined to the bed SUCKED big time. My baby also kept kicking the monitors, so they had to keep being repositioned.
- Interrupt labor, loss of connection to body/birth, stress for bub
- Slowed down labour and made me get an epidural as I couldn’t move off the bed because of the constant monitoring
- Pain relief and movement options limited.

- Increased intervention, less able to move around
- Mother very uncomfortable and felt restricted
- Don’t know if any
- Restricted movement, increased risk of intervention, injury, caesarean and obstetric violence.
- Not being able to move around. Hypervigilance when trying to relax
- Uncomfortable, kept slipping and beeping, ended up with the screw in his head monitor
- Not being able to move freely. Panic when monitors moved slipped off during last stages of labour
- PAIN. Anxiety associated with alarms and watching rates/contractions
- Not being able to move around freely - I had tens machine cords, drip, monitoring wires so it was very difficult to move or go to the toilet
- For me it was restrictive. I wanted to use movement to help my body and baby progress but had medical intervention forced on me instead
- I couldn’t move to manage my pain so ended up needing gas earlier than I hoped
- Due to wires no movement around
- Cascade of intervention. Reduced mobility.
- Anxiety about the noises and not knowing what’s going on
- Just a little cumbersome at times to navigate around with the wires.
- Could lead to unnecessary intervention. It is also distracting for myself as a birth mum.
- Restrictive, annoying, increased risk of intervention
- I was unable to move freely, be upright and walk around, unable to shower or take a bath
- Would lose baby when I moved. Batteries ran out
- Restricted movement even if wireless as sensors do not always detect baby.
- Restricted in movement and ways of coping with labour
- Lack of mobility when you are on a wired machine
- Lack of movement, monitor was falling off - couldn’t access water due to this.
- Too much information can make people stress without need
- I felt tied to the bed like I couldn't get off it, I felt very restricted in choices of labour positions, it contributed to my re-traumatisation from previous sexual assault.
- Restrain movement, increase fear
- If I was connected up to a monitor it would have impacted my ability to move around or maybe go in the bath and that would have been a problem for me. Also with the monitoring I did have with a Doppler it did occasionally interfere with my focus during contractions which I found difficult sometimes
- Rescued movement and added stress when any beeps or noises happen that aren’t familiar. Hard not to focus on the monitor and that’s it during labour
- Comfort and freedom
- Hard to get in a comfy position
- Possibly less free movement and optimal positions or use of water
- No movement, constant downplaying of what’s going on for the mother, no trust given to maternal instincts
- I didn’t see bay down sides to being monitored

- Risk of intervention
- Panicking when the beeps and noises would change even though baby was ok
- Less movement. Midwife relying on CTG reading instead of hands on in regards to contractions
- Not accurate, can stress over bad read/misread
- Reduced mobility, increased interventions, increased disruptions to readjust monitors
- Restricted movement and the midwife constantly needing to readjust it.
- It is uncomfortable, the midwives become a bit obsessed with it, it can add to the anxiety of giving birth, you cannot move around when you are being monitored, the midwife would get frustrated if I moved to deal with a contraction because she was trying to get a heartrate for the baby and that was the most important thing.
- Restriction in movement (if wired or uncomfortable)
- Concerning when bubs heart rate changes and no one discusses it with you
- None for me personally. Sometimes finding a good trace can be challenging
- Higher rates of c section for healthy babies
- Complacency from providers (not my experience)
- Less freedom to move around, I wasn’t able to have a water birth as I had hoped to do so
- Limited movement, cascade of interventions, lack of statistically significant data that says it improves birth outcomes
- Restricted in movement and positions as the sensors shift around and don’t pick up babys heart beat effectively
- Not able to move, care team more concerned with monitor numbers rather than actual patient and baby
- I felt with wired monitors, I wasn't able to be as mobile as I would have liked during my labour
- Difficult to move / change positions, using the toilet was difficult as the monitors would flip over and lose the heartbeat trace and when this happened the alarm would sound
- Restrictions on movement, use of water. False negatives, creates more fear, more centrally monitored
- Likely of doctors progressing to C-section discussion
- Limited movement
- Lying in bed with no real coping mechanism
- Being bed bound, uncomfortable and stressed during labour. Leading to other interventions such as epidurals to manage pain that you can’t manage in other ways
- I feel it gave the medical staff a false sense of security that she was doing better than she was. AGPAR was 2 at birth. They should have taken her out earlier
- Unable to move, strapped to a bed, unable to even go to the toilet easily.
- Restriction in movement on the bed
- Increased rates of caesareans
- Too much information may lead to early intervention
- Limited mobility. Medicalisation of labour. Another way that medical staff pressure Intervention onto a birthing woman. Inhibits the woman from being listened to and

understood- why listen to a birthing woman's desires and instincts when you can try on a little machine?

- Over zealous intervention
- It was quite uncomfortable and was malfunctioning which added an extra layer of stress to an already high pressure situation
- N/a
- Restricted movement, restricted access to pain relief (water), making the birth space feel less natural, constant beeping, added stress of worrying what the noises mean
- Limited movement, time which forced to be in 1 position, made me feel anxious that something was wrong
- Over-reaction, movement limitation, fear, interruption of physiological birth
- Not being able to move around much
- Interruption of mental state, lack of flow to get in the zone to labour/ birth, uncomfortable, constant readjustment of monitor, concern when you hear the machine beeps, or if the beeps change.
- None
- Can't move around as much and uncomfortable
- Less freedom to move around, use water as a coping mechanism etc
- None really. Being wired could cause a downside, but the clip on baby's had and being wireless is painless, probably the best method when considering constraints and things attached to you
- Cords
- Increased interventions, lack of control over birthing, birth trauma for mum and bub
- Movement of linked to CTG
- Left a sore and scar on my sons head. The monitoring confirmed the need for an emergency c section but I think that was a result of being pumped too quickly full of induction hormones, so if anything the monitoring saved my boy and I who both had a decrease in our heart rates
- No being mobile and possibly led to my baby not being in correct position for birth
- If wanting to move around the straps and cords could be restrictive. This was not an issue for me as I had an epidural.
- It moved out of place a lot
- None
- Restricted movement, uneducated of unusual sounds, restricted movement in shower wasn't allowed to splash machine, nurse fixed monitor first rather than checking up me first
- Mainly the mobility issues if wireless machines are not available
- I couldn't move freely around the room/change positions, I ended up needing an emergency c-section
- Lack of movement
- Unable to move, feel if you get off bed or go to toilet you are an inconvenience
- Impeding movement during labour, which helps progress labour and can ease delivery, preventing need for caesarean
- Restricts movement for mum. Can become a focus for the mum instead of being focused on labour.

- It was very distressing for me. It also wasn't explained at all that some dips in the baby's heart rate are normal/expected
- Limitation on mobility during labour
- They jump in too quickly to go for a c sect, hard to move around with wires
- Bed position/postures needed, may incidentally reduce mothers capacity to, comfort or perceived ability to move around and adopt labour positions that suit them.
- It was uncomfortable and impossible to move around
- Pain. Midwives so worried about a beeping monitor they don't even know the sex of your baby when it comes out.
- Interruption of the birth space, adds to the vulnerability of the mother having strangers touching or near her, distracting. Along these things may have an impact on the progression of the labour and consequently the birth and the babies health:
- Lack of movement/freedom
- Couldn't get up off the bed for the whole labour
- Early interventions
- Higher potential for emergency c section
- Uncomfortable and unnecessary for Mum or Bub
- I couldn't move and get comfortable. Luckily it was only while getting checked over before moving to birth suite
- Difficulty in labour
- Restricted movement, restricted birth positions
- None
- If I didn't have an epidural probably pain and movement.
- Without a medical background, I believe the continual monitoring created a 'red flag' for my medical providers that drove decision making through the labour in a way that made me feel stressed at the time
- I was tied to a bed and unable to move/escape, it contributed significantly to being re-traumatised from previous sexual abuse, i believe it hindered my ability to give birth unassisted because i was limited in my choice of labour positions
- I had to change positions to get a good heart rate reading and because I was in the water and unable to get a good heart rate trace I was made to get out of the bath to continue to give birth

3. **If given the choice, would you choose the same form of monitoring?**

- It would depend on what happens in labour and if I felt like I needed the reassurance
- I would choose less monitoring
- I'd want more intermittent monitoring, not continuous
- I felt restricted being monitored
- Smaller/more mobile monitoring would be preferable.
- It restricted me too much
- I felt it was the least invasive and most reassuring for everyone.
- For my second I chose to have no monitoring (freebirth) but I was 39 weeks so I feel like the situation was different. I was happy with intermittent monitoring for my premature baby

- I have more education now
- Intermittent Doppler would have been adequate due to light mec. I'm much more informed now
- Seems like a relatively small concession (only when wireless is available!) to keep caregivers "onside". I feel like there are more important things to stand my ground on and I can deal with the monitoring in order to appease them
- Hope for intermittent monitoring in future
- No, I would choose something different - my birth experience was very negative and monitoring contributed, especially by taking clinician attention away from me and by restricting movement and pain relief options (eg water)
- It was very restrictive
- I wouldn’t want to have an epidural without monitoring
- No freedom of movement
- I would only agree to fetal scalp monitoring and not as a matter of course.
- Wireless monitoring. More movement. No fetal clip on head. I wasn’t allowed water immersion due to this.
- My son has 2 scars on his head where the scalp clip was inserted incorrectly. Every time I look at it, it reminds me of the trauma we went through. Nobody monitored our CYG and it was syncing with the baby next door without anyone realising. I would request doppler/no monitoring for my next pregnancy
- After my experience with birth I will more than likely elect a Caesar next time so I will likely just have an admission CTG
- If it was another preterm labour - yes same again but maybe wireless if available.
- Only intermittent I’m not going through having a CTG again.
- I would decline it completely and get in the bath
- If I need to be continuously monitored wireless seems like the better option
- Depends on clinical risk factors
- Monitoring resulted in being restricted to bed, and requiring an epidural. Due to pre-existing hip problem, I needed to be able to move to manage pain and I was unable to do that. Ended up with epidural and slight shoulder dystocia in baby
- Don’t want to attach anything to baby's scalp next time
- pinard monitoring (homebirth)
- In three births I have only ever agreed to intermittent handheld Doppler and would do so again as the most non-invasive method and because of the evidence that it is less likely to result in unnecessary intervention due to clinicians misinterpreting foetal condition. I feel like on a continuous trace every tiny little fluctuation in HR is turned into foetal distress and intervention brought about unnecessarily. Not to mention CTG cords and machines and belts etc being cumbersome and invasive and restrictive, the idea of being hooked up like that gives me claustrophobia!
- So that I could have got in the bath or at least the shower
- I would like a lot more information to be provided about it and to not just have straps on my belly after barely even asking
- I would love a waterbirth again, I liked the Doppler but it also was annoying sometimes but maybe I was just an irritable birthing woman
- I would have prefer just the intermittent only

- I won’t go near a hospital if I can help it. Check every 15-20mins if you must but I’ll fight against continuous monitoring
- To reduce cascade of interventions
- I would be more persistent about using wireless monitoring
- I have a lot more understanding around monitoring and don’t believe constant monitoring is necessary
- Would take advice of health professionals, but would also like to keep moving and active
- It made me more anxious and physically uncomfortable
- I would have preferred wireless but I don’t think I would have been comfortable with just intermittent in my situation
- I would want to move around freely
- Disrupted the flow of labour
- Intermittent monitoring as I know that there is no evidence that continues monitoring reduces fetal or maternal complications.
- I'm not allowing any type of monitoring again unless I request it myself
- I’d avoid the continual monitoring
- I only agreed to CTG because we were an overdue, induced VBAC. With less risks I would have had intermittent monitoring
- Wireless so I could still move around
- I’d still want a CTG within the context but would much prefer the stick on, strapless option
- I was only checked with the Doppler a few times which allowed me to move freely and feel like I could trust my body. The Doppler was a quick reassurance that all was well and I should continue to trust my instincts and continue as I had been. This was a part of my plan for minimal intervention.
- Intermittent or none at all if possible - if there’s no reason to believe baby’s in distress there’s no reason to monitor!! The limitations of the CTG to detect heart rate of my posterior bub had a serious impact on my ability to labour effectively.
- Induction with continuous monitoring ruined my birth experience and I would never choose it again. If needed I would request intermittent monitoring with a wireless only IF necessary
- Hand held doppler works just as well as any sort of monitoring.
- Cordless would have been great as my whole labour I was active
- That’s what’s available at the hospital to me. If bub needs monitoring- I’ll take it
- I would want more frequent monitoring considering my birth outcome and baby’s outcome
- Yes, I decline the continuous monitoring and that was a great decision. I still wasn't enjoying the Doppler monitoring but I understand the value it has.
- I would choose to have the midwife listen instead of CTG, just because it was a bit annoying
- Because first baby went into sudden critical distress with no warning or symptoms, CTG only thing that picked it up.
- If being induced or there was another reason I felt warranted a CTG; I would also be comfortable choosing only intermittent Doppler if that felt safest/appropriate to me

- It resulted in the care providers restricting my movement entirely and even putting their hands on me to force me back into the bed when I tried to move, they used it as reasons to continually tell me they needed to intervene which when reviewed in hindsight was not true or indicative of fetal distress, it was also incredibly distressing to feel the need to move but be forced onto a bed and encouraged to get an epidural and then not listened to when the epidural wasn't working because that was used as another reason I couldn't move despite it not being effective. I don't think I would have required the epidural had I been able to be upright and in the shower like I was earlier in the labour so I believe it led directly to the way the birth outcome
- Because the midwives did it in such a way that was unobtrusive, not painful and reassuring.
- Only Doppler and only occasionally. I did not need a monitor to tell me I was in labour, it distrusted my hormones and causes discomfort for lying still
- I would decline constant monitoring with straps and choose intermittent monitoring with doppler
- Wireless with straps but kept losing baby's heartbeat whilst on fit ball and kneeling in shower
- I would opt for occasional heart rate monitoring with a. Doppler
- I want choice and control. I want to know the downsides before I agree. I want to know the alternatives the best part of the labour was when the older nurse took the monitor off briefly to give me a. Break
- Probably ask for a better wireless or Doppler to be able to bath and move around more
- Depends on why they recommended it.
- Natural labour, intermittent monitoring by the midwife was reassuring for me, my husband and the midwives at the birth.
- Doppler only. I feel continuous monitoring greatly impacts the cascade of intervention
- If anything- a Doppler every now and again to reassure the midwife that everything was fine
- I feel like it truly impacted my labour and birth to flow naturally with how I wanted to move my body and change positions. I really wanted to sit in the pool but was told I couldn't because of the monitoring. I was constantly worried about bumping the straps which kept sliding off anyway. I feel sad thinking about it.
- I didn't have any monitoring for my third
- I would have no monitoring.
- I chose a different method in my second labour (skull clip, water had broken in both labours) and it allowed more free movement and less interruption, meaning a more self managed and focused labour
- I know more, would have a different setting, more hands off, use of water, more movement
- I probably won't have another baby though due to my age!
- For my second baby I was more informed and I actually declined constant monitoring which was against hospital policy.

- I’m choosing an entirely different model of care (privately funded homebirth) and so continuous monitoring is not an option, and I don’t want it anyway.
- If I didn’t need constant monitoring, that would be fine with me.
- I believe the CTG monitoring was a major contributor for my failure to progress which lead to a caesarean
- Only under same circumstances. I will be trying for VBAC so will choose continuous monitoring
- I believe the wired monitoring reduced my movement and slowed my labour down when I arrived at hospital. I also believe concerns about my wireless monitoring, rather than observations off or listening to me - contributed to my instrumental forceps delivery and took away my ability to birth unassisted.
- I now know the effect it had on my baby
- It severely impacted my confidence, calm and comfort
- I believe Doppler is quite efficient in monitoring intermittently but I would also ask for the right to refuse at points if I’m not wanting to be interfered with.
- The same or less, I had the encouraged least restrictive I would have this again, or if it was safe I’d have none
- I would like to be able to move freely and walk out of the birth suite if I wanted to
- I'd like to try wireless CTG
- I would want something different but know the policy doesn’t allow this. I am not confident/brave enough to go against the policy.
- Or none if easier labour. Why because it gave me and health care peace of mind. Because despite a long labour (40+hrs active labour) we could see that bub was fine. When readings started to be off and they wanted to interview I asked for scalp monitoring and we could again see she was fine.
- I felt well supported with this kind of monitoring and it gave me reassurance. The midwife could easily monitor with the Doppler in whatever position I decided to assume, I was not restricted
- I will not be induced again and will opt for intermittent Doppler monitoring
- It completely depends on the circumstances of the labour. I would be more protective of my spontaneous labour so that more invasive monitoring was not required.
- Uncomfortable and restrictive
- Wireless so I could move around. I had an epidural because I felt there was no other way to manage the pain. I didn’t need this with my second birth when I was free to move and change positions
- May decline monitoring all together
- To allow me freedom of movement and pain relief (water).
- I’d ask not to be monitored while labouring unless I asked the midwife to check
- I would want wireless or intermittent with a Doppler (I had with my son in 2014 and was a much nicer/freer experience)
- So uncomfortable and irritating
- I know it was indicated but I do wish I didn’t need the monitoring.
- I would simply choose intermittent monitoring for reasons previously stated
- Because I would likely be attending a VBA3C in hospital- I wouldn’t have a choice- I’d be forced into continuous monitoring again

- Maybe wireless could be more comfortable so don't need to worry about the cables moving the pads etc
- Having some form of monitoring allowed me to have a no intervention birth because I used it as a tool to decline induction, pain relief etc
- So I could use water as a form or pain relief, and move into any position that I felt I needed to. Plus my baby seems annoyed by it and seem to push against it during an antenatal visit which caused her heart rate to race at points. It was very uncomfortable.
- I didn't like that there was a screw in my baby's skull. I didn't realise the SIZE of the device and the possible implications on brain activity etc. (there are probably no studies on this either!) I would have preferred a home birth for this reason.
- I prefer low-key, low-intervention labour, with the attending midwife being relatively "hands off". Continuity of care is so important here, as an established relationship prior to labour means that a birthing woman's preferences are known and respected.
- It really depends why. I think in my case with fetal distress it was important and I still had a vagina birth with no forceps, vacuum or episiotomy.
- Would probably happily choose no monitoring, but depends on the midwife presence I guess. I'm not anti so if she feels she would like to do so then I'm happy with very intermittent hand held monitoring.
- My next labour I want only intermittent, so I have a lot more freedom of movement and the ability to use water during labour
- I'd like to be able to move around and not feel so observed but unsupported
- I am home birthing next time, there is no way I will go back to a hospital
- Intrusive and made to feel like a prisoner. Not sure if it was my body pushing out a baby or the midwives
- I now know there is no evidence for continuous fetal monitoring and so I would try to avoid it.
- I would pay for a private midwife who knew my name and my risk preferences
- I'd like to try wireless CTG if it were available
- I would trust my body more. I knew my baby was fine
- Would like wireless of I have to have it again
- I would not allow it to be done the way it was done, I would prefer not to have my baby than go through that rape again
- If there's wireless that's better for mobility
- I want a home birth next time to avoid hospital red tape/policies/overbearing OBs and will accept intermittent monitoring if needed. I found being strapped up restrictive and difficult to move into positions without moving the CTG. I would not consent to internal monitoring at all.
- Intermittent monitoring only & this what I did with my second baby. In fact it was the only option available in that birth setting
- Wireless would be better.
- I was able to be sitting on the ground to bed
- It didn't bother me having these types of monitoring. Had it restricted having a water birth then it would have bothered me

- Although that stick on one looks better. Maybe the toggles wouldn’t move all the timeline they do in the belts
- Wireless monitoring sounds much more comfortable
- I would want periodic monitoring but not constant monitoring. This would aid freedom of movement and reduce the focus on the tech, allowing the midwife to support me.
- If it was for being induced again I couldn't remember it impacting me when compared to IV drip
- I’d definitely want wireless as an option and be sure the batteries are fully charged!
- I wanted a water birth
- I would never have continuous monitoring again and issues including monitoring are a big reason I never want to give birth in a hospital again
- It seems to be the best option- gives reassurance that baby is ok, can still move around somewhat
- Didn’t know there was wireless options, definitely would prefer this
- This kind of monitoring was really restrictive and intrusive for me. I wish I had more medical knowledge to have potentially made an educated decision to refuse.
- I haven't read enough about risks of Doppler use to say I would use it again.
- I would prefer my consent to be given, rather than have no choice
- Intermittent doppler is fine but continuous CTG not
- I felt that it inhibited my labour and movement because I was strapped to the machine. I would’ve liked to get in the shower or tried different positions
- I would never birth in a hospital again. Sadly, I already was against fetal monitoring for pregnancy and birth but due to hospital policy and my birth derailing I had to experience all forms of it and ultimately ended in having surgery instead of birthing my baby
- I only consented due to the complication of prolonged rupture of membranes. If where is no medical risk then I would not consent to monitoring
- Given the option I would want wireless stick pad monitoring to give the best chance of staying mobile
- Minimal monitoring to be able to move freely and connect with your own body
- I had a fetal scalp electrode, it was very distracting and as they had to adjust it a couple of times it really threw me out of my headspace having to get back on the bed for another internal
- I would push for intermittent so that I could be more active during labor & move my body to cope with contractions
- I want to trust in my instincts. I would choose wireless intermittent monitoring only
- Wireless would be good as I will be moving more during my next labour
- I would choose no routine/regular monitoring throughout labour
- Again it was annoying and distracting. Doppler checks every now and again would have been better
- Something sticks a little better and allows me to move a little more.
- I would want wireless monitoring if possible next time, but would be more than willing to have whatever monitoring they recommended
- I would decline CTG monitoring and only consent to intermittent Doppler readings.

- I would want either wireless or handheld methods so I can move around instead of being restricted to a bed.
- It depends on how the labour is going. If I need it, it's fine, but prefer to go without
- Peace of mind
- Wireless, but still continuous
- I had intended to have a more active birth for my second child and made preparations towards that end including discussing when monitoring was needed and could it not be on constantly. Unfortunately, my second baby was breech and labor was considered dangerous due to positioning and a csection (without labouring) was necessary
- Myself and my baby had been labouring for so long, I don't think that monitoring was completely useless in my case as it helped determine that baby was not coping. We ended up needing fetal scalp monitoring while baby was in the birth canal.
- I was afraid it impeded my labour and I hated my midwives. They didn't care about me at all.
- Not applicable
- Head probe I would choose again as it enabled me to move. CTG straps were annoying
- No, I hated the restrictions
- I would not have chosen to be induced if constant monitoring was told to me
- It would depend upon the situation and reason for monitoring
- I wouldnorefer wireless
- I would be willing to have anything that doesn't inhibit movement and water use. I would just like proper knowledge
- Want wireless monitors
- I would want wireless monitoring or intermittent monitoring. I would also delay continuous monitoring until I was in established labour.
- Second birth was precipitous unplanned free birth. Probably would have opted for monitoring or at least not objected to it if hospital birth
- While I found it stressful I also feel like it wouldn’t be offered if it didn’t serve a purpose. If something bad happened and I had turned down the use of monitoring I would feel responsible
- I would prefer wireless so it’s easier to move around
- Depending on the situation and my health and our babies health
- Unable to move
- I would like to be able to move around, be able to focus on a positive birth experience
- If there aren't the same reasons (meconium in the water) I definitely wouldn't opt for continuous monitoring as I found it stressful, however of there is a medical reason it is recommended I would most likely do what I'm told by the midwives
- Wireless would be better. The machine did not work when I was admitted
- I would have liked to have been able to stay active with monitoring.
- Allowed us to learn when baby was in distress, could have ended worse if information not known
- I would want less monitoring. More focus on me and my body and help to move through labour

- My focus was on keeping bub safe and didn't mind how that happened
- It wasn’t explained to me and I have no idea what it really is, I only briefly remember having my nany monitoring during my labour.
- Didn't know if options were available
- I liked knowing that my babies were safe. At no time did I feel that the monitoring was affected me. In saying that I don't really know what my other options are
- Depends on if it was needed or wanted at the time. Very subjective to the pregnancy and labour
- Wireless monitoring for freedom of movement
- Wireless ECG would be great. Water proof one even better
- No need to be monitored. Not high risk. 2nd pregnancy was a water birth and was only checked a handful of times last labour was checked between every contraction which was so difficult when I was getting them every minute and sometimes would take the student 3 contractions to find it or give up ðŸ¤¦â€�â™€ï¸�
- Private midwife and home birth, hands off monitoring.
- Wireless! So that I could move around the room. Without midwife adjusting constantly
- I would ask for wireless or intermittent monitoring grom the start so I am able to move freely around.
- Monitoring with stick on bits would have worked so much better for me. It would have meant I would have had a bit more flexibility to move around more.
- Wireless monitoring was the best for continuous monitoring, but I’d possibly choose intermittent monitoring if it was a low risk labour so as to have more freedom of movement
- I would opt for a repeat c section to avoid needing the monitor placed on my baby’s head
- Due to the issues with my sons birth If I was to have a VBAC I would want constant monitoring as I feel I would be paranoid about a loss of heart rate again
- I would want wireless if necessary
- I would prefer wireless if monitoring is absolutely necessary
- If it were available the sticky patch monitoring would be ideal. The ctg monitoring keeps falling off and needs readjustment
- Wireless would be better
- Movement was impeded. I had complications and would not consider vaginal birth again
- I didn't mind it but I would prefer wireless
- No monitoring or Doppler only. CTG impedes the experience and well-being of upright birthing for labouring women
- I would like to be able to move around freely and explore options for positioning without worrying about the CTG falling off or losing a trace.
- Wireless would have been preferable
- I would rather no monitoring if birth is not high risk
- So I could move freely
- I don’t know if I would go wireless or wired.
- I would not choose the CTG wired monitoring. The scalp clip was fine as it allowed me to move and change position but I would have liked to do more research on the

risks to baby with this type of monitoring. For an uncomplicated labour I would choose intermittent monitoring to prevent unnecessary intervention.

- I would see if we could somehow compromise and not have to be monitored so intensely
- I would stay at home to have another baby in hindsight. Happy to have Doppler monitoring but would be happy with only that
- Think it may have been the only option available in our regional hospital
- I would choose intermittent monitoring for a low risk pregnancy. If monitoring was clinically needed for a suspected problem, I would opt for FSE as it would be less restrictive to my movements and less disruptive to my labour
- Depends on the labour and options available to me. We have a small hospital. You take what's available
- I would choose regular Doppler checks instead as it can be done without interfering with my movements and it doesn't make noise that is distracting.
- I'd do what's recommended
- Would opt for wireless as I feel movement is beneficial for labour.
- Hearing the heart rate was reassuring for all involved and as I have epidurals it's easier to be hooked up with wires and straps
- I would not get monitoring at all
- Due to experience of labour opt for C/s only
- It was for the baby not me so I didn't mind
- I did have a legitimate reason to be monitored (suspected cord around baby's neck), so I accept it was somewhat necessary. But it still caused me extra stress/fixation on the heart rate number rather than focusing on breathing and the whole process of labour etc
- Depending on how the birth was going and if I needed medical /drug intervention
- I would ask for least restrictive monitor and explore benefits and disadvantages of it
- I think it hurt my baby
- So I could move around more freely
- Well now I know there is wireless, I would definitely choose that instead. Also would not agree to internal monitoring again, would opt straight for csect at that point
- Wireless so I could walk
- I would prefer to have no monitoring if bub and myself were well and there were no complications
- I would want wireless monitoring again and hope it gave consistent readings
- I had wireless monitoring the next time which was marginally better.
- A different circumstance may require the monitoring. I will always make sensible decisions.
- I don't believe my baby was at risk, I could read the monitors and know that there was no indication of any risk. I wanted an intervention free birth and wasn't allowed it.
- I'd really like to avoid the need for monitoring because of the limitations it out on birth and also the constant slipping during labour was really frustrating
- I would have wanted wireless monitoring so I could manage my labour pain actively.
- I had a difficult birth experience so my second child was born by c-section. If I had more children I'd opt for c-section

- I will ask for less frequent monitoring
- The position was too high, and I requested it to be turned down throughout the day then greatly reducing for active labour so I could feel the â€˜resistance’ to push against. I feel one of the reasons I had pushback to the reduced numbing/epidural was due to the need to be monitored and this was easier for staff. I did not feel this was intuitive to my body and desired birthing experience and was very different to my first birth. I was forced to sleep during my early stages of labour and it was not a mindful or overly connected birth process, though admittedly far less painful. My first was 24 hours and extremely painful and traumatic, however there must be a balance between ‘toughing it out’ and lay in bed and sleep in a semi drugged state’. I would have liked to be able to move and be involved.
- I want an intervention free birth with the ability to get into any position I want and use water as pain relief, not be restricted by continuous monitoring.
- Would want wireless.
- If pregnant again I would likely choose epidural again so wired monitoring would not be a concern or problem
- The wired monitoring made me immobile.
- My babies were delivered safely and were not distressed at any time. Because I knew they were comfortable I was able to focus on pushing and not stress about any issues for them. Also during the delivery, Twin A heart rate would slow down everytime I would push. The monitor meant that my Dr knew this very quickly and intervened with the vacuum to get her out safely.
- Being monitored is highly frustrating, but I'd hate it if something happened to my baby and it was missed because of a choice I made
- I would want no monitoring or wireless monitoring
- With an epidural yes because I can’t move anyway. But with no epidural I’d rather not.
- I’d prefer to move around and would refuse induction next time
- I did not know wireless was an option and this may have been less cumbersome
- Wireless or not continuous so I could walk around
- If no distress, I'd want intermittent monitoring of some type
- I would want intermittent monitoring unless clinically indicated.
- Wireless would be better
- I suffered unnecessary interventions without consent due to use of CTG, which is known to increase interventions without improving outcomes. If I have a midwife present at a future birth I may consent to intermittent monitoring only if I feel it's worthwhile in the moment, otherwise I will just trust my body and baby and follow my intuition.
- I would insist to have Doppler checks rather than CTG to have more freedom of movement and to be able to get into my birthing head zone without the constant stress of thinking about losing the signal of the CTG
- I would find out more ahead of time
- Now that I know there are wireless monitors, I would choose this option.
- I wish I had wireless monitoring that just worked for me so I didn’t have to have the probe attached.
- I would love to be able to freely move and use other methods of pain relief

- It was non invasive and non restrictive and provided reassurance
- Wireless monitoring gave me the freedom to move around, bounce on my ball, have a shower all while knowing the baby was safe
- I had no issue with being monitored with the CTG + wires as after the epidural I was essentially bed-bound anyway
- Would depend on the pregnancy. My son was born with a disability so I'm probably more like to go with more intervention not less. However it definitely had an impact on how my labour went, it was not as I had hoped.
- I am currently pregnant and have elected for intermittent monitoring by Doppler as I believe constant monitoring led to an unnecessary c section in last birth
- Would want to use water as pain relief
- I'm more aware of my rights
- I'd be open to it but I'd probably opt for no monitoring unless clear indication of distress
- Intermittent Doppler monitoring only, previously agreed upon with a private midwife. No pushing for scalp clip or CTG at time of birth.
- It would depend on the circumstances. I'd prefer to be able to move but if I needed the monitoring for the safety of my baby I would do it.
- I was able to still move around and get into the birth pool so it did not impact on my labour. I barely noticed it was in situ and it allowed for quick detection of my baby's distress b
- I would like wireless
- I would opt for intermittent monitoring or Doppler, for more movement
- Had same for 2nd baby for different reasons. Hated it equally. Won't do it again
- I would want to be able to move and turn lights and sounds off
- I don't want an epidural again
- It was uncomfortable I couldn't move freely about the room OT use the shower which I wanted to be a big part of my birth plan. Constant interruptions whilst in labour negative effect on the labour experience
- I want to be able to move freely and not have something so intrusive
- Depending on my obstetrician's recommendations and assessment of mine and my baby's risk
- If having epidural or intervention yes but for VBAC I will be requesting intermittent monitoring only
- I'd prefer wireless if I needed to be monitored again
- Wireless to enable easier movement throughout contractions.
- Research what would be less invasive for baby
- No way. Next time I will have very minimal monitoring done as I believe it contributed to my emergency caesarean. Very stressful
- I didn't make an informed choice. I just went along with what I was told.
- No epidural or induction
- I didn't find it necessary or beneficial. If I had to do that birth again i would only accept to hear baby's heart beat once every 15 mins or so
- I would prefer something wireless if available. But don't think that was an option at the hospital I was at.

- My baby was fine. I feel I could have Been a lot better if allowed to stay in other positions
- I would steer clear of CTG monitoring and trust my baby is happy and doing what it needs to do to get through the birth canal on their terms. Intermittent Doppler monitoring I would have again during my labour.
- I do not want to be restricted by CFM or have false alarms causing unnecessary interventions.
- I had a really easy labour and birth experience, next time if I had a similar experience I would like the same level of monitoring, but if I had a different experience, I would want monitoring to be commensurate with risk level
- I agreed to CTG due to trust in my private midwife - and it gave me more freedom within my setting. Otherwise I would stick to doppler only next time
- I would want wireless monitoring so I could move to ease the pain.
- Periodical checks so I could labour in all positions rather than continuous
- I would prefer only intermittent monitoring so I could move more freely & attempt a water birth
- I didn't feel in control all of the time, I felt I had to do as the OB wanted even if my comfort wasn't 100%
- I'd ask to not be able to hear it if possible
- Through much negotiation I was able to decline CTG monitoring and have a hand held Doppler used every 60 minutes through two contractions.
- I did not want a scalp clip and would not consent again
- Movement
- I would choose not to be touched to monitor contraction strength but as I was induced I do believe continuous monitoring was safest for baby
- No labour so no impact on myself a d I was happy that my baby was being so closely monitored
- I think I will insist on intermittent as it was not invasive, and I feel more confident now pushing for my evidence-based choice, rather than just following hospital policy.
- I would choose whichever is best for my baby
- Wireless that works, stick on looks good rather than straps which are awful and move around too much
- I would probably be more forceful in stopping the every 15 minutes
- Would opt for intermittent monitoring so I could move
- If I had to be monitored, I would try a wireless option, or where the midwife checks as that would allow more movement during labour and also would allow the midwife to have a more hands on physical examination so that crucial things are not missed
- I was happy with the wireless monitoring every four hours. It allowed me to labour the way I wanted to (in the bath, and walking around) - it was only after 24+ hours when things went a little pear shaped and I was eventually transferred to a birth suite for continuous monitoring as the baby was in distress.
- I don't think there would be other options at this particular hospital as it is fairly small. I would prefer an option that was wireless or stick on.
- I wasn’t consulted at all, just told it’s procedure
- It was painful and failed to give a reading making everybody angry and yell,

- Only doppler
- I would hope the technology for wireless monitoring improves and I would be more vocal about asking why I needed to be monitored all night before the induction
- I would choose wireless so I could keep moving
- I am not familiar with the other forms of monitoring and know that the wired form is effective. I used it for both my babies and they were both delivered healthy and safe.
- I would decline continuous monitoring and push for intermittent auscultation/doppler
- I would like to be able to move around and use water for pain relief
- Would be happy with wireless monitoring, would request not for entire labour
- Definitely would choose wireless monitoring if available.
- I would want a wireless option at the very least, something secure so I was able to move about
- I had an epidural so had to stay in bed. If I had been mobile anyway I'd have wanted to move around unencumbered by wires
- I want to be able to move around during labour and not feel restricted to a bed
- I would do whatever is safest for the baby's wellbeing
- I wouldn't choose it at all if there's an option! It was presented to me as non negotiable
- I hope I won't have to be induced again, but if I do I preferred the internal monitoring because she was constantly readjusting the straps and monitor before hand
- I wasn't given a choice. It was "we need to monitor the baby's heartrate and this is how we are going to do it."
- Peace of mind due to complications during first labour
- I felt restricted and unable to move around freely, part of my original birth plan was to be able to move around and use the exercise ball during labour
- Continuous monitoring doesn't improve birth outcomes. I would rather have freedom of movement and have intermittent monitoring with a hand held Doppler for my next birth
- I wanted to do positions that I felt comfortable with but the shape of my tummy meant the straps kept shifting and I spent more time worrying about that then actually relaxing into the moment with my baby to progress as a team in birth
- Monitoring is reassuring but would like another option
- I would ask for wireless monitoring
- Wireless, easy to use
- Want ability to change position, use of water for pain management.
- Wireless
- I am due in 4 weeks and will not consent to continuous monitoring because I believe it contributed to my emergency c-section by being bed bound, uncomfortable and stressed during labour. It led me to an epidural to manage pain which I did not want. I hated it.
- Wireless if available so I could move around more
- I would enquire about wireless monitoring
- It inhibited mobility. Distracted me. Caused distress unnecessarily.
- If I had another baby it would likely be a freebirth, so there would be no monitoring

- Preferred wireless but was unable to use it due to the machine not being charged
- I have chosen to freebirth my next child (due in a few months) with a doula, my partner, and my sister. I do not trust the health system to protect myself and my baby and believe birth is a natural process that occasionally needs intervention. Intervention (including fetal monitoring) should not be normalised. Most women are safer at home away from the red tape of hospitals (unfortunately hospital birth, or free birth are the only viable options in my area).
- I would strongly ask for intermittent again. Only going to continuous if there seemed to be a need.
- Traumatic birth with many complications so hard to say
- Because I didn't have to worry about pads slipping or wires getting in the way. I could walk freely if I wanted and could use the Toilet
- It meant I got my baby girl out before things went south
- I didn't want interventions but got stuck with a cascade of interventions the minute I entered birth suite. Told mid push that I had to have forceps or caesarean and had to choose now purely because babe heart rate had slightly dropped with contractions likely because given pitocin drip even though I said I didn't want it then CTG even though I didn't want it
- Same monitoring with no induction medications
- If I had an epidural I would happily have the same type of monitoring again. If not, then I may prefer more freedom to move around, get in the shower or bath, etc.
- If I could move around with an epidural (not allowed at the hospital I birthed at even though I could feel my feet/move them) then wireless would have been beneficial.
- I was having an induction so it's very important to have CTG monitoring
- I would ask if wireless monitoring was available
- I would ask for wireless monitoring
- For my second labour I opted immediately for a scalp prong to be placed so I could have wireless monitoring. This was far more comfortable for me and I had no issues.
- I understand there was some medical reason for monitoring so I can't really say I wouldn't have the same monitoring but it was still extremely distressing for me and absolutely contributed to my labour stalling further
- Impacted my ability to move
- I'd go with what was recommended for the situation and reason I needed to have it
- I didn't want it! I refused it! I yelled to not have it. I asked to not have it before it was put on.
- At home irregular monitoring was okay with my midwife who I knew well. At hospital wireless monitoring equipment needs to be up to date and midwives need to better trained in its use so this is a real and workable option.
- I would like to be able to get pain relief from shower/bath
- No monitoring
- I wouldn't have any constant monitoring, just some monitoring from a midwife when needed with a hand held device
- Ultimately the outcome for us was positive with a healthy baby - I am unsure in hindsight whether the emergency c section was truly necessary and the continuous monitoring influenced the chain of events that led us there quite heavily. Without a crystal ball, I don't know whether an alternative could have resulted in a different

outcome but I am still happy with my birth experience although it was stressful at the time.

- I want to be able to move freely during labour, I also worry that Doctors will be too quick to do a c-section and use the ctg results as an excuse, I also found it intrusive and inappropriate that my ctg trace was being monitored in a different room by doctors I'd never met.

4. Now that you've had your baby, what do you wish you had been told about monitoring during labour?

- The baby can hear the high frequency even from a doppler and we don’t know the effect of that
- Definitely more discussion around reason for monitoring and options.
- Just a better description would have been helpful. I didn't really feel like I had much of a choice when it came down to it.
- It felt like there was no choice given to me- and it prohibited me from having a water birth
- More options ie wireless, when it is and isn’t necessary
- I wish I was given the option especially as the wireless ones were out of use
- What happens if there was different monitoring eg doppler or intermittent monitoring
- That I had options
- That its ok to not have to be strapped to something and there are other methods!
- Any education/information would have been good
- Yes. But not by hospital staff.
- that i could have a wireless option.
- How frequently the sensors move and need adjusting
- Why it was used and what the medical team were trying to ascertain (and whether they suspected a problem)
- No
- That there is no evidence that continuous monitoring improves outcomes, and significantly increases caesarean rates. That it would be uncomfortable, restrict my movement and pain relief options. That intermittent doppler is a safe option.
- That it was going to happen and that wireless monitoring was an option
- That it meant ppl always in the room
- That there were options
- That CTG traces can be difficult to interpret and that the more the sensors move around the less accurate the traces are. My baby was deprived of oxygen for a significant period of time during labour DESPITE continuous CTG monitoring indicating nothing out of the ordinary. At no point in time (despite the monitoring) was I given any indication that the baby was in distress. After a brief issue with shoulder dystocia (less than 1 min; not long enough to result in observed pH and lactate levels) baby was born with an APGAR of 0 and required 4 minutes of CPR. He was transferred to another hospital for hypothermia treatment in NICU while I waited 12 hours for a bed to become available and transport. I thought AVOIDING these things was the reason WHY hospital use continuous CTG monitoring!
- That there was different options available

- N/A
- Nothing
- Evidence based reasoning into why it was required - as well as how that related to my own personal situation.
- Nothing different as it didn't impact my labour but I may feel differently if labour did go as hoped
- That you can decline monitoring or to not have heart rate checks so frequently and not have negative reaction by health care staff
- That monitoring doesn't have to mean I'm stuck in bed on my back
- Nothing as my midwife followed me throughout my pregnancy and kept me informed every step of the wat
- No
- I wish o knew I could get in the bath and shower with the monitors
- That I could have refused if I choose
- I wish I had been offered wireless monitoring. I had wanted to use water as pain relief but was unable to due to the monitoring they wanted me to have. My labour progressed very quickly so that was one upside, that I didn’t have to be monitored for long.
- To see the statistics, and understand why. It’s not to save my baby, its to save the hospitals backside
- That I wouldn’t be able to move around freely. That the monitoring determines what intervention can and should be used according to policy. That it can fall off and provide an inaccurate reading and the medical professionals still base decisions on it when it drops out.
- Sorry, I've found this survey very hard to answer, as I didn't labour for this pregnancy, but I was monitored prior to a planned caesarean. I had monitoring used frequently in my last few weeks of pregnancy - I didn't feel it was any different on the day I gave birth.
- Nothing. I just rolled with what I was being advised to do
- I wish my midwife had respected me when I said I didn’t want CTG monitoring. It started the cascade of interventions that I didn’t need or want and left me with severe PTSD.
- I knew my options but standing up for what I wanted was difficult
- That it really isn’t necessary, it’s not for the benefit of the woman or baby, only the hospital
- Nothing. Had the same monitoring throughout pregnancy for my first and second child. Monitoring allowed me to try naturally 3 weeks early with my first but resulted in emerg c-section & went for emerg c-section 2 weeks early with my 2nd child due to similar circumstances.
- Not all labouring positions are possible, even with wireless monitoring. That it can be annoying even when it’s working.
- The cascade effect of not being able to be active in labour
- Just all options available and when one may be required so i could make a more informed decision instead of feeling pressured to have electrode monitoring.
- Warning about how uncomfortable it would be

- Nothing, I educated myself. Had I relied on scare tactics I doubt I would have achieved the natural VBAC that I did.
- That hospitals require a good CTG to be able to use the bath and if they can't get one they won't let you in
- It is optional
- Nothing i was happy and made the choice to have all monitoring internally and my obstetrician was supportive of this
- What would happen in the instance that manual listening with a handheld Doppler presented a concern what would have happened next. Is monitoring routine hospital policy?
- That there were other options
- What it involved, why they wanted to do it, how it was helpful for them, what the benefits and risks are
- There are different monitoring options, the amount of times they like to monitor as standard practice and what the least amount of monitoring they will do for a normal birth, why they need to monitor and what the risks are
- It's not necessary non stop
- I felt informed and glad that I made the decision of intermittent over continuous monitoring.
- I was well informed
- Where the wireless version was & why there aren't enough to go round
- The statistics of how monitoring affects birth outcomes
- That it would limit my movement and what other options I had.
- That when discussing being induced that I was told I would be constantly monitored
- That you have a choice and constant monitoring isn't needed during vbac
- Not so much told, but I with wireless CTG monitoring was available within my hospital
- This is a difficult one as I'm not sure whether it contributed to my emergency c section or not. There may even be divided opinions by professionals, I'm not sure. After more than 40 hours of intense contractions at somewhat irregular intervals, dilation to between 5-7, syntocin was being mentioned but not delivered. Midwife ruptured membranes, and was told not to push even though urge was starting. Given epidural to allow syntocin, but the latter never eventuates. Obstetrician came and recommended c section as heart accels and decels were too shallow. Bub was born with APGAR of 9... so did the monitoring lead to an unnecessary c section? I don't know!
- Not that I can think of
- If I wasn't a midwife I would want to be told what options are available. An explanation as to why I would need continuous monitoring, being truthfully told that wireless is actually rarely available
- What the lines mean
- That you had a choice key reasons for it and expectations on how it will work upfront
- Yes
- I already knew that continuous monitoring doesn't necessarily improve birth outcomes but I felt that I needed to be monitored as my baby was maybe more at risk due her Down syndrome diagnosis and small size (reason for induction)

- Whether it was truly important to be monitored at that moment in time
- That it wasn’t always necessary. At one point I pulled all the cables and straps off which apparently alerts the midwives but no one came to reattach them. After that I figured they really weren’t needed.
- How uncomfortable it would be to get set up. And that once I was in the labour suit they would want to use a CCG monitor continuously
- I wish that my request to decline had been respected.
- Everything
- That it doesn’t necessarily need to be continuo
- That there was wireless available
- That it wasn’t compulsory or that there were other methods available.
- That the CTG was continuous and not always accurate and that they may want to have other forms of monitoring
- No
- I wish I knew that they no longer had the wireless monitoring. I would have refused continuous monitoring in favour of intermittent monitoring or turned up just in time to push the baby out!
- Being my 4th baby I felt pretty well informed. I do however wish more women were told it an aspect of care that they right to not consent to if they don’t feel it is right for them and their baby
- Nothing.
- That the CTG often doesn’t work well on posterior babies!!
- That I could have intermittent Doppler monitoring. That I could decline the use of continuous monitoring. That it was a choice and not a necessity.
- That although recommended its ultimately my choice
- Nothing I was well informed
- wish I had done a better preparing course, and prepared my body better, preventing the prolonged second stage, therefore not needing the extra monitoring.
- That i could say no
- The effects in your ability to labour, the fact that it’s not evidenced based, can be wrong and that the people using it have now lost the skills to assess birth without it and that the research shows it does not actually prevent death or CP
- Not told more, wish I Stood my ground more
- Nothing. I was well informed
- N-A
- More that I could have asked the midwife to stop checking every 5 seconds on me- because the new mid shift barely touched me because she could see I was labouring- not needing to feel them constantly
- The benefits for the baby
- I was happy with the knowledge that I could decline the monitoring and my husband was confident in doing the same.
- Nothing, being a doctor who works in O&G I already knew all the guidelines, however I'm hoping that if a layperson was in my situation they'd be discussed why the monitoring would be done
- Nothing- I was very well informed

- I wish I had been listened too when I asked to not have my baby’s trace on the central monitor
- I felt really well informed by classes, my midwives and my obstetrician
- Information about my specific risk and why it is indicated without having to ask however I was only given brief information when asked and was lied to antenatally and told I would be supported to have intermittent monitoring which the care provider refused to do on the day
- There is nothing that I wish I was told that I didn’t already know.
- That i could opt out
- That it doesn't always give accurate information. Can lead to unnecessary intervention
- I am a registered midwife. I was well informed. I wish I was more able to advocate for myself
- N/a
- My alternative options. That I had a choice to say no
- Nothing extra, I felt well informed.
- Perhaps that it’s optional and that you can choose which way you would like to be monitored
- How it can affect your labour
- Everything!
- That it is not comfortable and can be quite intrusive with all the extra hands touching you trying to keep it working properly
- That it will pull focus from where my mind should be during labour. That it makes people come into your birthing space far too often to fix it up. That it limits movement even if wireless because it moves around so much. That it allows the doctors too much influence in your birth which can lead to interventions and cesareans.
- That I wouldn’t be able to take it off. That it would interfere with me getting the peaceful natural birth I wanted
- That I wouldn’t be allowed or able to use the birthing pool
- The different options
- I was well informed
- That it wasn't compulsory
- I felt I had enough information
- I wish women knew the advantages and disadvantages of different forms of monitoring. Women often don’t know they have a choice, or aren't told why they have continuous monitoring or how that could impact the rest of their labour
- That i could have stayed home and had my baby. If i ever have a long labour again i will be more active, well nourished and hydrated throughout the labour and implement different ways to aid my labour
- How uncomfortable and restrictive it is, how unreliable the readings are and how care providers can misinterpret the information.
- What options existed for which scenarios and the implications of each
- The risks and benefits, all my options of monitoring
- That I could decline it and that it wasn’t necessarily needed.

- I wish I had been able to have a conversation about the risks and benefits. I was just told I had to because I was being induced, and I was so scared that I agreed. That's not the right reason to make a medical decision. It has been another factor in the ongoing mental health concerns I had after the birth of my first child.
- How to understand what's high and what's a low reading
- Nothing, I was told the appropriate info
- I should have been told that it would be a distraction. I believe that the midwife should have been able to see my labour slowing down and made suggestions to help it along. I think that women should be told that the monitoring could impact their movements during labour, even if the monitors are wireless. Lo
- Nil else
- That the doctors and midwives Clotilde acknowledge that the benefits of monitoring in my case were actually unproven and could potentially do harm rather than good. But more that I hadn't caves to pursue and sick to what I know about monitoring in labour.
- That it was compulsory. That there were other options like wireless or none at all
- If wireless monitoring was an option
- I wish the midwives had told me I would have to be monitored the whole way through my labour and that it would mean I couldn't have a shower or get in the bath instead of the uncertainty of thinking I would only have to have it on for a short time more and then they would suggest continuing with it and not answering me about whether I could get in the shower and avoiding giving me an answer. I found it infuriating.
- Actually how regularly to expect monitoring but also to not be alarmed but so frequent monitoring when an spike or deceleration occurs because labour needs to happen for birth to happen
- I had all the info I needed, however I did feel like every time the midwife pressed dipole into my abdomen another contraction started.
- That it was an option and there were different ways to do it rather than straps
- I wish I'd had more explanations of what would trigger an intervention and what that intervention might look like. I wish this information was communicated to my family members as well, so they could remain calm.
- Not sure, being a midwife I knew it was a requirement for meconium but I still felt like it didn't help nor hinder my labour at all.
- I was happy with my decisions about monitoring
- I felt fully informed
- I would have liked more general knowledge about the different types of monitoring, indication, risks
- I just wish I pushed more for the wireless monitoring, more movement could have led to less intervention and potentially then not an ECS
- It was my 4th baby and all low risk so I knew what to expect
- Nothing additional
- I was well informed by my midwife before labour about what monitoring what be done and how. It was a bit uncomfortable if a contraction came while she was monitoring, but that was a very minor inconvenience.

- The monitoring was not traumatic for me and I had a midwife who was able to locate wireless CTG. The coercion to have augmentation and then the hyper stimulation of my uterus was the traumatic part and the thing I would change.
- Needs to be clear that it’s a choice, not forced upon women
- Nothing, i just wish the hospital at the resources for either wireless / waterproof monitoring
- The purpose, options for types of monitoring
- I am a midwife and felt I had all the knowledge available
- How much readjusting is needed and how it can restrict movement and access to water (shower).
- More info on why they want to listen so often.
- I felt informed during this labour and birth and my consent was requested prior to any monitoring.
- Nothing
- I was very happy with what i was told and how informed i was.
- Intermittent doppler was fine and I didn't even notice it
- How easily they can move around and come off, it really restricted my movement and had a negative effect on my labour
- Nothing, I knew a lot about it already
- I was well informed as this was after a very traumatic first birth
- Yes
- I feel I was given adequate information
- How disruptive it can be and also how traces have to be interpreted and there is subjectivity in this
- Nothing. I was fully informed
- Nothing extra
- I knew- but there is no consent and is no choice- it’s just ‘the way it’s done’ which I will never agree with
- The midwives made out it would be uncomfortable and invasive with continuous monitoring. I found it completely the opposite.
- More specific information about my baby’s vitals and mine as well to understand what’s happening. I like to know
- NA
- It’s can be quite uncomfortable and distracting so only use it if really necessary rather than as part of the hospital routine process
- That it was not always reliable and that there were other options. Also that I could decline continuous wired monitoring and ask for an alternative
- Because I have a history of pregnancy loss, it would have been nice to not be so focused on 'emergency baby monitoring' and just a reassurance thing. I wish I was given more opportunity to decline.
- Not for me - but all women ought to be told that there is very few scenarios where continuous fetal monitoring is necessary or has proven efficacy. Birth interventions should not occur for the convenience of medical staff or as a result of "litigation mitigation" strategies.
- Risks of continuous monitoring. Knew some, found out more later. Not relevant to my births but still. Good stuff to know.

- I wish the midwife looked at me instead of the monitor. I wish I was told I could take it off. I wish I was told that that if the monitor is on, they look at it to see if I'm having a contraction instead of looking at me. I which I was told that monitoring showing contractions further apart than they expect for active labour would mean the midwife leaves the room, without any other check.
- It is only reliable in certain positions, which will make labour harder because midwives focus on the monitoring being accurate over the mother's comfort
- That the fetal scalp probe was screwed in to his head. They didn’t explain it like that. The risks of monitoring and the increase likelihood of emergency delivery
- That I can say no!
- That I have a choice!
- That it is not evidence based practice and can lead to higher intervention rates.
- That wireless was an option and when it was that i could actually get up and got to the toilet if needed
- I wish that I’d been told that I would have as much reassurance as a cow going to a slaughterhouse this was the worst experience of my life and I’m still suffering
- This more applies to my first birth (8 years ago) in hospital, but you do have the right to decline continuous monitoring. I found it extremely uncomfortable.
- Nothing
- I did get informed of monitoring in labour
- Nothing
- Nothing really
- Pros and cons and offer the choice, but mainly being listen to when I needed to be free to move
- Nothing if there was wireless I assume they would have mentioned
- I was very well informed antenatally so I felt like I had all the information going into my labour
- Explained the different types of monitoring available
- More options
- It can cause more harm than good - a watched pot never boils.
- I was well educated
- I wish I knew what the internal monitor looked like and ACTUALLY involved for my son. Its not placed ON its placed INTO his scalp - there is a difference and saying otherwise is lying and not informed consent. I wish I had declined constant monitoring and only allowed my midwife to check intermittently. We did tell the original OB not to enter again unless there was an emergency as I believe he would have used the monitoring against me and pushed for induction again. He told me hed get the drip started when I first arrived and I declined when I realised he meant to induce me for no medical reason. The CTG at the time felt like the lesser issue and i accepted.
- That I had a choice on what type of monitoring I had. They didn’t give me an option to not have it
- I had knowledge about monitoring, but it was done without consent & this was the main problem & only due to policy (length of second stage)
- I felt informed
- Yes

- Nothing. This survey seems to be hinting at monitoring being a negative thing and I'm unsure why. Monitoring kept my baby safe especially during some scary parts of labour - including at the end when my baby was going into distress and so she was delivered quickly via forceps. A little discomfort with some wires and straps to ensure a safe and healthy baby is fine by me.
- More information about how results will inform the rest of the labour
- That there is choice
- Intermittent checks would have been better than continued. Wireless would have been so much better
- The risks
- I feel I had enough info
- How long it would take. When I went in for check ups I'd get monitored â€˜for a little while' which could be 30mins or 2hrs.
- I would have liked it to be explained in more detail and perhaps the option of turning it off for a while to manage my anxiety
- Nothing more than I was
- That it might be used to justify intervention which was perhaps unnecessary.
- If I could have declined continuous monitoring and what other options there would have been
- I was given good information, list all the benefits and give the parents a show on how to read the results
- Could I have used the shower or laboured in water?
- That the mid wife would be in the room the whole time, would have been nice to have been left with my husband to labor together
- The scalp 'clip' is actually screwed into your baby's head! I would not have consented to that if I knew it penetrated her skin. I also wish I knew more about the pros and cons of monitoring and the validity of choosing not to do certain types of monitoring or any at all.
- I was happy with the information
- I was told as it was planned
- anything, I knew nothing to be honest
- The real stats and medical facts of my situation to help correctly weigh the risks and benefits of such an intrusive form of monitoring.
- I feel like it was framed as necessary which it might not have been. In saying that after all the carry on fighting to not have CTG I felt comfortable with its use.
- I knew it all already but once I transferred I was at the mercy of the hospital and its policies. Also the hospital never really explained the monitoring I just didn't ask because I was already well informed but they didn't know that fact
- Na
- I had all the info i needed. The flu was a complication that I needed more info for but I was already in advanced labour anyway...
- Nil
- That I could've have chosen to have different types of monitoring like the midwife regularly checking the babies heartrate herself.
- I was happy with the option to have intermittent monitoring with the Doppler

- That people would be watching me outside the room and making judgements and decisions on my labour
- I'm certain it was the right decision for my and my labour.
- Nothing
- I was well informed. I never would have consented to a CTG if I were in a hospital environment.
- I wished inductions of labour were fully discussed with all women as being a medical intervention requiring a lot of monitoring and checks and not just as something that is normal. I did my research and declined induction of labour even when I was scared to death by the doctor and opted to have a beautiful water birth instead.
- Why and when?
- At the time believed I must have needed it but looking back it was just hospital policy
- That even wirelessly still makes it awkward to move around
- Yes
- Not really, I accept that this was necessary for the health of my babies
- EVERYTHING
- That I wouldn't be able.to.move about or even get into the positions that I wanted to be in on the bed and was constantly told to get onto my back even though O knew it wasn't what was best for me
- Not sure
- If it could be done during a water birth or while in the shower
- Given more detailed information
- How wireless monitoring isn't that great and I would still have midwives fiddling getting to work
- I feel I had been told everything I needed to know. I knew why they were doing it, and it was a reassurance to me to see her heart rate going strong. It was a little scary when things started to spiral downhill, but it was great that the midwives could see this and they sprung into action.
- About the on the head one
- That I had the right to refuse or request a different type.
- Nothing
- Indifferent
- That there may be a wireless option!
- Why it was needed the whole time.
- That there were other options
- I would have liked to have known rationale for doing it and that I'd be monitored if induced earlier
- Options!
- I was fully informed and don't think more information about monitoring was necessary. Maybe just more info on what was available at the hospital during antenatal classes. But I don't think that would have changed anything for me.
- how the fetal scalp monitoring was used and attached to baby's head, before I was in labour so I had time and the mindset to process information.
- I wish they would have listened to me.
- I was fully informed

- That it would occur and why. The different options that were available and if they was a choice
- I wish any risks had been explained to me
- Nothing
- If induced told constant monitoring was necessary, wound have declined induction
- That the scab on my baby’s head would take quite a while to heal
- how it works, what staff are checking
- More about signal changing
- That i would have to wear the monitoring equipment for 24hrs non-stop.
- The impact it would have on my ability to move around freely and how they would make labour more uncomfortable
- Anything
- That’s there are multiple options
- To use wireless so I could walk more and let nature help
- I think I had the information but didn’t fully consider the impact on myself & stress levels
- Why, what the results mean
- Yes
- Wireless option. Given a choice to monitor or not.
- Not sure
- Nothing - I had excellent care in my very speedy delivery
- Different options for monitoring
- When it was warranted so that I could advocate for myself as I wasn’t offered the option or an alternative - I would have been happy with regular Doppler monitoring
- Why i needed it, if it was necessary, if i had a choice
- That it is standard practice and what they're listening for
- How often it is done (eg Doppler with each contraction)
- More discussion about reasons for monitoring
- It can be annoying!
- Unsure
- Anything at all. They didn’t tell me anything about it except that it would listen to my baby's heart
- NA
- Had epidural for first baby (not the one I'm discussing here), didn’t know that would mean stuck in the bed with continuous monitor
- The options and reasons why
- Na
- That there were more options than being attached to a wire
- The baby's heart rate could be dropping
- What impacts it would have! We were told about your birth ball, the bath, movement, breathing but not - hey all of that stuff is useless because you'll be strapped to the bed most of the time
- Why they needed too and they could have asked and said why since my other 3 I didn't need monitoring at all. (water birth was explained and perfectly fine)
- Nothing - I felt fully informed
- That there is a wireless option particularly for the scalp clip monitoring

- Yes
- It's optional.
- That wireless machines need to be charged beforehand.
- That it can be super uncomfortable when on for a long period of time and may cause me to stress out more as I was unable to move due to the monitor not picking up the babies heartbeat unless in a certain position.
- I would have liked more info about what monitor data meant and how to interpret. It was terrifying when baby's heart rate dropped out for a few minutes, following this it would have been reassuring to know what the numbers meant.
- Yes
- There are more options than a trace with a belt and wires or a clip with wires.
- I had all the information I needed
- You have the right to make an informed choice
- More about how it works and how reliable each type of monitoring is
- Nothing it's just needed
- What the numbers and we're and what they meant so I could understand it
- I wish I was informed of options and if it was completely necessary
- I'd like to know if fluctuations in the baby's heart rate are normal during labour
- That it would be needed the entire labour. That there were options in terms of monitoring available. To even be asked if it was ok to use wired monitors would have been good.
- That it's not really necessary
- That different types of monitoring can be available without endangering my baby. That there is a choice
- Nothing- I was fine with it
- Whether it was necessary and why (in my case it gave early notice of complications, but also caused severe anxiety as labour progressed)
- That it would draw blood and leave a mark in his head
- NA
- That I could choose to take the straps off! It felt very restrictive!
- Midwives have no right to limit or dictate your ability to consent nor weaponise your treatment for their easement. That you can opt not to monitor.
- Why, what they are looking for, benefits
- Whether it was a choice for me, how important was it during my labor.
- Internal fetal monitoring
- Wireless as an option
- I'm glad I was monitored the whole time. I felt it was best for me and baby. I feel all babies should be monitored throughout
- It isn't necessarily needed continuously
- I feel my private midwife had me well informed but the public hospital did not
- That it was optional
- That the sound could be turned down
- That it restricted movement
- Had to stay out of the bath
- That you have a choice. I thought it was compulsory - for safety

- That there was a chance that they would have to attach an antenna? To the babies head to check for heartbeat and it may leave a scar.
- The risks to baby with the scalp clip. That I would need to have continuous monitoring and what options were available to me.
- Women need to be given more choice and more education. The need to be more empowered to speak up and demand better conditions for birth. This would result in less intervention and better birth outcomes
- The choice and ability to refuse the suggested types and ask of alternatives
- If there were other options
- I wish they had done the FSE earlier, and I had been given more information about it, rather then waiting towards the end of my labour for my CTG monitor to continually lose contact and for my labour to be disrupted so much.
- That monitoring is related to risk and that i and baby were actually at risk during my long labour. I ended up with significant ongoing pain due to the labour and baby had Jaundice
- I wish I had of been told how necessary it was, did I really need to be monitored?
- That I had a choice rather than told if I didn't I would need to consider a caesarean
- Nothing - I'm happy for monitoring your occur. A healthy, live baby is top priority.
- If it was completely necessary
- That wireless is an option
- How necessary it really is. I feel the midwives go to these monitoring devices and then â€˜set and forget'.
- Length of time monitoring would occur for and what actions are taken based on monitoring results
- nothing really, it was for my baby so I had no objections. Next time I would love a wireless machine
- Not really
- I am ok with what was provided
- That evidence shows it does not improve outcome for baby or mother.
- With an epidural you're not able to walk around and need to lay on your back, so monitoring with wires does not impact the situation.
- Nothing
- Pros and cons of various options
- YES
- That I have the right to ask for breaks, or that if baby is tracing well that I can request Doppler instead.
- That i had a choice!!! To say no
- It should be discussed in the antenatal period. I had no idea that having a low PAPP A would require CTG monitoring. I'm glad I had experiences two previous labours/births and felt comfortable to say no
- Nothing
- Nothing else
- The circumstances it might be require, i didn't know before labour that i would need constant monitoring.

- There was a power outage at the beginning of my labour and the midwives weren't able to use the wireless monitors, only the wired monitors connected to the generator power.
- I was happy as I had an independent midwife who gave me hours of time and information. I feel sad for those women who have rushed 15 minute appointments who can't talk these topics through
- That I would need the coil
- Optional
- That there were different types of monitoring, my labours are fast so i was always told we can set up the wireless ones later, but by that time its too late for them to switch it over
- I wish I knew there might have been other options (although my hospital may not have had them available at the time). I wish I had known about any risks.
- Did I have a choice? I didn't feel like I had any choice. I wish the wireless monitoring I was promised had been provided.
- I was fully informed
- Wireless choice
- No more than I already had
- Yes
- That it rarely works properly.
- Nothing. I was well informed by my midwife.
- Informed and given opportunity to consent or decline. Education about other options.
- That a water birth wasn't possible
- That I'd been informed I had a choice.
- Yes
- That there can be different ways of monitoring that you can request instead
- Yes. I was not told in my antenatal visits that my "high risk" status would mean I would need to be monitored so much.
- It might have been nice to be really clear that my request for limited intervention would include periodic use of the Doppler, but it didn't really impact the overall experience.
- The machines numbers would always overrule my preferences. So If I had my time again, I wouldn't have let the hospital know my waters broke early so I wouldn't be induced. Or I would've fought harder for a c-section.
- That I can decline
- The wireless choices so i was not limited to the bed
- How long I would be monitored for, when I would get an update on results (difficult when the OB is required to deliver results/ outcomes). What an adverse result would mean/ next steps to mentally prepare. Though this is a fine line between scaring mums and preparing them.
- The best birth is an active birth, and your comfort levels and positive hormones help to progress labour.
- That continuous wired monitoring was not needed, as babies were not distressed, that monitoring would limit my movement and that this would impact on my labour

progressing, that I had a choice in monitoring strategies, and that I should of been informed before these were undertaken.

- Be more understanding of what it was reading.
- It's purpose, how I could cope better with the discomfort
- Nothing
- I wish I had been told that I didn't need it on all the time, and that it was my choice to take it off and put it on as I wished. I wished I had been better informed about the monitoring methods available at the hospital. I wish I had read up a bit on what the "data" from the monitoring could mean, for example how "serious" is it if a baby's heartbeat decreases during a contraction.
- That it occurs as soon as your waters break if there is meconium in them.
- Would have been to good have info to make an informed choice.
- Why they were doing it
- Nothing. I was happy with the monitoring
- That wireless was an option. That wires could be attached directly to baby with enough time to actually talk about it.
- That it was optional, what the risks are of not being monitored, why exactly being induced meant I needed monitoring.
- that there is wireless monitoring
- I wish I'd known how they attached the sensor to my baby s head beforehand
- It's painful. It is limiting. But breathe and trust your instinct, don't let the midwives just go off the machines, get checked if unsure and really request to be checked, don't let them brush you off
- If there were other options
- From my first baby who had continuous monitoring j was pretty aware of the information around monitoring.
- I had enough information
- Nothing, i wouldn't change anything about monitoring during my experience. If no epidural and free to move i would love a wireless monitoring though
- There was no option to not do it. So this doesn't really apply. My baby was in potential danger so we had to monitor
- How to ignore to focus on labour, reasons care providers might want it
- That it might lead to other unnecessary interventions.
- That I had options.
- More about the different options available and the pros and cons of each
- How restrictive and benign it is.
- Risks of CTG
- That it's optional.
- I wish that I had a low risk pregnancy and to be told I didn't need constant feral monitoring
- why it is necessary and what the other options are
- I wish the doctors had explained more what they were concerned about and what parameters they were not concerned about.
- The process + pros/cons of internal monitoring
- About the different sorts available.
- Nothing

- I was not given any information about the monitor attached to baby A head and any risks to him
- I think I was entirely well informed, by my midwives and previous labour, and my own research,
- I didn't really have any info, I didn't know different options
- I felt I was adequately informed
- Just the impact of having it and how to prepare coping strategies that work with it.
- Nothing it didn't bother me
- Previously listed that its not necessary in a normally progressing labour low risk with no complications
- I wish I knew how to read and understand the machines and numbers
- I feel I had been given all the information, but found the obstetrician to be quite pushy
- I didn't realise it would be on the whole time even when pushing. Just makes it uncomfortable but I know why they have to do it
- that it would limit movement, limit access to water and that it hasn't actually done much for maternal and fetal outcomes except increase the Caesarean section rate! INFORMED CHOICE is EVERYTHING
- That it was a choice
- That I can accept or refuse it
- That when labour is in second stage and baby is already so low and about to crown that it is difficult to auscultate the heart rate and tracing can be unreliable. Why advise a scalp clip when birth is going well and there are no reasons to be concerned.
- I wish I'd had more options
- Nothing additional for this pregnancy. Being a second pregnancy I felt I had a better understanding of what it was and would have happily chosen different monitoring options if a wireless option hadn't been available
- I didn't /don't know what's wrong with monitoring apart from wires/wireless
- I can say no
- That is was optional
- That it wasn't necessary all the time. That I could decline it. What it was actually showing
- If there were other options (wireless would have been great). Being offered a break off it so I could have showered or got in a different position.
- Being bedbound and agreeing to intervention would impact my ability to vaginally birth
- That it screwed into my babies head.
- That I had a choice of monitoring systems. That I could decline to be hooked up to a machine constantly
- Nothing I knew everything I needed to know
- That there is a choice, and that a conversation should occur.
- Nothing but of course being a midwife birthing I was in a position that I already understood all my options
- That if induced I would have continuous monitoring and not allowed in the bath/shower
- Nothing, I just wish I had the courage to decline intervention

- Nothing
- any information would have been useful
- Nothing. I like it. It reassures me that baby is okay during the whole process
- That a wireless monitor might not be available. Walking around with a machine was hard.
- That while the electrode was more accurate it may effect the baby or cause a trauma to their head. My baby was vacuumed and had marks on her head afterwards already so I felt bad for her that she might be sore.
- That there are different options
- The risks of infection from the clip and that there were other monitoring options. I was only given the one choice during my active labour. There definitely needs to be more information provided to expecting mothers. My poor baby had a very bad infection and has a scare on her head from it. Such a traumatic experience overall. I wish I knew then what I know now. Things will be very different for my next birth.
- It's not evidence based
- If there were other options and what the negatives of being monitored might have been
- Yes
- That there are adhesive CTG wireless monitoring available
- Yes. I believe knowledge is power
- That it didn't seem to bring me comfort so that i had a choice of not doing it. Also that the clip they use when baby is descending and CTG can't find the heartbeat is actually an incrusted metal
- Just if there was a wireless option.
- You have options, that it's normal for baby's heart rate to decelerate during a contraction and as long as it's recovered afterwards then baby is happy and healthy. What is normal range for baby's heart rate during labour.
- I felt well informed as this was my third baby and I have also done a lot of my own reading and research regarding all aspects of labour and birth.
- That it increases the rate of all sort of interventions and that intermittent monitoring is an option (this was never offered to me)
- How necessary it really was. Risks to baby by not being monitored. How restrictive it was going to be.
- That its optional or there are options for different ways for it to happen
- I was happy with the level of info provided
- That the studies show that CTGs aren't actually safer than doppler use and can increase use of interventions eg instrumental/caesarean
- In some ways the internal one was easier to move around with. That we didn't really need it done.
- That I could decline it. That there were other options.
- That I didn't need it straight away
- Nothing
- Its accuracy, need for constant monitoring
- I knew all i needed to know for a hospital birth
- Yes
- Yes

- That you can decline it.
- I wish i given time in labour to make decisions
- The irregularities in the heartbeat
- I was told mostly
- I’m fine with what i knew.
- I wish the policy was not continuous fetal monitoring
- Whether there were other options
- Options, what they are looking for
- I wish I had have been offered wireless CTG earlier to stop the interruptions
- Why they were doing it
- The different types and why it is needed
- Nothing additional, I was well informed from both my antenatal classes and doing an online course with Dr Robyn Thompson which covers monitoring and reasons for/against it (and your rights as a mother in labour)
- I wish there was a wireless option available. Other than that, really can't fault my care team.
- What kind of monitoring I would be able to use and why I may not have been able to use wireless monitoring
- Yes
- That it was voluntary
- If there are any risks
- That the scalp clip is actually a screw in to the babies skull
- Options
- I would have liked to have been able to use the shower for pain relief
- That CTG unnecessary
- I wish my voice was heard, my babies arrived safely but my experiences could have been better if I was allowed pain relief and movement
- Risk of not monitoring, impact on being able to move
- Wireless option
- I wish I had been given the option for intermittent monitoring.
- Just information related to all continuous monitoring options available.
- Its always your choice and i wish the midwife respected my choices without throwing the hospital policy line at me
- I wish I knew that the FSE would possibly come off, and that my position would affect the connection.
- I wish I had of been properly informed that I wouldn't be able to move around. I consider it to be a large part of why my induction failed
- That I wouldn’t be able to birth in the bath.
- That there was a choice of options
- That it would be a non negotiable so I was mentally prepared to labour on a bed
- I had the information i needed
- Nothing
- Choice
- I was.
- I wish I'd been told honestly about what evidence is in the literature of it's benefits/risks, I wish I'd been empowered to know that it was my decision to make, I

wish I'd been told just how much it would restrict my movement, I also now feel uncomfortable learning that the trace is being transmitted to another room with people watching who are not my caregivers.

- Not necessary at all in most cases, No real benefits, more a hospital policy than anything else
- Nothing. While I don’t remember being given much information I felt happy and comfortable with the information I did have
- Limitations
- That I could refuse it
- I was happy with the information I was provided
- That it was my choice to be monitored!
- Why they were doing it
- Risk of intervention, statistically doesn’t improve outcomes in most situations
- Nothing
- Just more prepared for how annoying it was
- That I had choices.
- What all the different lines of monitoring are and why there is a need for it
- Was happy with what I was told
- The fact continuous monitoring and being strapped to a bed is painful, higher c section risk and doesn’t change outcomes
- Nothing different to what I was
- How restrictive it was, the limits it puts on where you can labour (eg- in water), how unreliable and difficult to adjust they can be, how they often don’t pick up what they’re meant to due to poor positioning, and that it’s not mandatory
- I wish that stick ons would have been available so that i was disturbed by others touching and fixing the straps or hearing the sounds if the machine right next to my head
- What all the monitoring options are
- N/a
- How much the risks outweigh the benefits in many situations
- The implications for Ctg- especially for wired model, increased risk of leading to C-section, limitations of Ctg with positions
- The reason why they’re were doing it which may have led to me knowing that the syntocinon would ultimately end up putting me into an emergency c section
- I wish when they promised I could move around they hadn’t actually lied
- That I wouldn’t be able to freely move around to help baby engage and manage pain, and that wireless doppler options were available
- Just because the monitor shows a heartbeat, it doesn’t mean she’s ok.
- That I had options
- Greater explanation of why it is required and the different monitoring options available. Also how to interpret the results of the monitoring
- I wish I was given the options of which types of monitoring are available and understood that I also had the choice to decline monitoring.
- I wish I had discussed if wireless monitoring was an option. I knew monitoring was necessary due to complications but would have like a more active labour process. However, in hindsight, this wouldn’t have changed the outcome of having an EC.

- I feel like I was informed enough. I just got caught up in the momentum of medicalised intervention that I felt was prioritised over my instincts as a birthing woman. Next time I will be more confident to apply my knowledge and decline monitoring if I feel to do so.
- When it is necessary and the benefits of it in my particular circumstance
- I had an epidural during monitoring so could not move around. I found the monitoring comforting
- There was options that are better for the patient than the doctor and midwife- staff choose options that are most convenient for them
- My body told me my baby was fine and safe, everyone around me told me my baby was going to die without a c-section based off being unable to find a heart rate on monitoring. I wish someone told me to go with my gut.
- Anything
- That heartrate is only one way to check on baby.
- Choices, options
- Not much really. I don’t think it’s that important to be told anything about monitoring. They need to do it and they hook you up. If the straps and pads don’t work they use another method. Monitoring heart rate is important and I think necessary
- That there might have been cordless available
- That i had a choice, one i wasn’t given in labour
- Nothing about monitoring, more about mindset and the increased risk of c section after induction
- No mobility
- I have no regrets about what I was told or not told. There plenty of other more consequential things to be thinking about (pain, pain relief options, baby’s health, whether I would need interventions) without thinking about the monitoring. I was happy to have it, as it reassured me that my baby was okay and that if her heart rate changed we would know about it immediately.
- I think I was told an adequate amount. I don’t feel like I was given any choice on it though.
- The restriction of movement! too much fiddling, the distraction
- That there may have been options available rather than just cords and wires
- It didn’t make any difference to me - I felt lucky to have the reassurance of monitoring
- I’d had second daily ctg monitoring for 5 weeks prior to my first birth, and a week before my second so I was familiar with the ctg machine. I wish I had been more vocal in insisting upon wireless monitoring for my first birth.
- That it’s normal for baby’s heart rate to drop in labour. Every little dip caused an absolute panic and I was stuck on that little screen. I couldn’t progress or relax, I felt so constructed and uncomfortable as it triggered my sensory issues
- That there were other options besides cord machine
- Nothing, but I wish they’d be a little less strict with it given new research
- Nothing specific comes to mind for my experience
- That you could be attached to a machine for hours and be unable to walk around
- Nothing. I knew the device was idiotic before birth.

- I think i was informed enough, just wish i did not have to be induced in the first place
- Yes
- I wish i was advised of wireless monitoring. As I had wired monitoring i felt like i couldn't move and had to give birth on my back
- Nil
- Nothing.
- I wish I'd been told the extent to which it would inhibit my movement more clearly
- The truth about the lack of evidence in the literature that it has any benefit!
- That it's restrictive and invasive

5. Do you have any other comments you want to share about your experience of monitoring during labour and birth?

-
- Being induced I felt I needed to be monitored but that wouldn’t have been the case if I laboured naturally
- N/a
- My experience in a rural base hospital was 1000% better than a local major city hospital. I felt the midwives were more used to natural, intervention-free births and chose monitoring that supported that, vs the city midwives who assume everyone is having an epidural or will need one.
- Admission CTGs should not be a thing for women without risk factors. Intermittent monitoring is sufficient and should be the gold standard. I hated being stuck on the bed attached to the monitor
- I would be very interested to know the EVIDENCE behind continuous CTG monitoring being a standard part of most labour ward procedures.
- No
- I feel like I got duped into wired monitoring as the wireless ones "ran out of battery" after a couple of hours. I was not given the option to take it off for a bit (eg to go to the toilet) and the midwife seemed annoyed by my frequent requests to be disconnected to use the bathroom. Hardly a reassuring experience, rather dehumanising to have your normal bodily needs treated as an annoyance, at any time but especially during labour. Also, It does not appear to be considered as an "intervention" by most people, despite having a large effect (mostly negative as far as I can tell) on experiences and outcomes.
- I was monitored for both of my births. In both cases it was a surprise. I was not told beforehand. I didn’t know I had a choice to not be monitored. I assumed everyone had to have it. Being monitored really negatively affected my first birth. It made me feel out of control and like I was only allowed to lie still on a bed through contractions. I was more prepared with my second birth and kind of just ignored it and did my own thing but I still had a midwife fussing over me like every 30 mins trying to adjust the stupid thing is it would slip
- I didn’t progress beyond 7cm. I was never told if my baby was posterior. I wonder if monitoring would’ve told them that?
- It gave caregivers something to "rely on" when discussing what went wrong during our birth.

- Women need to be given options and evidence based reasons for CFM.
- No
- I feel conflicted about it - my eyes were glued to the machine for my 27hours of failed induction, which made me incredibly anxious. But it also showed clearly how my baby wasn't coping and was progressively deteriorating in condition.
- No
- I was given morphine early on in my labour and felt completely outside of my body afterwards. From there I don't remember ever being asked if I consented to monitoring or how I wanted to labour. I think we all thought the labour would stop as it was so early. Like I said previously it's lucky it was a quick labour or I would have been very uncomfortable strapped to the bed.
- It completely altered my labour and birth. No one cared about me or trusted me, they rushed to the machine. I was hooked up so I couldn't move. I was strapped to the bed and ignored.
- Sorry, I'm not sure if I've helped your survey at all. I felt the questions were a little skewed towards trying to show the benefits of wireless monitoring, but my experience with previous pregnancies was that I just used what was available, and the wires weren't really a hassle. It is what it is, I guess.
- Continuous monitoring cannot be necessary when safe births occur without it. I think hospitals pursue it to protect the hospital not to support mother and baby.
- As I transitioned my water broke, heart rate wasn't able to be monitored and it lead to them wanting to use forceps, they did an episiotomy without me being able to consent, but I pushed my baby out before they could apply forceps
- It's debilitating, it takes choice away, makes medical professionals make snap decisions based off of a machine, no trust in the woman, it was a horrible experience
- My midwife changed birthing suites for me because she knew my desire to be more mobile during labour. The first suite was supposed to have wireless but did not. They started hooking me up. Thank goodness we could move suites. I think more pain relief/intervention would have been necessary if I was not able to move/be in the shower.
- I forgot to say i had an epidural as well
- Subsequent births without continuous monitoring were a much better experience and outcome for both myself and baby. I wish I had been more informed and known that I could have refused the continuous monitoring in my first labour.
- The straps were annoying when wet from the shower
- It was my way or the Highway!
- No thankyou
- I was not told enough about the options. That you can decline if you wish.
- I would encourage women to become informed. Monitoring is not just about hearing your baby that is just an added bonus
- A few times I wished if the heartbeat was normal and sounding healthy that someone would have been a bit more vocal in telling me that to reassure me in the middle of labour! I know nobody wants to disturb the labouring woman but if they smiled gently and nodded that everything was OK I didn't see it, i even asked a few times of the heartbeat is it OK!?! And it's hard to think thoughts and speak in the middle of pushing out a baby.

- No.
- I’m not sure if it’s relevant but I felt like if I was older and married the team of midwives/obs would have made a better effort in explaining things and asking consent, I felt very infantilised and I wish I was given to opportunity to decide what to do with more information
- Mostly a positive experience. Definitely could not consider consenting to monitoring that would not allow freedom of movement. Movement and water were essential to birth for me.
- I felt the hospital used coercion to get me to use CTG
- Loved my private midwife. The hospital midwife’s are understaffed, overworked & hog tied by a breaking system not designed for women
- Very much affected both my VBAC births due to policy restricted my options and my desire to shower etc which wasn’t permitted
- I was not asked for consent with monitoring, just told I had to. I was strapped to the machine unable to move around. Had to wait for assistance me to even go to the bathroom
- There definitely needs to be more information given to pregnant women explaining all their options
- I’m glad I declined CTG monitoring & opted for Doppler
- Put it all there in the previous comment!
- It seemed more about midwife ease than my comfort. If it were to be unplugged and moved so I could be comfortable where I wanted to be it was apparently a lot of work for them, so I just had to lie in the bed uncomfortable. It was like yes I could make you more comfortable but it’s too hard with no other options.
- My second birth had much better wireless monitoring and it was a completely different experience. I didn’t notice it and was able to shower/ bath and roam free
- Monitoring is what picked up my baby’s bradycardia which is why it ended in an emergency cesarean so I don’t know what to think - whether it potentially saved my baby’s life or caused me to have another unnecessary cesarean
- I’d say the best kind of monitoring if a woman need one is intermittently with her permission using the Doppler
- There is fear connected to monitoring and fear is the enemy of natural birth.
- No thank you.
- CTG trace was the sole reason for my failed induction leading to emergency Caesarean section. Since then, I have had three independent midwives review my hospital notes and advise that they would not have suggested the same course of action, rather that there was nothing wrong with my baby
- No
- Yes, more wireless monitors need to be provided in hospitals it has to be available to every woman
- It was horrible. I didn’t know I had choice, staff were verbally forceable that it had to be done but it wasn’t properly discussed and I wasn’t given the opportunity to make choices. I wish my partner had also had it explained to him so he could have discussed it with me too and we could have made a decision together without the pressure of medical staff. Felt like I was taken advantage of in a vulnerable situation and not given choice and control

- Monitoring for me personally I enjoyed and it was reassuring for me. It did add extra stress knowing what I was hearing and seeing though I'm sure that is the nursing background. I also have a history of tachycardia so it was reassuring to be able to see my heart rate, until it was quite high especially during active labour. It can add extra stress. It would maybe be an option if the sound was able to be muted inside of the birthing room or the computer screens had a family mode etc where there is a screensaver instead of the monitoring screen.
- I think my experience is warped as a midwife. I already had relevant clinical knowledge, but being on the other side as the woman has encouraged me to be far more thorough in my explanations and holistic prior to gaining consent
- I would have appreciated being asked if a midwife could touch me before shoving the hand held monitoring on me. I did not give consent I was t given a chance to.
- Second birth less monitoring which was a lit less stressful and helpful for labour to process
- I think hospitals over medicalise birth, the practices aren't evidence based enough to warrant a lot of interventions that are done and women should be given options and supported rather than forced into their policies

- We need better solutions for monitoring posterior babies to avoid fear mongering from medical professionals. Once we got the scalp clip in we could see clearly that my baby was fine - but we had to break my waters to do that which was excruciating and hindered natural labour. If the scalp clip or a CTG that doesn't work are my only options with a posterior bub, that's not good enough.
- I think the healthcare providers try to use scare tactics to get you to make decisions during birth or don't give you options and take the choice away. In my consent for VBAC it stated that CTG may need to be used but at my appointment the obstetrician has told me it's non negotiable.
- I think telemetry worked well for my size and the fact baby was breech - no loss of contact etc (I was lucky)
- Continuous monitoring is trash and so are the people who continue to peddle it
- They shouldn't force monitoring on ladies unless there is a real health concern of baby.
- As much as Choice does come into it, hospitals make their own policies which makes it hard
- Earlier monitoring may have been the key to preventing a brain injury for my child so earlier monitoring should be considered.
- Wireless and water proof monitoring is important to have available to allow freedom to move and use the shower.
- It was a horrible experience and learning it was potentially unnecessary and if my care provider would have listened that me declining meant they should have been taken off was incredibly traumatic for me given the outcomes that happened following
- Yeah this is my third pregnancy. Monitoring in the hospital setting for the first two was horrible. They used the belt and clipped the ctg to my stomach and it was

constructive and painful. Using the Doppler this time was the best experience thus far.

- WHO guidelines recommend against routine monitoring on arrival, best practise should be standard
- I am a midwife. This impacted my level of understanding
- I work in a hospital environment and I am an assertive person however I didn't feel strong enough to say no to constant monitor. I hate to think about women who don't have this background as it would be even harder!
- No
- My first birth was not a great experience but one off the things that still stands out in my Memory was how much I hated the midwives pushing on the monitor so hard into my belly trying to get reading. I had imprints in my belly for hours after.
- Intermittent monitoring meant I was able to move around, use the bath and shower when I needed to, change positions, and give birth naturally with support from the midwives and my husband. I value the skill and professionalism of the midwives in reassuring me through my labour, and helping provide a safe space for me to wait til the waters broke naturally rather than using an intervention method.
- Keep electronics and doctors out of the birth experience unless absolutely medically necessary!!
- Doctors and midwives need to keep their hands off birthing women
- None
- The hospital system is abhorrent. The coercion and bullying needs to stop
- Increased intervention due to being in one position on the bed
- I had some time on the Ctg during which the midwife couldn't make it wireless, so I had to wait for the shift change. That part was very distressing to me as I had been reassured that I would be able to move freely and instead I felt like I was trapped not just in the hospital but within a metre or so of a machine.
- No thanks
- I would not opt for the monitoring again. I think that a hospital policy for CTG monitoring simply because you are post dates is unjustified and should be offered as an option for the birthing mother more than a requirement
- No
- It's really difficult to undo that power imbalance between women and doctors/midwives, and doctors and midwives need to recognise this and be very careful that their language does not support that imbalance.
- I was in early labour at hospital when they did and wouldn't let me go home to rest and wait it out because the baby's HR was up due to the monitoring. I would have much preferred to be at home for the most part of my labour but had to stay in hospital being monitored
- I generally felt bullied throughout the labour and birth of my first child and the monitoring played a role in that too.
- I believe my experience was one of the best, to be let be most of my labour without beeping machines or being confined to a bed for monitoring. I feel more women from all risk categories should be shown all options and have free choice for their labour and birth experiences. There is way too much fear in the maternity system

these days and birth has been happening for millennia without such monitoring devices. Don't feed women fear!

- Monitoring saved my daughter from injury or worse and I'm really glad we had a CTG. I can see how that might be restrictive or invasive for other people though.
- No
- My labour was very quick so there wasn't much time to listen to the baby before they were born anyway!
- No
- I was able to move freely, and spent a lot of my labour in the birth pool. I wouldn't have wanted to be anywhere else, for sure.
- The external CTG is a bad (flat) shape and continually list contact with the baby's heart rate. The FSE was less invasive because I didn't have a midwife constantly in my space trying to find the baby. However it excluded me from using the bath.
- No
- It made me stuck to a bed my whole labour, i wasn't able to move freely and i wasn't able to access water in the shower or bath for pain relief. All hospitals should have every monitoring option available for birthing women so we can choose what is suitable or wanted during OUR birth.
- I declined CTG based on my personal situation and knowledge. I was birthing at a rural hospital that I work at and was told I may have to transfer to a tertiary hospital for birth if I declined CTG. The OB wanted consent prior to labour, I didn't feel comfortable making that decision prior because the clinical picture could change. I was lucky to get a beautiful midwife who respected my wishes and didn't try to change my mind whilst in labour
- I hope technology will develop further to allow women to labour without any sort of restrictions imposed on their labouring experience.
- My midwife was integral to my positive experience of monitoring, my desire to continue to move my body and focus inward, and the monitoring offered to me allowed for this.
- Having a screw put in my babys head was horrifying.
- The MGP programs (run by midwives) is 100% recommended by me. Having the same midwife for all my births keeps the same continuity of care as well as a deep level of trust between you. Highly recommend!
- This was my 4th baby and the most uncomfortable labour due to the straps used for CTG monitoring
- I wish more women knew about their right to choose| decline and the risks of monitoring as well
- No
- Midwives were awesome and were very receptive to the way I wanted to give birth.
- Having had 3 long labours before caesareans- I can guarantee they contributed to me having my caesareans- and with 3 babies with Apgars 9,9,9 even at Caesar- the monitoring is bullshit and causes more unecessarians
- Yes, it was made out to me continuous monitoring would be distracting and annoying when it wasn't at all. I liked being left alone, knowing baby was safe and not having my breathing interrupted during contractions

- It helped my peace of mind to know me and my baby were monitored so if something went wrong we could act and do something about it
- Only monitored at the end when meconium in baby sack
- I was very prepared for all pathways in my labour and had a plan for most things but I think I viewed monitoring as a very low intervention option. I think next baby I would have a different idea that it is a big distraction so only use it if absolutely necessary. Distraction and loss of focus can so easily slow down/stall labour and that just leads to more intervention
- Yes, being hooked up and monitoring made the experience so impersonal and medicalised. Not a setting to induce the natural hormones required for a physiological birth or anything near it.
- Would like more options for FHR monitoring, something less restrictive
- babies move! And because of this, monitoring can cause unnecessary stress!
- Depictions of women undergoing continuous fetal monitoring on TV/ movies are very detrimental in terms of social understanding/expectations of what a normal, active labour should look like.
- Yes. My midwife asked for consent everytime to listen in. And i occasionally said no and that was respected. Yay to her! I don't think in a normal labour its as important as people make it out to be.
- Monitoring moves the focus from women centred to machine/data centres care. Monitoring with an epidural makes sense (my second birth I had continuous monitoring because I couldn't identify if I was having a contraction), but for spontaneous birth without epidural it was not beneficial, and contributed to poor care.
- Monitoring of bub shouldn't take complete priority over the mothers ability to labour comfortably. I believe that the c-section may not have happened if i was able to focus completely on what my body was doing, but this was not the only contributor to it in the end
- Private OBs tell you one thing during your appointments and make it sound like everything will be as natural as possible and they will respect your wishes. This is a load of crap. Constant monitoring was one of the factors leading to a traumatic birthing experience. Never again.
- Trauma including sexual assault by a doctor after screaming at him to stop cos he was hurting me, he didn't.
- I am all for fetal monitoring in labour. Birth is hard for the baby too and so many things can go wrong even to mothers deemed low risk
- No
- I had internal monitoring. Wish I'd known it can leave a scar on my babies head
- Just glad the Mater mother's don't seem to use the screw in baby's head anymore much less invasive for mother and allows her to focus on birthing
- It was a big, a huge part of what went wrong on my labour, the maternity health in Australia is totally broken, it has damaged my life forever
- Wired did add an element of can I get up?
- I was always happy to have intermittent monitoring in labour. When my waters broke and I went in to hospital with meconium liquor I was told CTG would be advised, and also due to having COVID. I agreed to this and got in the shower. I was

getting harassed to move into better positions during contractions so they could get a better trace of his heart rate. I asked if his heart rate was okay and they said it was a normal trace. So I took the CTG off and asked to go back to intermittent monitoring which they agreed. I asked if I could have a scalp clip instead if I needed continuous monitoring later on in the labour as I didn't want to be disturbed in the shower. They told me they were no longer an option if I was in the shower. I asked why this was the case and the midwife said her manager told them they couldn't get wet. I asked her to check the box for manufacturers information and all it said was that there is no evidence on safety for water immersion. I wasn't getting in the bath. I told the midwife if I needed one she could put one on and document that I got in the shower against advice. Anyway I never ended up needing it. I agreed to syntocinon augmentation toward the end of labour and to have the CTG put back on for this. As I started to approach second stage/ urge to push I could feel baby moving a lot and rotating down. The heart rate was elevated on the CTG and I could feel they were worried, hovering over the CTG looking at the paper so I told them I'm not worried about my baby, he's just moving a lot. he then started to have early decels with contractions which recovered in between. So I assume because of this they started directing me to push and telling me I needed to stop holding back, and putting a lot of pressure on me to get the baby out. I knew this was normal in second stage and could still feel baby moving. I kept saying I'm not worried about him and trying to follow my bodies urges but they kept directing me how to push and wouldn't stop pressuring me with it. I really didn't like it. Once his head was born and I was waiting for the next contraction they were even trying to tell me to push before the next contraction. He came out perfectly fine, cried straight away. I wish they had have listened to me.

- I think i did in the previous question but i think every single person who is involved in births should be reminded of what INFORMED consent it. We should be given ALL information when making decisions not be told this is what I'm going to do or we need to do. Just because monitoring is wireless does not mean its not restrictive.
- If I had my time over I would of like the option to decline the monitoring. I was told they wouldn't except me to birth there if I didn't accept it. It was very distracting and uncomfortable and I wasn't allowed to use the shower as a way of natural pain relief as they couldn't hear it properly. I ended up with an epidural because I wasn't able to movement and water the way I wanted to for a natural pain relief. It just stressed me out and I gave into the nurse who suggested a number of times that I should have an epidural and I feel she was doing that to make her time easier rather than helping me with the way I wanted to labour
- I didn't realise that going over my due date would require me to have so much additional monitoring. I was a low risk pregnancy, and each monitoring appointment apparently was normal, but I still had to come back every 2-3 days, then daily once 40+7. Felt like the medical intervention started with the regular monitoring, thru to (failed) induction, failed spinal, GA c section.
- No thank you
- No
- The experience of monitoring raised my anxiety while providing reassurance. It was a complex experience.

- Hated it. It ruined me. I had cannulas in each hand and couldn’t move without the monitoring beeping because it had lost the heart rate. Baby was perfect, it was the machine. They changed machines about three times. Never again.
- My experience of monitoring felt like I was coerced into an intervention that I didn't really want because I was in a vulnerable position
- no thanks
- The push for CTG without an evidence base was fairly concerning. I had a great birth and I think intermittent as opposed to continuous monitoring was a part of that.
- looking forward to hospital care providers using evidenced based care and not relying so heavily on CTG. Also looking forward to hospital providers gaining informed consent but I don’t hold out much hope. Informed consent is severely lacking in public and private hospitals. And the RANZGOG are a patriarchal joke who peddle the status quo
- There needs to be a more accurate way to monitor babies in labour. And reasons for a CTG in labour reexamined
- I think we need to present more options for women. And if it's possible to cordless) wireless monitoring if it has to be continuous.
- I’d like the norm to be to provide women with information so they can make an informed choice about their birth.
- Very positive and supported
- When would arrived the CTG was put on. I knew how CTG can contribute to the cascade of intervention. A soon as there was a reassuring trace I asked the midwife to take it off a so wanted it on as little as possible. She then did Doppler monitoring for a few hours. I think this is not utilised enough. If there is a risk factor put it on for a while but if it’s reassuring then take it off for a bit
- I had a private midwife who offered me intermittent auscultation or if I wanted less than 15 minutely checks. I opted for 15-30 minute checks
- it is not discussed / explained well during pregnancy
- My first birth was pretty good, the midwives were pretty switched on. My second birth I had a midwife from a different area and she wasn't as sure about the equipment which didn't inspire a lot of confidence.
- Nope
- We need to improve monitoring options at private hospitals
- It took 3 attempts to internally place the tether, this would be the opening ceremony to a very traumatic birth. Lost confidence right from the start. It took three attempts to place because it wasn’t picking anything up, they hadn’t turned it on. That was the only reason it wasn’t working. 4 staff didn’t realise it wasn’t on.
- Having machines that actually work and are ready to go when I arrive at the birth suite
- None
- I wish more women knew they didn’t have to consent to constant fetal monitoring
- I’m not sure if mine technically counts as during labour, because although we tried several options to get my body to go into labour, I only experienced an hour or so of light contractions. I also really appreciated the monitoring they provided through the pregnancy for decreased movement checks.

- Monitoring is not necessarily consistent throughout. I did have wired for a part of it but mostly wireless
- No
- I would do it again for my babys health
- Nope
- I was also attached to a drip which also limited movement
- No
- I may have made peace with it but i still feel angry about my labour.
- No
- Constant wireless monitoring is not comfortable
- It's important for women to know that they have the power to consent to monitoring of any type
- It made me more anxious
- The wireless machine ran out of battery and therefore I was not able to use it
- Please choose midwives carefully. Some just shouldn't be midwives, they are vile and the bed manner makes or breaks this experience
- I think it would have been useful to understand the purpose of monitoring, options available and how to react to alarms
- I had an extremely quick labour - arrived at the hospital at 10cm, ready to push. the monitoring had no noticeable effect on me or baby
- The reason I was monitored was to give the midwife reassurance as my labour occurred outside of a maternity setting in a remote area, but it was unnecessary and impacted upon my labour experience
- I feel there is way too much intervention in women's labours and births, it has made it so sterile and clinical.
- Changing positions after each contraction for the Doppler was very frustrating as it limited my movement. When the ctg and scalp monitor was on it was disappointing being lied to that there was not a problem with the babies heart rate when I could hear very obviously that there was (my husband and I are medically trained)
- I was very fatigued. I didn't have energy to move around so doubt i would have asked for wire monitoring
- My priority was a healthy baby. I was happy to accept any monitoring or management needed to ensure a healthy baby on delivery
- My child nearly died because the staff kept fobbing me off.
- Became concerning when care provider focused on trying to locate baby's heart rate instead of providing reassurance that this is just one tool on a bigger picture.
- Modern medicine is fantastic, but there's a time and place when it comes to labour. I think monitoring was right for us given our circumstances
- My labour was only 40 min whilst in hospital so it should be noted there was very little time for monitoring
- Na
- Hospitals need to treat the patient not the procedure. i did not NEED to be monitored continuously but my preferences were dismissed because of policy
- The wireless monitoring is great since i don't remember i had it, but i definitely remember the ob keep reading the heart rate of the baby. And i was informed the baby's heart rate was dropping at some stage.

- Make things clear laboured October 2013 no monitoring, laboured February 2017 water birth so was monitored a handful of times but this was explained a few times during pregnancy so I was expecting it. Laboured again in August 2019 no monitoring at all. Laboured February 2022 and was monitored between every contraction. This labour I had a student midwife wo struggled to find the heartbeat which the first few times made me worry something had happened but quickly realized she wasn't good at finding the heartbeat. She would sometimes take multiple minutes attempting to find the heartbeat, wouldn't stop or move so I could walk through my contractions, am allergic to painkillers and she was aware of this I told her waling was my way of coping with the pain but she rarely stopped looking for the heartbeat during my contractions. Her looking for it hurt my stomach skin (which is covered in stretch marks and is super sensitive). I was not told they would be required to monitor the heartbeat and was never told why it needed to be done. Having her try to find the heartbeat while I was trying to walk around or walk on the spot just annoyed me and I had to stop myself from yelling at her to go away and leave me alone as I was absolutely sick of her checking the heart beat.
- No
- I feel that monitoring impacted on my overall satisfaction of my labour and delivery.
- I was completely happy to be continually monitored, it was what was best for my child. I was just annoyed that I was literally trapped and confined to a bed
- I believe that it's better to have intermittent monitoring unless it is absolutely necessary for the mother and baby to be monitored the whole time.
- Monitoring technology saves babies lives and should be easily accessible in all hospitals.
- I believe monitoring saved my babys life - very important to me!
- Overall it was a good thing because it resulted in an emergency c section and live birth rather than a stillbirth
- I feel sorry for anyone who wasn’t given details or choices and had monitoring that negatively impacted their birth - but for me monitoring was standard, wireless and a reasonable request / part of giving birth in a private hospital. I think I paid for the service and would do so again. I believe it is needed for the safe arrival of baby and care of the mother.
- More information for parents prior to birth is a must
- No
- I didn't see the option for fetal scalp monitoring on the list so have answered up until the point I had the scalp monitors attached. I had these with both babies as they wanted more accurate monitoring of babies condition
- it sucked
- Monitoring enabled midwives and obstetrician to identify my baby was in distress and opt for an assisted delivery in theatre with forceps / episiotomy which was a good outcome for both of us.
- No
- We need to put more trust into women and start listening to what they want from a birth- too many mothers are experiencing birth trauma and the medical profession wonders why.

- More compassionate staff in explaining and equipment that works. The wireless kept losing the connection and being able to use them while freely moving around with a secure connection would have been much better. I still had a positive labour experience though.
- It took one of the most unique and possibly only birth moments I could have had and tortured me for hours on end needlessly due to medical abuse and the lack of informed consent.
- It sucked
- Being able to visualize my toco was off putting because I would begin to tense when seeing the number rise
- It saved both my babies, very thankful for constantly being monitored. I hope everyone can receive this
- No
- Shouldn't be used unless absolutely necessary
- The use of monitoring was probably more implied consent than direct consent. There was no sense of force by medical staff, more so just routine and procedures.
- I am obese so the machines were harder to pick up baby's heart beat

- Ultimately i had a healthy baby at the end of my birth experience so i am so grateful for the medical help i had
- Continuous Monitoring is incredibly over used!! There are far to many contraindications to intermittent monitoring, and too many indications for Continuous. The threshold is too low. And women aren't educated that their risk of a c sec is higher with cont monitoring.
- Monitoring mean i could see the baby was fine, which kept me relaxed despite the marathon labour.
- Birth trauma caused by being unable to move, stuck on my back on a flat bad
- Continuous monitoring was more of a hindrance in birth than a help. My hypno birth was calm, my baby and I were healthy and it was purely to satisfy a doctors need to be in control.
- I feel like it was policy for a premature birth, I was fine with that. If I don't have a prem birth this time it will be great to not have monitoring attached
- I appreciate the technology is available to help when needed. I think midwives need to be trained better in how to set them up and use them, but ultimately spend TIME with the woman in labour and find out what is going on rather than read a chart. Use technology that can be put on and removed quickly so the woman can focus on her what she is there to do - deliver a baby as naturally and safely as possible.
- No
- No
- Midwives, doctors, nurses need to trust womens bodies instead of trying to manage with interventions and monitoring. Care should be truly women centred and undisturbed
- Is this a requirement?
- The midwife was great and supported my decision for intermittent monitoring but I know this is not all midwives/doctors. I'm so glad I didn't have to fight for what I wanted during labour.

- My midwife was great and didn’t make too much fuss about the monitoring when I needed to change positions and it didn’t give a great reading. Nothing alerted her to stress if the baby at that time, so she let it go until I changed position again and could get a good reading again.
- I was on CTG for approximately 10 hours and when my baby was born, i actually cried because I couldn't hear her heartbeat in "surround sound" anymore
- It was helpful when deciding how long I could keep trying for a vaginal birth before baby was in distress
- Prefer to have not been monitored and drip (syntocinon)
- For me all I wanted was baby ok, so hearing the heart beat gave me reassurance. One that starting getting lost or dropping it was scary though, but I'm glad the medical staff knew it was happening so my baby was delivered alive as they knew the heart rate was dropping
- Thankfully I was monitored as baby was distressed resulting in an emergency c-section
- Overall it was a good experience to be monitored
- I read my body perfectly and had a beautiful birth with a healthy happy mother and baby despite the distracting and interfering midwife who witnessed. My primary midwife was my advocate for my choices and made the difference
- I liked being monitored, it was reassuring for me.
- There is a better way. Women can feel empowered by being given choice and control over their own bodies. Continuous monitoring is not the best option for everyone
- I was left labouring in a maternity ward room with a woman and her baby a metre away from me as I was not permitted to go to the birthing unit until someone was available to monitor my baby. This was uncomfortable and ultimately traumatic as I felt that I needed privacy for my labour. Then to finally be allowed into the birthing unit only to be strapped to a machine rather than given access to the birthing tools led to my need for an epidural, then a forceps birth as I couldn't push effectively. So basically, monitoring ruined my labour and birth experience and led to interventions I didn't want and 4th degree tearing.
- I’m glad I had read up on my rights to not have some much intervention as well as the support of the midwife team to make me feel safe and feel that baby was safe
- Just an added stress when so much was out of my control and wishes.
- No
- There is a need to stop the cascade of interventions when it comes to birth. 1 in 3 women experience birth trauma and it is becoming normalised. If induction wasn’t so common place, continuous monitoring wouldn't be so common place.
- I also had a fetal scalp clip
- If there are better ways to monitor that are less invasive and restrictive, that would be so good. The beeping and the constant sense of being tethered to a machine, especially while having contractions, is a kind of torture where you feel trapped and in pain but you can't move freely to help you work productively with that pain.
- More info could be included in the antenatal courses but overall I have was happy with my wireless monitoring

- My babies heartbeat kept dropping based on the monitoring. My midwife asked to have a scalp prick done to check for lactic acid to see how baby was. He was fine. Therefore the monitoring sort of provided a false negative. My monitor kept talking down so the student midwife spent hours on the floor holding it
- Personally i found it reassuring that the doctor knew what was happening with both babies as well as me. I am fortunate to have had exceptional care from all involved.
- Doctors and midwives need to be on the same page. It was stressful when they disagreed about monitoring in front of us. Also, monitoring wasn't bad in short labour (6 hours), but more challenging in my longer labour when i wore the monitor for 14 hours.
- no
- It was painful and is often hand in hand with induction, sadly. It's almost relied upon by the staff, so things are let to go on for longer than wanted or should rather than trusting mums instinct. Things got bad because they waited too long
- Wireless was much better and comfortable then the wired monitors but the wired monitor seemed to be able to stay on baby's heart beat better.
- I was monitored due to hospital policy for being induced. The monitor later detected some fetal distress and an internal monitor was placed onto babys scalp which I'm grateful for
- I just think it is great to have the option
- Wireless should be standard to allow movement while still giving care providers visibility
- More credit should be given to the mother tapping into her knowledge of her body and baby.
- Need better standards around informed consent and need those standards to actually be enforced.
- I didn't like the midwife always looking at the numbers on the screen when she came in instead of at me. I started looking at them too and it made me anxious
- I am a neonatal nurse so was happy to have increased surveillance as I have seen how quickly things can change. I found the wireless monitors to be fantastic and much less cumbersome than a scalp clip.
- When they had attached the scalp monitor the first time it was not attached very well. I was told to be very careful not to have it pull out, and I was. The midwife then managed to pull in out by trying to change my pads without even waiting for me to be ready. When the doctors came in to reapply another one the midwife blamed me and said that I wasn't being careful enough. I then had to have a second scalp probe attached. That was unfair and I called her out on that with my partner backing me up. Thankfully the doctors were good about it. The second monitor the applied though they made sure was on so tight that when I went for the caesarean they couldn't get it off and they had to wait until they pulled him out to get the probe off. At the end of the day I am glad I was monitored because that could of potentially saved my sons life.
- It was a minor feature of birth process
- I think by continuous monitoring midwives then don't have to stay with you and can see other patients

- I’m so glad it was wireless so I could move around and have a bath. Gave me peace of mind that bub was safe and doing well.
- No
- I was switched from CTG to wireless/clip monitoring without being asked
- No
- Clinical practice should be guided by high quality evidence. Recommendations based on opinion have to stop, mothers need to be listened to and feel heard in order to feel safe.
- For me I did not notice it at all. I don’t actually remember it even being put on me. I knew I had it on but never needed it to be adjusted so had a positive experience. If it had to be continually readjusted then I think my experience could have been different
- I attempted VBAC with my 2nd baby. Was bullied by the doctor at my VBAC appointment for declining ctg when I was already aware of the risks
- Hated it! Being stuck within 1m or so of the machine was bell. Couldn’t move around to help get comfortable
- I would not agree to future CTG without medical indication from intermittent monitoring. Wireless is very important to me.
- It’s a horrible experience getting the monitor attached and it was also horrible seeing the injury to my babies head
- I felt the monitoring impacted my labour and was part of the reason why I had to have a c section despite my biggest wish for a natural birth
- I was very happy with having Doppler and being able to be mobile. I really would of struggled if I needed a ctg and had to be on the bed and I think it would of massively changed my ability to cope with labour.
- It was annoying for my first birth. I did have the trace with leads initially for my second but I was left in the room with it on with clear spikes during my contractions but because I internalise in labour I was lying there quietly and told I wasn’t in labour despite the trace. Two hours later I delivered my posterior oriented son. If no action is being taken based on the trace/reading then why bother doing it? It felt like it was done to buy time when they were short on staff that particular night. In fact, at 9 dilated the obstetrician checked with the senior staff for permission to break my waters because of the staffing issues
- No
- No
- Having a monitor was great as it meant that the midwife Didn’t have to keep checking, and allowed us a little more us time.
- Overall I really liked my experience and all the staff were amazing.
- Expecting mothers are not given much information regarding monitoring. The medical professionals need to ensure this is covered in depth as my labour could have been very different and my baby wouldn’t have had an infection on her scalp if I had been told of the risks.
- I am not a survivor of childhood trauma or sexual abuse but remembering how stuck i felt i can imagine could be triggering for those women in labour. The question then is, risks v benefits......is monitoring continuously a benefit or a risk to those women

- It gave me peace of mind the birth was quite difficult and it helped me to know baby was ok
- Found that the midwives were very hesitant to attempt to use wireless ctg, which lead to being restricted in bed for 18 hours on wired ctg
- In the end I went from monitoring with straps etc to it being attached to babies head. Had all the wires but at least it did keep cutting out like on my belly.
- Having been to the hospital for monitoring prior to birth I felt comfortable knowing what to expect.
- One and done. Pregnancy and labour is not something I ever want to repeat
- Consent for monitoring needs to be clearly obtained from the birthing mother and only after she is properly informed of the risks and benefits of monitoring. It shouldn't be assumed that she wants or has to have monitoring during labour.
- I believe intermittent Doppler should be standard, not CFM unless it's a high risk (genuinely high risk) situation.
- continuous monitoring was a major factor in a traumatic and ultimately unnecessary emergency caesarean that I spent years recovering from the birth trauma. I felt completely disempowered because the monitor was put on without consent and I was unsuccessful in getting it removed during labour. For months afterwards I dreamed I ripped the monitor off in the labour. I wish I had done this in the birth.
- Ni
- I considered myself a little informed going into birth, but i think once in labour i feel as though i lost control or understanding as to why monitoring was needed
- My midwife was an excellent advocate for me and explained pros and cons without the presence and pressure from the doc/OB. This helped a lot.
- No
- Negatively impacted overall
- Monitoring helped with my husband and I peace of mind due to our first birth, but possibly impacted comfort and ability to relax during birth
- No
- It stopped me from enjoying the natural beginning of labour and made the experience very clinical
- No, thanks.
- No
- No
- My first birth was a c-section, and I did not labour at all. I don't remember what type of monitoring I had. It was not something I thought I had any choices about. I think if I had not been doing my own research because I wanted to achieve a VBAC, I would have done whatever the hospital policy was, without questioning it
- I just wish we had better technology and time with midwives to educate us on it all.
- I had a midwife [mgp] and she gave me excellent advice and information about what monitoring and what it might mean if certain things happened through labour. I felt obliged to do it and looking back now, would probably had preferred to stop the interruptions earlier. There were too many people in the room trying to assist me.
- In my own experience, monitoring is great, especially if it allows you to remain mobile, however it should not fully replace physical examination and should be used in conjunction with physical exams

- I was thankful to be well informed, given choices and asked for consent as i know that so many other mothers don't have this experience
- CTG picked up my Spontaneous Preterm Labour, without it, I would not have had access to the early intervention required to continue my pregnancy and my child might not have been viable. CTG should be available in EVERY emergency hospital. It was not used and I was discharged home from emergency saying my baby was fine, movements and heartbeat strong. My obstetrician was contacted and insisted I attend the private maternity hospital for CTG and immediately recognized and intervened to prevent my early labour progressing. CTG in my birth was cumbersome, but allowed me to labour longer and birth vaginally, rather than being rushed for a Caesarian due to time pressure - it showed us we were fine even if the process was a little slower than desired!
- It was a negative experience for me as the monitor did not pick up the contractions for me meaning I had less contact with nursing staff and much later epidural that I would have chosen.
- Nope just more info would be nice before labour
- When I had a cesarean they told me my daughter was dying based on her heart rate. At my debrief I found out this wasn’t true but the nurses were not qualified to interpret the readings.
- Extremely uncomfortable to have CTG and anxiety associated with necessity
- I do not regret having continuous monitoring during both my labour experiences. It provided me with great reassurance at all times that my babies were okay during and as labour progresses.
- I wish that women were offered more choice and not made to feel terrible when they request options outside of mainstream.
- Limiting the movements of a woman in labour is very difficult, constant re arranging of straps was very annoying
- I was asked to switch to the monitor on baby’s head due to the monitor moving on my stomach which my partner declined. This was close to the end of labour and I was glad we felt comfortable declining it. My midwife stated that if she felt she could no longer read the monitor she’d ask again - informed but safe.
- It was painful
- It was awful, how am I supposed to feel emotionally and physically safe to give birth being tied to a bed? This element of my birth experience greatly contributed to my re-traumatisation from previous sexual assault. I feel angry now that I read more about the lack of evidence in the literature of it's benefits to women and babies. It feels like just another way to control my body and put the needs of Doctors before mine.
- No. Thank you
- Culturally inappropriate and culturally unsafe, I wanted water births for all 3 children and was told I had to be monitored for all which meant no water
- My baby had the umbilical cord wrapped around his neck twice and the midwife later told us they knew through monitoring. Maybe faster action in using that information
- No
- I'm so glad this research is being done because I think this can be improved SO much.

- I don’t think so
- Was happy that my complicated labour was managed well, my baby was delivered safely and my own safety was ensured (numerous complications for me)
- The equipment used to monitor me was not working as it should. As I have extra fat on my belly there were issues trying to monitor baby’s heart rate and so they used an instrument that attached to my baby’s head. The equipment also wasn’t picking up when I was having contractions so I was given a cord with a button to press every time I was having a contraction. It felt hard to focus on my labour whilst having to record my contractions.
- I also ended up having a scalp clip put on baby as the external monitoring was not picking up her heart rate accurately and I wanted to go in the shower at some stage
- Wanted to be able to move freely
- Currently pregnant and major factor with discussion labour for this pregnancy. Aiming for a vbac and doctors are adamant that I require Ctg from presentation to the hospital once labouring a
- Staff need more training
- I hated it and will not consent next time. I had no idea it existed before I was in the birthing suite and health professionals working with birthing women should be held accountable for not providing sufficient information
- I understand the focus on the baby but being strapped to a bed unable to move for the purpose of monitoring is not in the best interest of the labouring mother
- I have several comments but too lengthy to jot down here. Happy to discuss by interview.
- The first midwife put the monitor on me, the second (who was there for the birth) removed it, suggesting that it was unnecessary. This made me feel frustrated because I had been using it for hours "unnecessarily". I was much more comfortable without it on
- I had a good experience with monitoring. I was quite exhausted by the stage of being monitored so it did not bother me.
- Labour is already a challenge women shouldn’t have to request the best options to suit the situation. The care providers should assist in finding the best option without adding to the challenges
- Private midwife made all the difference for feeling like i had choice and options and that I knew what was best for me. The hospital staff were judgemental and pushy in what they wanted, for no obvious reason (bub was never in distress in my case, just lack of progress and the fact i was vbac meant they wanted to monitor more).
- Guilting and scaring mums into doing things by saying baby could die whilst mum is in labour is disgusting and wrong
- No
- No
- No
- I think a lot of the monitoring was basically what I was told as it was hospital policy.
- No
- It was reassuring to have the monitor attached, but it was uncomfortable, it kept falling out of the strap, my partner had to keep holding it in place, it really got in the way of being able to â€˜naturally’ labour

- For a long time, I struggled with memories of my sons birth. It wasn’t clearly explained why I required a C-section and if it was I was probably in a daze having been in labour for a long period of time, finally getting to 8cms and then being told I’d have an emergency C-section. It wasn’t until I saw the next birth clinic with my second child that the nurse talked me through what happened pulling out the monitoring information and showing me what happened to make the OB decide on the C-section. It would have been nice to have everything explained a bit more thoroughly at the time
- I felt like it was for the benefit and convenience of the midwives and not helpful for me at all
- I don't think the hospital coped very well with me being an older mother AND being healthy, this made them put a lot of pressure on me to achieve their desired outcomes which included monitoring. I doubt they would have let me go without monitoring
- Thank you for doing the study. In 2022 seems insane that these monitors are being used. Equally insanity of midwives when it slips off during labouring that they insist on punching it in to your belly, the worst pain...as if once you’re that far dilated they’re really going to be able to do any intervention if the machine has no baby sound anyway. Please get them banned or technology upgrade to something that monitors baby heart without interfering with labouring.
- No thank you
- I have had five babies, all different labours. Four out of five were hospital births with constant CTG monitoring. I never get like I had the choice to say no. Fifth baby was born at home with some monitoring from midwife. This was by far the best birth. So calm and relaxed and just as safe.
- My student midwife was incredible doing the monitoring. If it wasn't for her I would have needed a repeat c section I'm sure. The monitoring was annoying, and wireless want available du to other births, but I'm glad I was able to give birth naturally due to monitoring. Personally I would have done it without monitoring - and I say that as a scientist and statistician fully aware of the risks - but I'm glad I was able to due to the monitoring.
- It’s best for baby.
- It was overall a really positive experience - I do wonder how it might have gone without the continuous monitoring but also with a healthy baby it is easy for me to question now.
